# Radius additivity score: a novel combination index for tumour growth inhibition in fixed-dose xenograft studies

**DOI:** 10.3389/fphar.2023.1272058

**Published:** 2023-10-13

**Authors:** Nicola Melillo, Jake Dickinson, Lu Tan, Hitesh B. Mistry, Heinrich J. Huber

**Affiliations:** ^1^ Seda Pharmaceutical Developments Services Unit D Cheadle Royal Business Park, Stockport, United Kingdom; ^2^ Division Drug Discovery Sciences, Boehringer Ingelheim RCV GmbH & Co KG, Vienna, Austria; ^3^ Division of Pharmacy, University of Manchester, Manchester, United Kingdom

**Keywords:** drug combination, biostatistic, mathematical model, synergy, xenograft

## Abstract

The effect of combination therapies in many cancers has often been shown to be superior to that of monotherapies. This success is commonly attributed to drug synergies. Combinations of two (or more) drugs in xenograft tumor growth inhibition (TGI) studies are typically designed at fixed doses for each compound. The available methods for assessing synergy in such study designs are based on combination indices (CI) and model-based analyses. The former methods are suitable for screening exercises but are difficult to verify in in vivo studies, while the latter incorporate drug synergy in semi-mechanistic frameworks describing disease progression and drug action but are unsuitable for screening. In the current study, we proposed the empirical radius additivity (Rad-add) score, a novel CI for synergy detection in fixed-dose xenograft TGI combination studies. The Rad-add score approximates model-based analysis performed using the semi-mechanistic constant-radius growth TGI model. The Rad-add score was compared with response additivity, defined as the addition of the two response values, and the bliss independence model in combination studies derived from the Novartis PDX dataset. The results showed that the bliss independence and response additivity models predicted synergistic interactions with high and low probabilities, respectively. The Rad-add score predicted synergistic probabilities that appeared to be between those predicted with response additivity and the Bliss model. We believe that the Rad-add score is particularly suitable for assessing synergy in the context of xenograft combination TGI studies, as it combines the advantages of CI approaches suitable for screening exercises with those of semi-mechanistic TGI models based on a mechanistic understanding of tumor growth.

## 1 Introduction

Combination therapies are commonly used in the treatment of many cancers because they are often demonstrated to be superior to monotherapy, an effect often attributed to drug synergy (Mokhtari et al., 2017; Plana et al., 2022). Thereby, drug synergy can be defined as the effect that a result (e.g., an anti-tumour effect) of a combination of two or more compounds cannot be explained by simply both compounds acting independently. Drug synergy can be due to pharmacokinetic effects, where drug-drug interactions may lead to a higher effective drug exposure, and pharmacodynamic effects, where biological synergies on the molecular mechanism of action are exploited.

The study of drug combination effects from clinical data is restricted by ethical constraints that prevent systematic comparisons of control populations with single treatments. Hence, most of the understanding of combination therapies in cancer is derived from preclinical studies (Plana et al., 2022), such as mouse xenograft studies with implanted human tumors, where tumor growth and its drug-mediated inhibition have been studied. However, the predominant method for studying drug combinations for pharmacodynamic synergy is to use dose-response matrices of *in vitro* screens of cell line panels (Pemovska et al., 2018), which lent its conceptual framework to the analysis of *in vivo* and clinical studies.

Combination indices (CI) were used to determine whether co-administration of drugs showed synergistic (CI<1), additive (CI = 1), or antagonistic (CI > 1) effects. CI can be defined as the ratio between the expected additive effect of a drug combination and the actual (measured) combination effect on a certain *in vitro* parameter (such as the proliferation IC50, defined as the amount of drug needed to reduce the proliferation by 50%). Several approaches for deriving CI have been reported (Foucquier and Guedj, 2015; Pemovska et al., 2018) and differ in the calculation of synergies and assumptions on drug-effect curves. Therefore, the response additivity score assumes linearity in the drug-effect curves (requiring synergy to exceed the added single effects). Conversely, the Bliss independence model assumes an exponential drug-effect curve, and the effects are treated as probabilities between zero and one. By contrast, the Loewe additivity score evaluates whether an excess synergistic effect cannot be explained by a mixture of both compounds acting independently (Foucquier and Guedj, 2015). Unsurprisingly, different concepts often yield different CIs for the same data (Pemovska et al., 2018).

To explore the combination of CI-based methods in studies of drug-mediated tumor growth inhibition (TGI) with fixed-dose xenografts, limited literature is available (Wu et al., 2012; Wu, 2013; Huang et al., 2022). Wu *et al.* developed an interaction index for fixed-dose, two-drug combination studies based on the bliss independence model (Wu et al., 2012). Huang *et al.* proposed a comprehensive statistical framework to evaluate interactions for TGI, both at a fixed time point and in given time ranges, exploiting the highest single agent, response additivity, and bliss independence methods (Huang et al., 2022). The application of classic methods for CI derivation in xenograft TGI studies is particularly suitable for screening exercises; however, the transfer of such *in vitro* concepts to *in vivo* models remains cumbersome. As such, it is questionable whether these methods can well describe effect saturation, whereby single treatments have already eradicated a significant portion of the tumor, and TGI as synergy readout is highly nonlinear with respect to its causation.

An alternative for determining CIs may be pharmacodynamic (PD) models, which include a semi-mechanistic study of tumor growth, where PD refers to tumour volume. Such studies are commonly used to describe the TGI evolution of single compounds in xenografts studies (Mayneord, 1932; Simeoni et al., 2004; Jumbe et al., 2010; Parra-Guillen et al., 2013; Evans et al., 2014; Ribba et al., 2014; Mistry et al., 2018; Orrell and Mistry, 2019). PD models have also been expanded to assess synergy (Rocchetti et al., 2009; Terranova et al., 2013; Tosca et al., 2021), they require individual modelling efforts for each tumour using numerical simulations, aggravating large screening exercises.

Therefore, we propose the radius additivity (Rad-add) score, a novel CI score for detecting PD interactions in fixed-dose xenograft TGI combination experiments, to study the pharmacodynamic synergy. The Rad-add score was derived from a semi-mechanistic tumor growth model (Mayneord, 1932; Jumbe et al., 2010; Evans et al., 2014; Mistry et al., 2018; Orrell and Mistry, 2019), but addressed the need to assess combination effects in xenograft studies with a simple index in a high-throughput fashion. We assessed its performance vis-à-vis the bliss and response additivity score using the Novartis dataset from (Gao et al., 2015), a large-scale study of patient-derived xenografts (PDX).

## 2 Results and discussion

**FIGURE 1:**
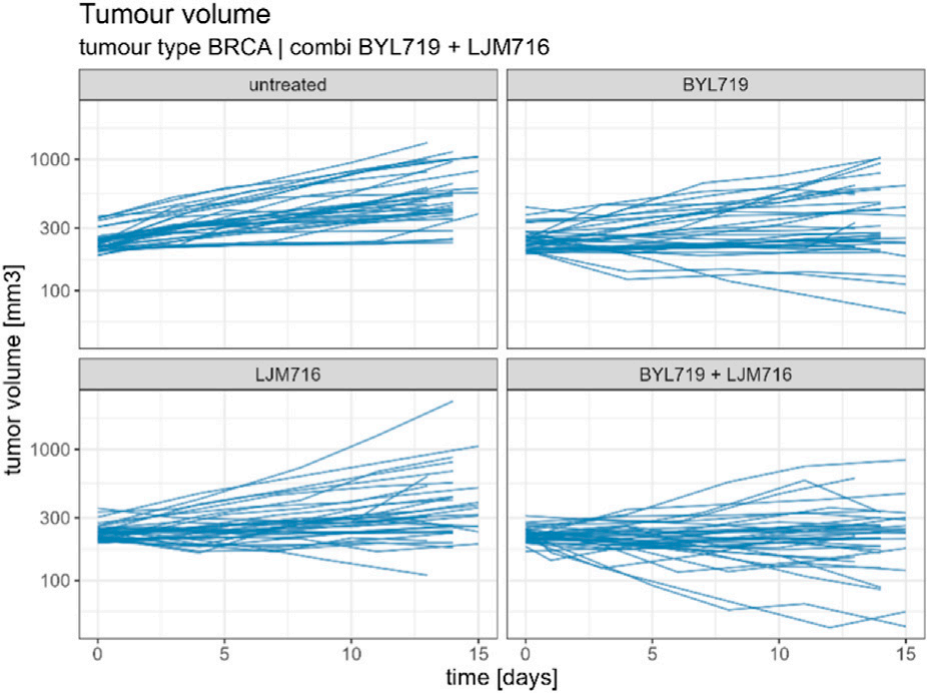
Example of combination study for BYL719 and LJM716 with BRCA as tumour type. The combination study is composed of one untreated arm, two single agent arms and one combination arm. Duration of the study was considered up to 14 ± 1 days.

From the Novartis PDX dataset, 22 combination studies (two single-agent arms, one combination arm, and one reference arm) for six different tumor types were derived from the Novartis PDX dataset. Data from the studies were resampled using a bootstrap algorithm to allow the statistical assessment of the combined indices. In Table 1 all tumor studies are listed, together with the targets of all considered compounds. An example of the combination study is shown in Figure 1.

**TABLE 1:** Selected combination studies form Novartis PDX dataset.

Tumour type	Drug A	Drug A target^a^	Drug B	Drug B target^a^
BRCA	BYL719	PI3K alpha	LEE011	CDK4/6
BRCA	BYL719	PI3K alpha	LJM716	HER3
BRCA	LJM716	HER3	trastuzumab	HER2
CM	BKM120	PI3K	encorafenib	BRAF
CM	encorafenib	BRAF	binimetinib	MEK1/2
CM	LEE011	CDK4/6	binimetinib	MEK1/2
CM	LEE011	CDK4/6	encorafenib	BRAF
CRC	BKM120	PI3K	LJC049	TNKS
CRC	BYL719	PI3K alpha	binimetinib	MEK1/2
CRC	BYL719	PI3K alpha	cetuximab	EGFR
CRC	BYL719	PI3K alpha	encorafenib	BRAF
CRC	cetuximab	EGFR	encorafenib	BRAF
GC	BYL719	PI3K alpha	HSP990	HSP90
GC	BYL719	PI3K alpha	LJM716	HER3
GC	INC280	MET	trastuzumab	HER2
GC	LEE011	CDK4/6	everolimus	mTOR
GC	LJM716	HER3	trastuzumab	HER2
NSCLC	BKM120	PI3K	binimetinib	MEK1/2
NSCLC	BYL719	PI3K alpha	LGH447	pan-PIM kinase
PDAC	BKM120	PI3K	binimetinib	MEK1/2
PDAC	figitumumab"	IGF-IR	binimetinib	MEK1/2
PDAC	INC424	JAK1/2	binimetinib	MEK1/2

^a^ target references: BYL719 (Juric et al., 2018); LJM716 (Reynolds et al., 2017); BKM120 (Rosenthal et al., 2020); encorafenib (Delord et al., 2017); LEE011 (Santoro et al., 2022); cetuximab (van Geel et al., 2017); figitumumab (Molife et al., 2010); INC280 (Moreno et al., 2021); INC424 (Passamonti et al., 2021); trastuzumab (Kong et al., 2016); binimetinib (Bardia et al., 2020); LJC049 (Smith and Sheltzer, 2018); HSP990 (Spreafico et al., 2015); everolimus (Tolcher et al., 2015); LGH447 (Raab et al., 2019).

**FIGURE 2:**
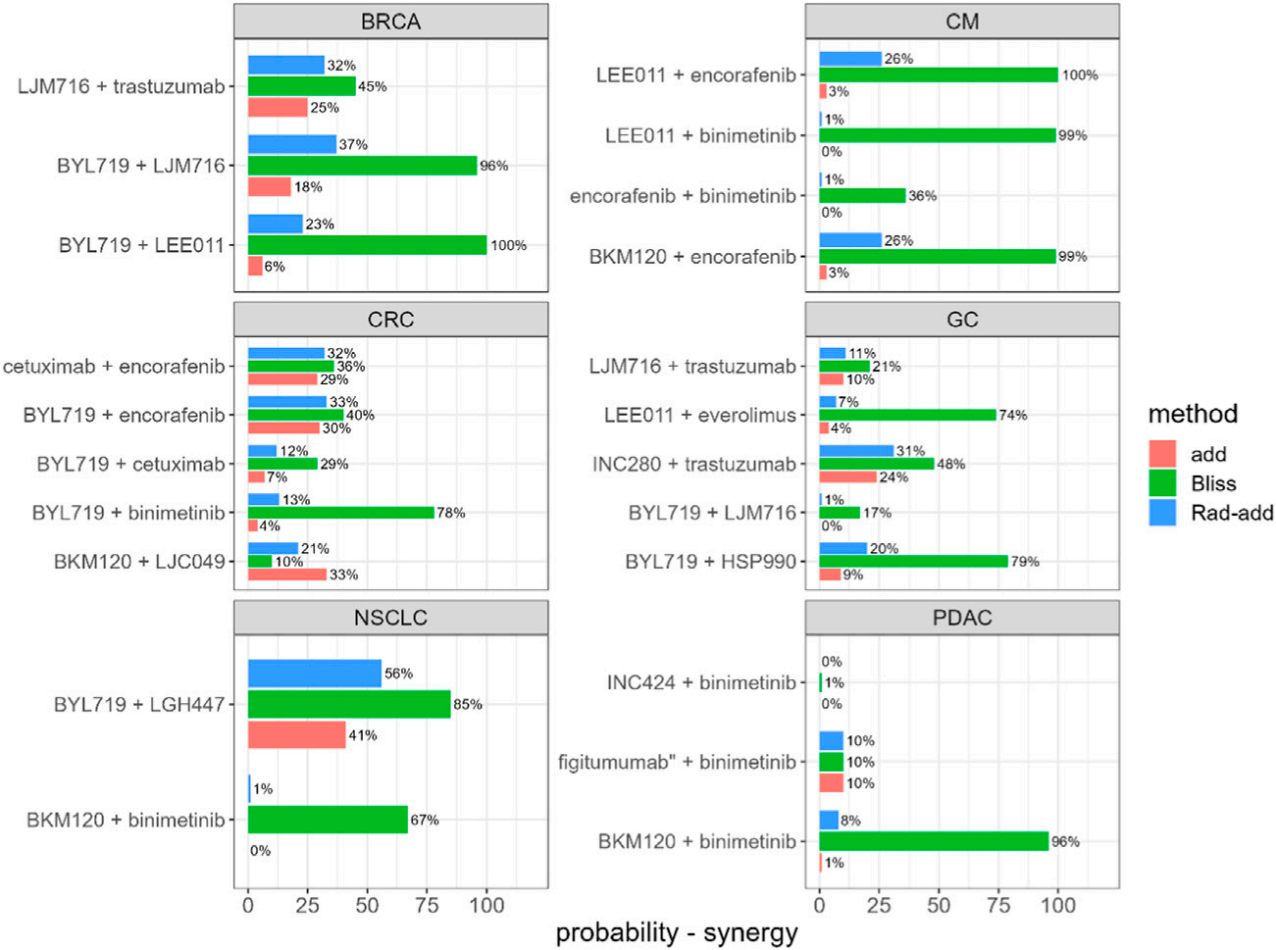
Predicted probability of synergistic interactions on the final dataset derived from the Novartis PDX database, stratified for tumour type and drug combination. *Rad-add* stands for radius additivity, *Bliss* for the Bliss independence method and *add* for the response additivity method.

Rad-add (see methods Section 3.2 for details), response additivity, and Bliss CIs were derived for all combination studies of the final dataset, and an uncertainty distribution was obtained using 1,000 bootstrap samples. For each combination study, the probability of the drug combination being classified as synergistic was derived as the percentage of bootstrap samples with CI<1. The probabilities of synergistic interactions for all considered combination studies with all investigated approaches are shown in Figure 2. In Supplementary Tables S1, S2, the calculated CIs with 90% confidence intervals are reported.

Figure 2 suggests that the Bliss method tends to predict synergy with a high probability for several combinations (e.g., for tumor-type BRCA, BYL719 + LJM716, and for tumor-type CM, LEE011 + encorafenib). Conversely, the response-additivity approach predicts a low probability of synergistic interaction. The highest predicted probability of synergy was approximately 40% for the study with NSCLC as the tumor type and the BYL719 + LGH447 combination. For most combination studies, the probabilities of being classified as synergistic with the Rad-add method seem to fall between the results of the response additivity and bliss approaches. As shown in Figure 2, only one combination (tumor-type NSCLC, BYL719 + LGH447) had a probability higher than 50% of being classified as synergistic with the Rad-add score. The overall low probability of synergistic classification with the Rad-add score is in accordance with previous reports, where drug synergy was often overstated in both clinical and PDX trials (Ocana et al., 2012; Palmer and Sorger, 2017; Plana et al., 2022).

As shown in Supplementary Figure S1, the probabilities for a combination classed as synergistic with the Rad-add approach correlate well (*ρ* = 0.82, *p* <0.01) with those calculated via the response additivity method, with the former generally being higher than the latter. This is probably because both the methods rely on the assumption of additivity. Conversely, probabilities calculated using the Rad-add score were not significantly correlated with those calculated using the Bliss approach (ρ = 0.28, *p* = 0.2), where the former are generally lower than the latter.

The Rad-add score, which is based on observed values at the beginning and the end of the experiment approximates a model-based analysis, conducted via regressing a model, Eq 6 with *f(cmp) = 1*, against the time-series data, which also assumes that the tumor radius grows linearly over the time interval of interest. The similarity between the TGI derived using the Rad-add score method and those derived from regressing a model against the time-series was also assessed. In Supplementary Figure S2, the predicted TGI using the empirical Rad-add method is compared with those derived from regressing the underlying PD model against the time-series data. The two metrics correlate well (*ρ* = 0.98, *p* <0.01), suggesting that the radius additivity method is a good approximation of the model-based analysis via regression.

In this study, a novel CI method, the Rad-add score, was developed for fixed-dose, drug-combination, and xenograft TGI studies. The Rad-add score is derived from the mechanistic understanding of tumor volume growth and is intended as an approximation of a model-based analysis performed using the constant radius growth model. The Rad-add score incorporates the advantages of model-based analysis, such as the description of synergy in a semi-mechanistic framework of disease progression and drug actions, together with the speed of CI calculation. The latter characteristic makes the Rad-add score suitable for screening. Indeed, the Rad-add score has some drawbacks. Like other CI approaches, the Rad-add score is currently developed only by using PD profiles, ignoring pharmacokinetics, and therefore potential effects on tumor growth due to drug-drug interactions. Finally, the Rad-add score is only suitable for fixed-dose xenograft combination TGI studies, whereas other CI methods can be used in diverse contexts.

In conclusion, in the context of xenograft combination TGI studies, we believe that the Rad-add score is a suitable method for detecting synergy, as it combines the advantages of model-based analysis with those of CI-based methods.

## 3 Methods

### 3.1 TGI and CI with response additivity and Bliss models

In a standard xenograft TGI combination study, one reference arm, *k* single-agent arms (one for each treatment), and one combination arm were typically used. In such studies, TGI was considered an indicator of treatment efficacy. For a given treatment τ , $TGI_{\tau }$ can be defined as follows:

$$TGI_{\tau }=\left(1-\frac{V_{\tau }\left(t_{end}\right)-V_{\tau ,0}}{V_{r}\left(t_{end}\right)-V_{r,0}}\right)\cdot 100$$ (1)

Where *V(t*_*end*_ *)* and *V*_*0*_ are the tumor volumes at the end and beginning of the experiment, respectively. Subscript *r* refers to the reference arm The CI for TGI is calculated as follows, where $TGI_{pred,add}$ is the predicted TGI in the case of additivity and $TGI_{combi}$ is the TGI of the combination arm.

$$CI=\frac{TGI_{pred,add}}{TGI_{combi}}$$ (2)

CI equal to, lower than, and higher than 1 represent additivity, synergy, and antagonism, respectively. The CI depends on the method used to calculate $TGI_{pred,add}$ . For the case of two treatments (A and B) administered in combination, $TGI_{pred,add}$ calculated according to the response additivity and Bliss model ( $TGI_{add}$ and $TGI_{Bliss}$ ) are as follows:

$$\begin{array}{c} TGI_{add}=TGI_{A}+TGI_{B}\\ TGI_{Bliss}=TGI_{A}+TGI_{B}-TGI_{A}\cdot TGI_{B} \end{array}$$ (3)

The hypothesis that TGI_add_ relies on is a linear dose-effect relationship, whereas TGI_Bliss_ is an exponential dose-effect relationship (Foucquier and Guedj, 2015). TGI_Bliss_ is based on the principle of statistical independence. Therefore, to calculate TGI_Bliss_, TGI_A_ and TGI_B_ must be treated as probabilities (between zero and one). In xenograft experiments, it is not uncommon to observe a TGI effect higher than 100%; in such cases, the requirement of the TGI to calculate the Bliss score is not respected.

### 3.2 Derivation of the radius additivity (Rad-add) score

In 1932, Mayneord showed that the increase in the long diameter of tumors implanted in rats followed a linear law. This was explained by the fact that not the whole tumor mass is in a state of active growth, but only a thin capsule enclosing a necrotic core (Mayneord, 1932). This observation forms the basis of numerous TGI models used to analyze preclinical drug development data (Jumbe et al., 2010; Evans et al., 2014; Mistry et al., 2018; Orrell and Mistry, 2019). From such observations, if we map the observed tumour volume onto a spherical shape, the following model for radius growth in the untreated case can be derived:

$$\frac{dR}{dt}=g$$ (4)

Where *R* is the radius of the tumor and *g* is the radius growth rate constant. When treatment begins, the reference growth rate may be decreased by the pharmacological action of the compounds, which depends on their exposure (*c(t)*) and potency (*IC*_*50*_). Typically, such pharmacological actions follow Hill’s function.

$$f\left(cmp\right)=\frac{c{\left(t\right)}^{h}}{c{\left(t\right)}^{h}+IC_{50}^{h}}$$ (5)

where *h* (Hill coefficient) and *IC*_*50*_ can be obtained from *in vitro* experiments or estimated directly from *in vivo* experiments, respectively. *f(cmp)* can be used to describe tumor growth under treatment as follows:

$$\frac{dR}{dt}=g-d\cdot f\left(cmp\right)$$ (6)

where *d* is the decay parameter representing the maximal decay rate caused by a certain treatment for a certain tumor and mouse strain. From Eq 6, it is possible to easily derive the analytic expression of *R(t)*, where *R*_*0*_ is the radius at the beginning of the experiment (t = 0).

$$R\left(t\right)=R_{0}+g\cdot t-d\cdot \int_{0}^{t}f\left(cmp\right)\;dt$$ (7)

For simplicity, we define $\psi_{i}=d\cdot \int_{0}^{t}f\left(cmp_{i}\right)\;dt$ as the effect on the radius growth at time *t* of a given treatment *cmp*_*i*_ and $G_{t}=g\cdot t$ as the unperturbed tumor growth at time *t*. The additive effect of a combination of treatments, *i = 1…k*, is defined as follows:

$$R_{RA}\left(t\right)=R_{0}+G_{t}-\sum_{i}\psi_{i}$$ (8)

By determining the radius at the end of the experiment (*t*_*end*_) and *R*_*0*_ for the reference arm, it is trivial to calculate *G*_*t*_. Once *G*_*t*_ is known, $\psi_{i}$ can be derived for all considered compounds (if the radii at the beginning and end of the experiments are known for each treatment). (Thus, Rad-add is an empirical metric, which attempts to approximate a model-based metric which would involve regressing Eq 6 against the time-series data.) Once *G*_*t*_ and $\psi_{i}$ , *i = 1…k*, are known, $R_{RA}\left(t_{end}\right)$ can be predicted using Eq 8, where R0 is equal to the radius at the beginning of the experiment for the combination arm. From $R_{RA}\left(t_{end}\right)$ the predicted volume at the end of the experiments under the hypothesis of additivity can then be derived using V = 4/3 π R ^ 3, i.e., mapping back to the spherical volume, and the TGI can be calculated according to Eq 1. If TGI_combi_ is known, CI can be calculated using Eq 2.

Notably, the Rad-add score is agnostic in terms of the shapes of both control radius growth and drug-mediated effects, as *G*_*t*_ and $\psi_{i}$ are directly calculated from the tumor growth curves. Therefore Rad-add can be considered an empirical value, as stated above. A model-based version of the score can also be calculated, termed PD model, by regressing Eq 6 against the time-series data. Within, this study only single doses of the compound were available and so *f(cmp)* = 1, i.e., no *in-vitro* data were used and an IC50 value from the *in-vivo* data cannot be estimated.

### 3.3 Comparison of Rad-add score with response additivity and Bliss

The performance of Rad-add, response additivity, and Bliss scores were compared on the public PDX dataset by Novartis (Gao et al., 2015). In such datasets, different tumor growth curves can be obtained for different patients, tumor types, and treatments. The latter can be either a single agent or combination of agents. Different PDX experiments were performed for the same tumor types and treatments. These PDXs are characterized by different mutations, copy number alterations, and mRNA expression levels.

Combination studies were performed to calculate CIs. Only combinations of two compounds were considered. Each combination study was characterized by a combination arm, two single-agent arms (one for each treatment), and a control arm. The duration of the experiments was from the start of treatment (day 0) to day 14 ± 1. Dataset preprocessing consisted of the following steps:

• For each tumor type, only treatments that were part of a combination and dedicated single-arm study were selected.
• Time series with measurements from days 0–14 ± 1 were selected. Time series that did not reach Day 13 were excluded.
• Arms with only one subject were discarded.

For all combination studies in the final dataset, the CI using Rad-add, response additivity, and Bliss methods were calculated. The CI distributions were obtained by bootstrapping with 1,000 samples. For each CI, the probability of being classified as synergistic was calculated as the proportion of bootstrap samples, where a CI<1. For the Rad-add CI calculation, tumor volumes in the Novartis PDX dataset were converted to radii with the hypothesis that tumors are spherical in shape.

The analysis was performed using the R software (version 4.2.1 (R Core Team, 2022). The codes and information for reproducibility are available in the Supplementary Material.

## Data Availability

The original contributions presented in the study are included in the article/Supplementary Material, further inquiries can be directed to the corresponding author.
