# Supplementary figures and images for: Radius additivity score: a novel combination index for tumour growth inhibition in fixed-dose xenograft studies
Source: Front Pharmacol. 2023 Oct 13;14:1272058. doi: 10.3389/fphar.2023.1272058 (PMC10603293; doi:10.3389/fphar.2023.1272058)

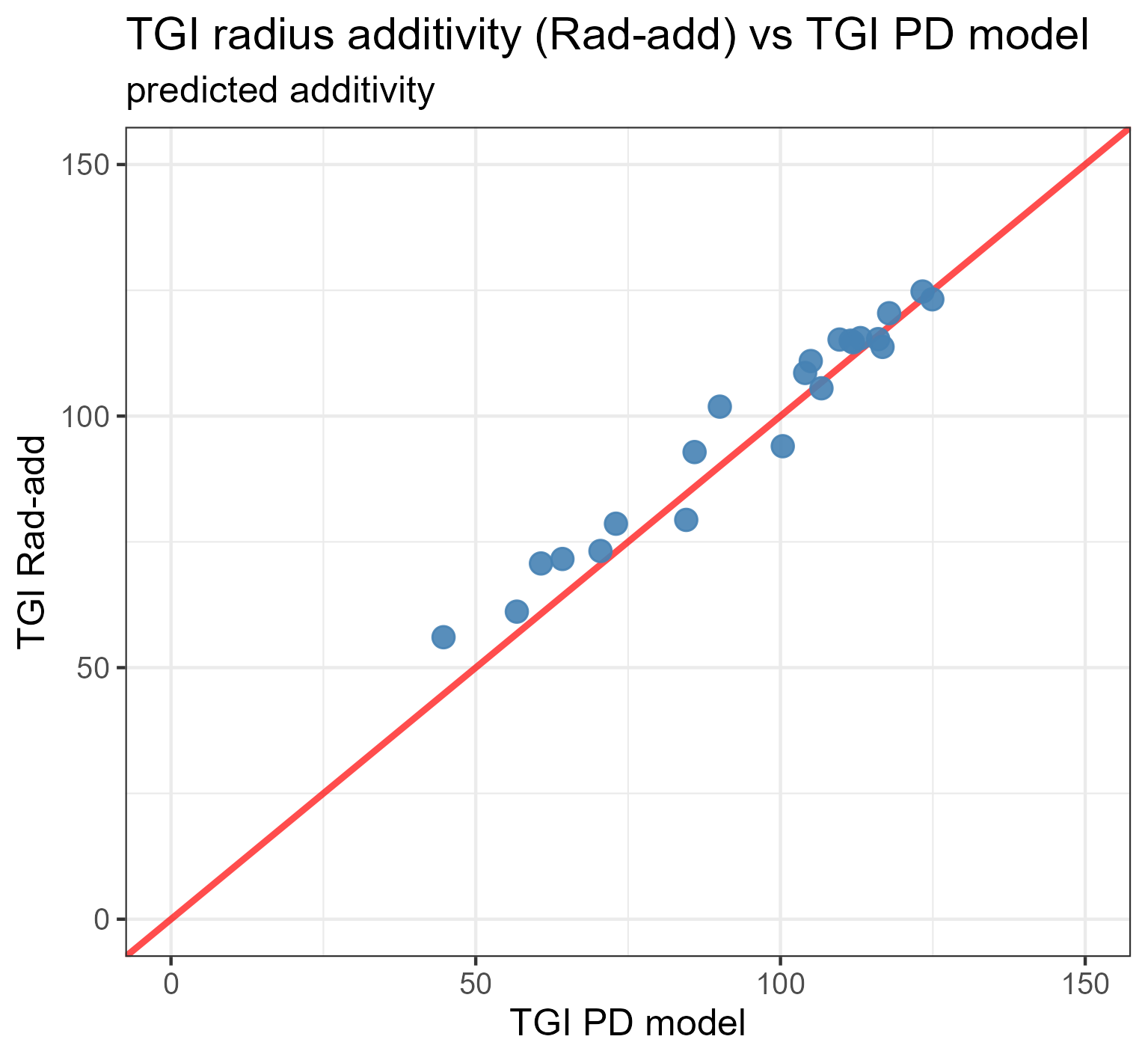

Supplement: Supplementary file 1 [file DataSheet1.ZIP › code_complete/results_comp_RA_vs_PD_model/TGI_RA_vs_TGI_PD_model.png]

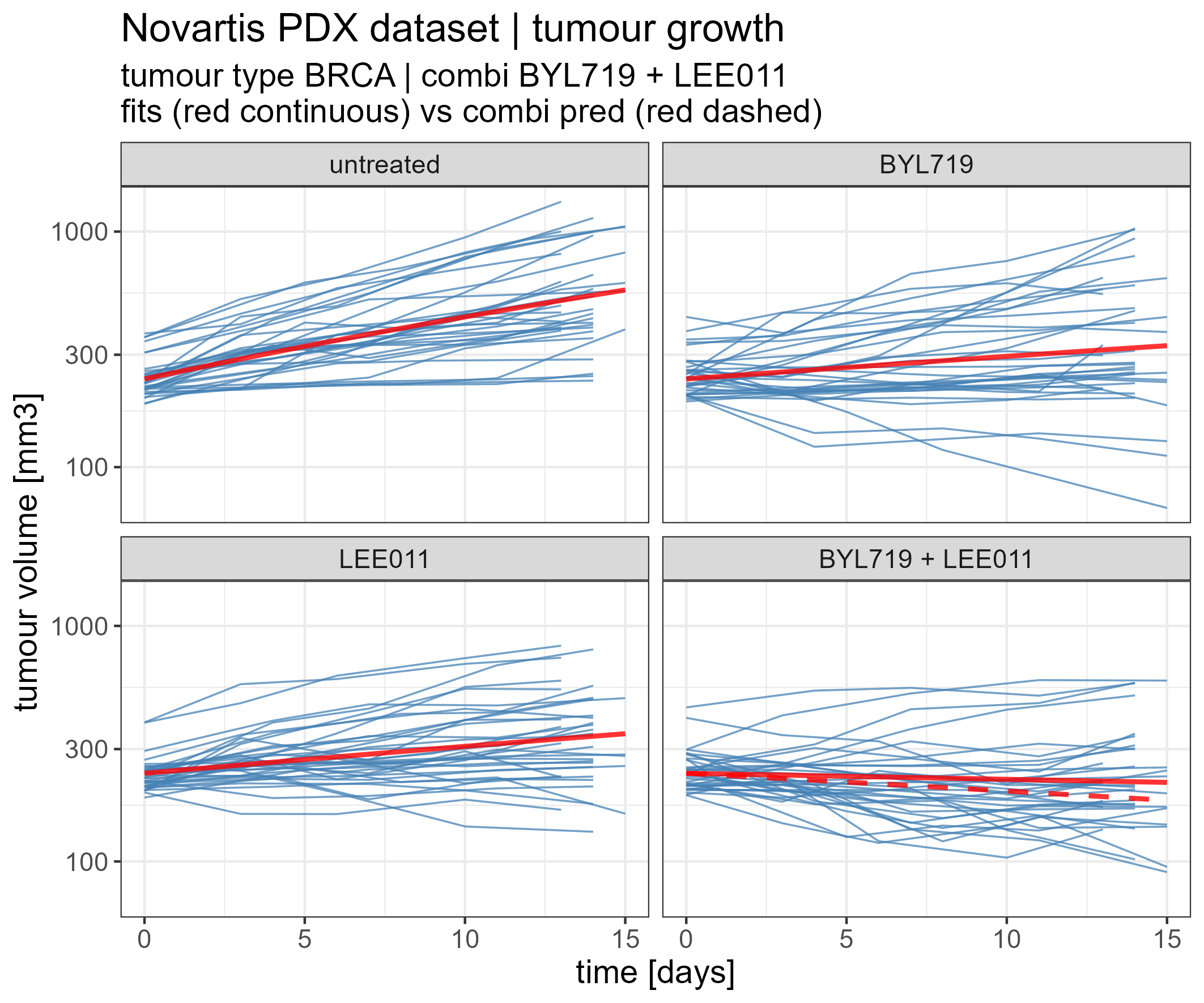

Supplement: Supplementary file 1 [file DataSheet1.ZIP › code_complete/results_PD_models_synergy_2/BRCA_BYL719_LEE011.png]

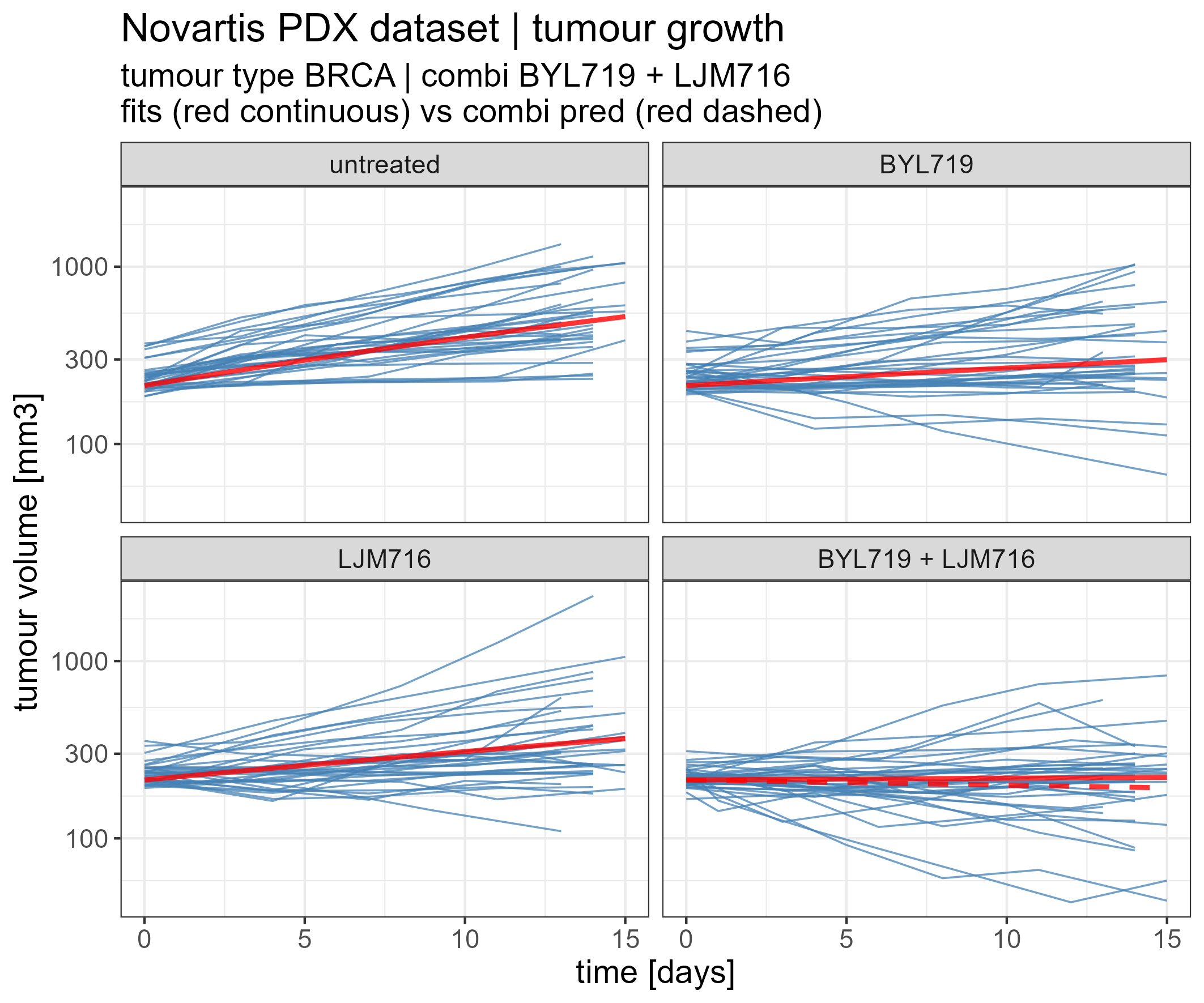

Supplement: Supplementary file 1 [file DataSheet1.ZIP › code_complete/results_PD_models_synergy_2/BRCA_BYL719_LJM716.png]

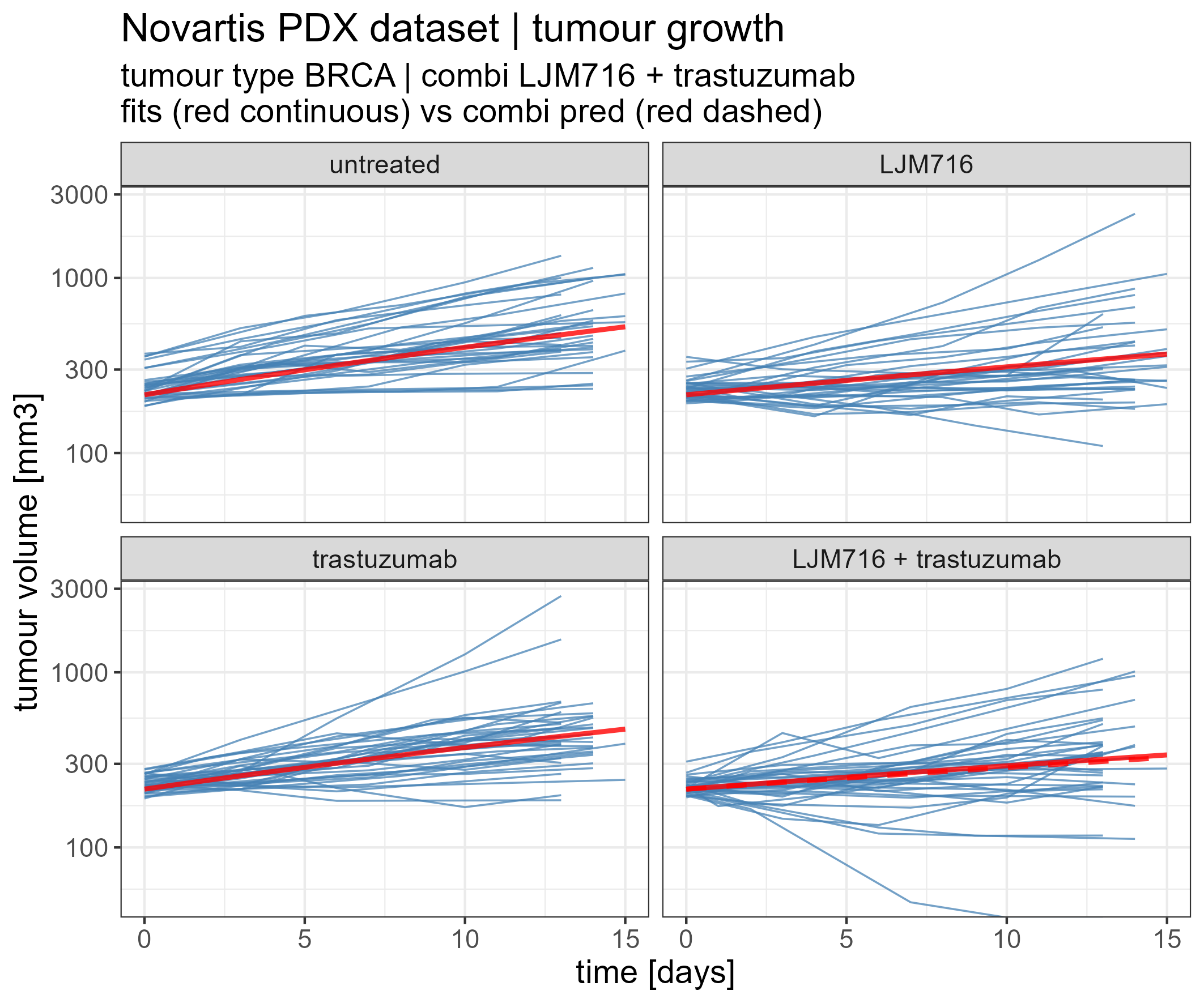

Supplement: Supplementary file 1 [file DataSheet1.ZIP › code_complete/results_PD_models_synergy_2/BRCA_LJM716_trastuzumab.png]

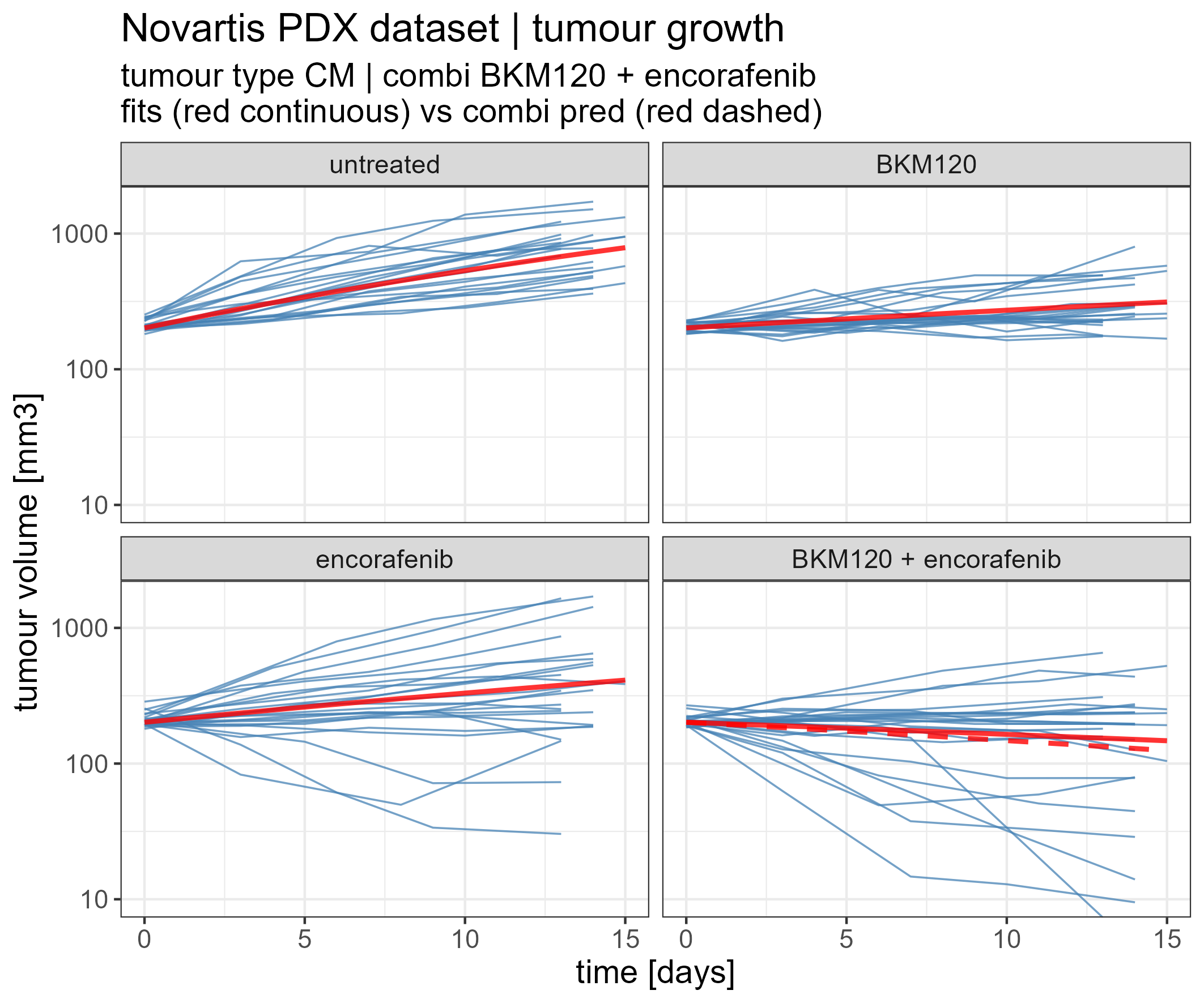

Supplement: Supplementary file 1 [file DataSheet1.ZIP › code_complete/results_PD_models_synergy_2/CM_BKM120_encorafenib.png]

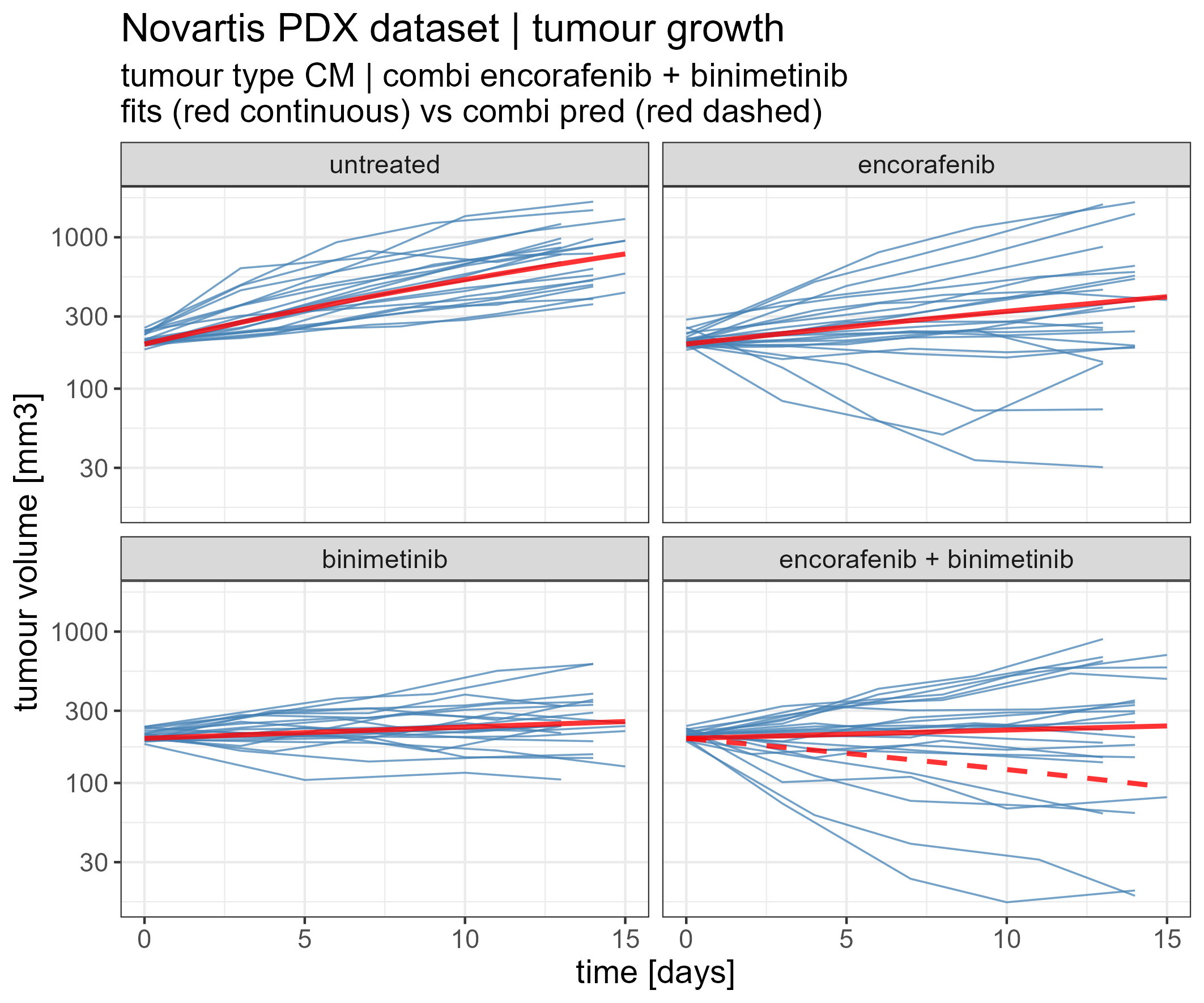

Supplement: Supplementary file 1 [file DataSheet1.ZIP › code_complete/results_PD_models_synergy_2/CM_encorafenib_binimetinib.png]

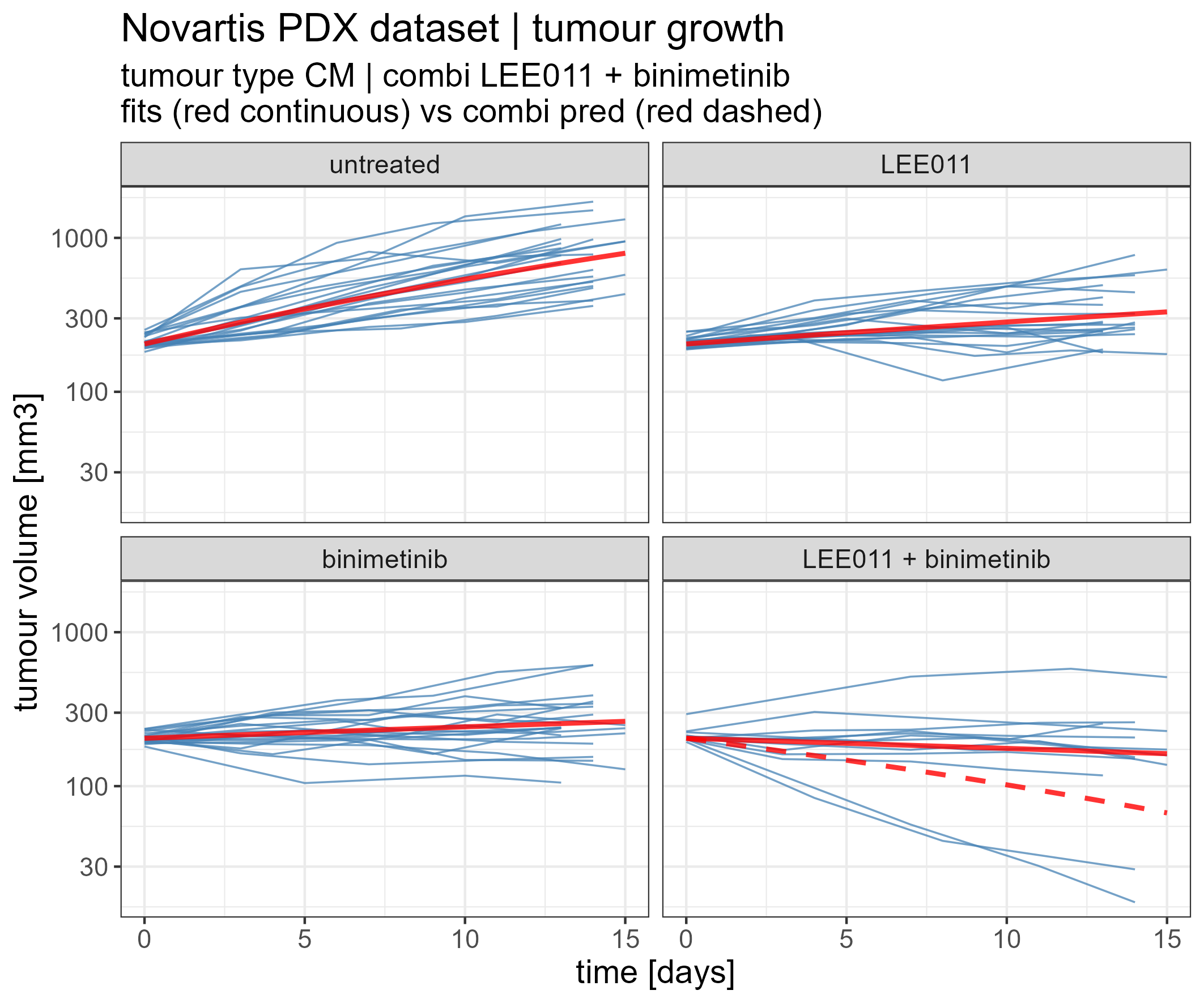

Supplement: Supplementary file 1 [file DataSheet1.ZIP › code_complete/results_PD_models_synergy_2/CM_LEE011_binimetinib.png]

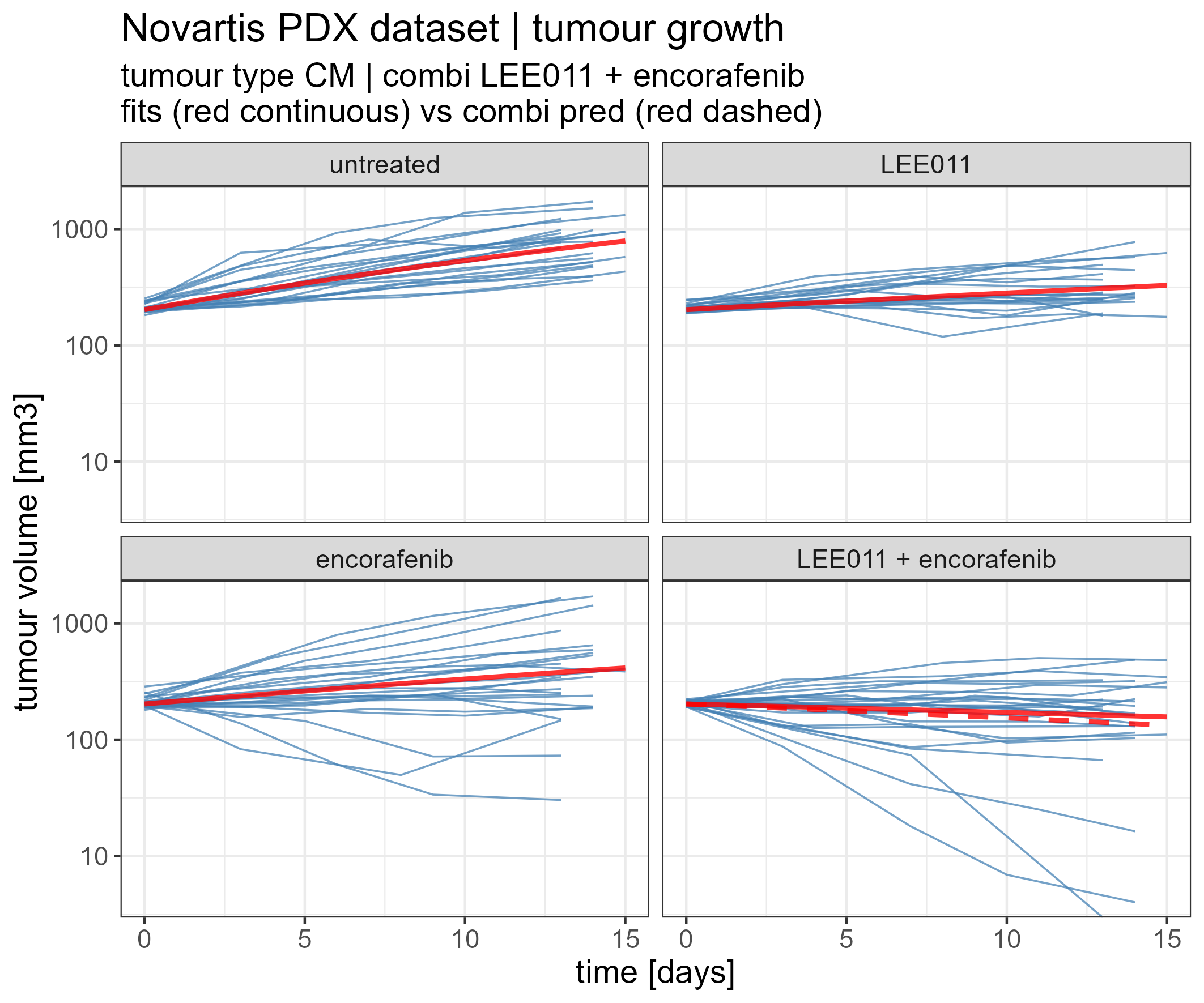

Supplement: Supplementary file 1 [file DataSheet1.ZIP › code_complete/results_PD_models_synergy_2/CM_LEE011_encorafenib.png]

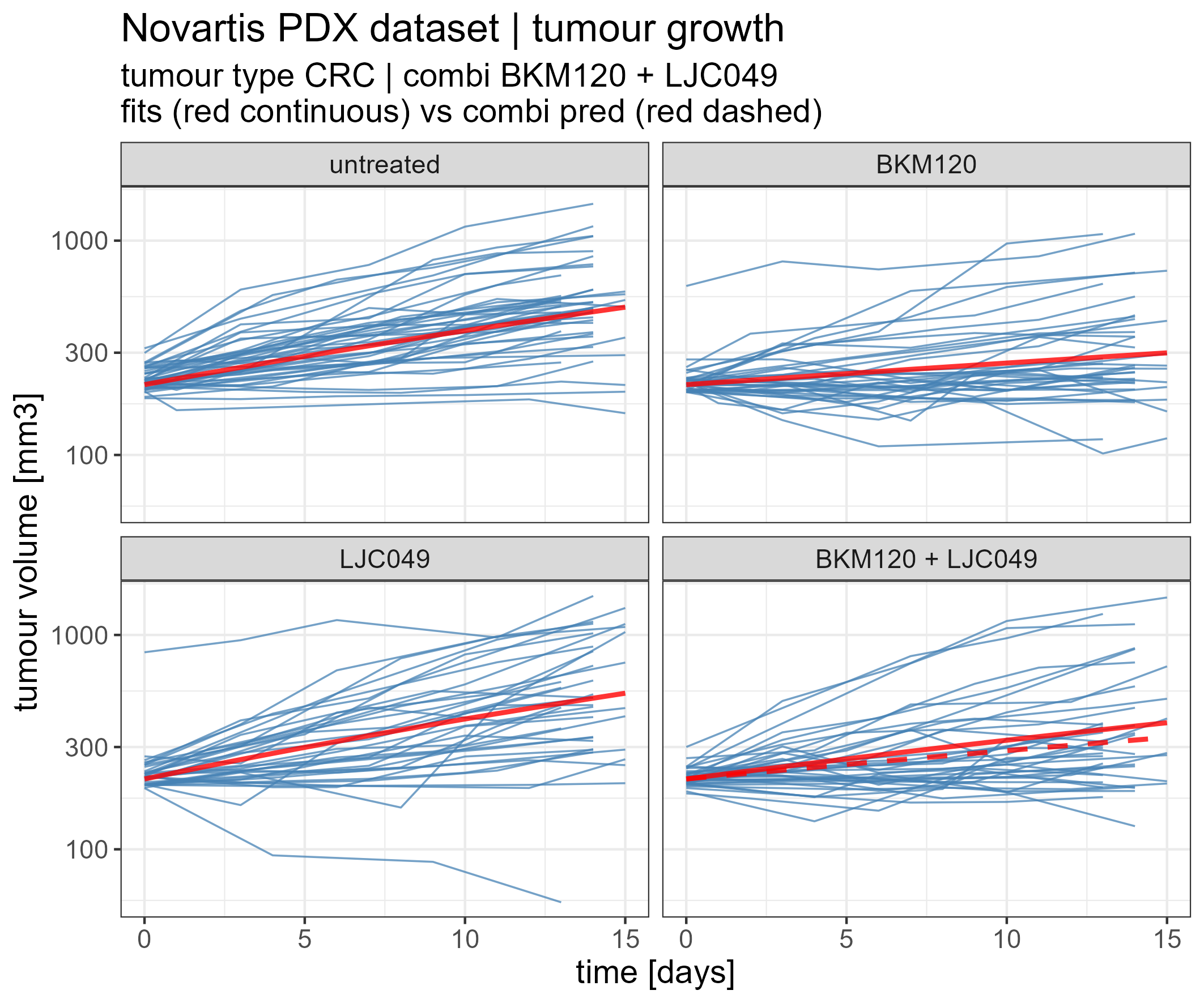

Supplement: Supplementary file 1 [file DataSheet1.ZIP › code_complete/results_PD_models_synergy_2/CRC_BKM120_LJC049.png]

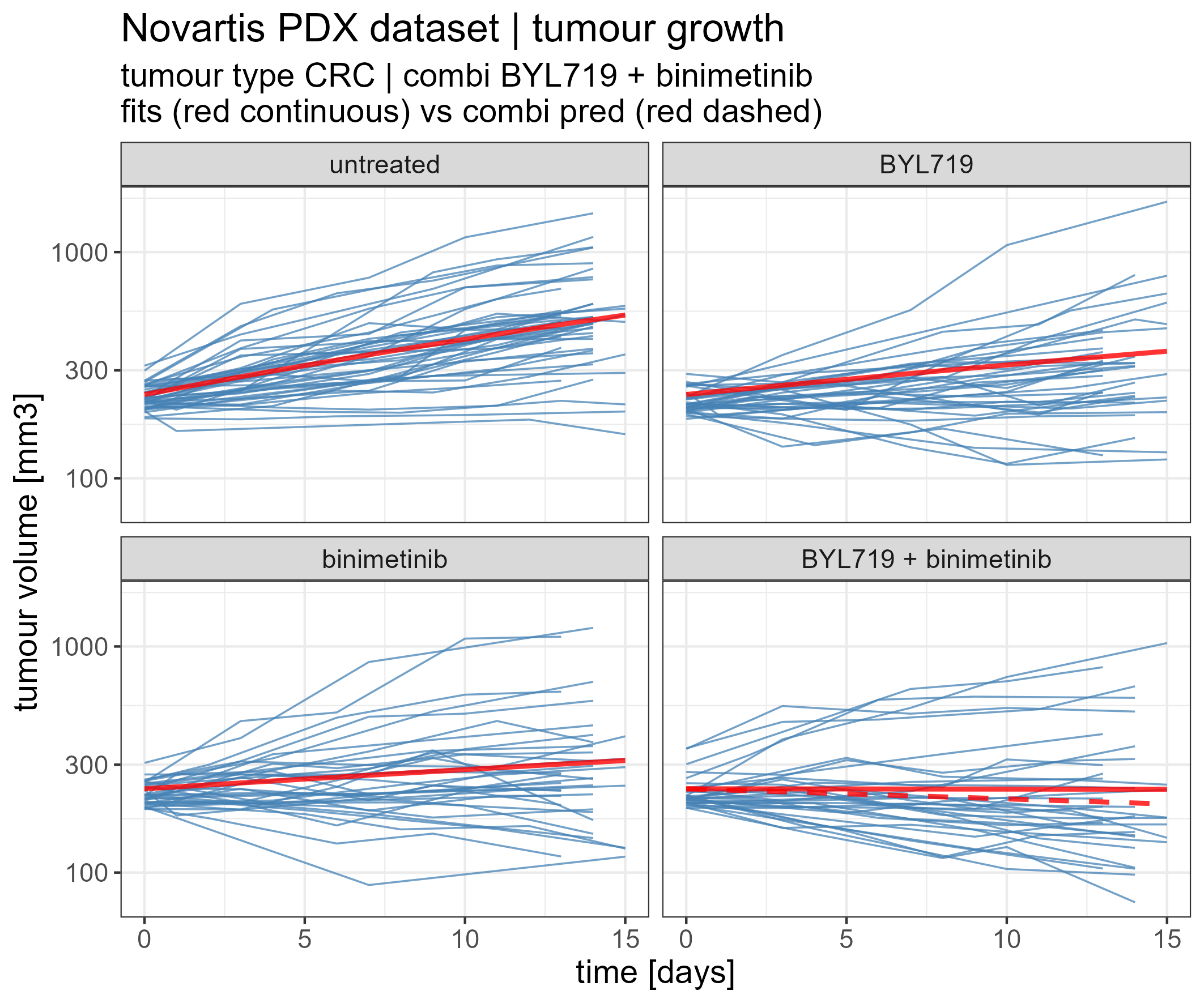

Supplement: Supplementary file 1 [file DataSheet1.ZIP › code_complete/results_PD_models_synergy_2/CRC_BYL719_binimetinib.png]

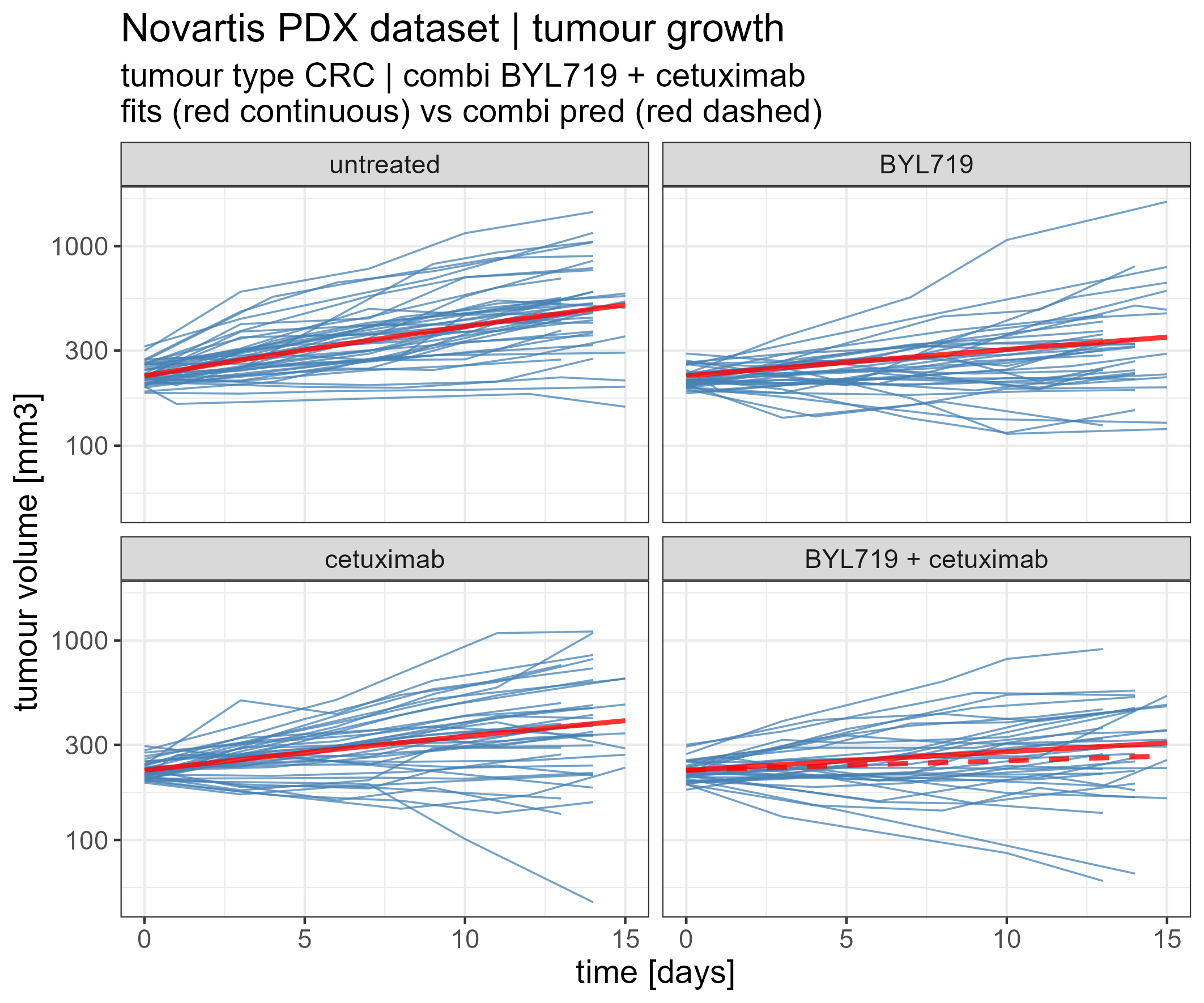

Supplement: Supplementary file 1 [file DataSheet1.ZIP › code_complete/results_PD_models_synergy_2/CRC_BYL719_cetuximab.png]

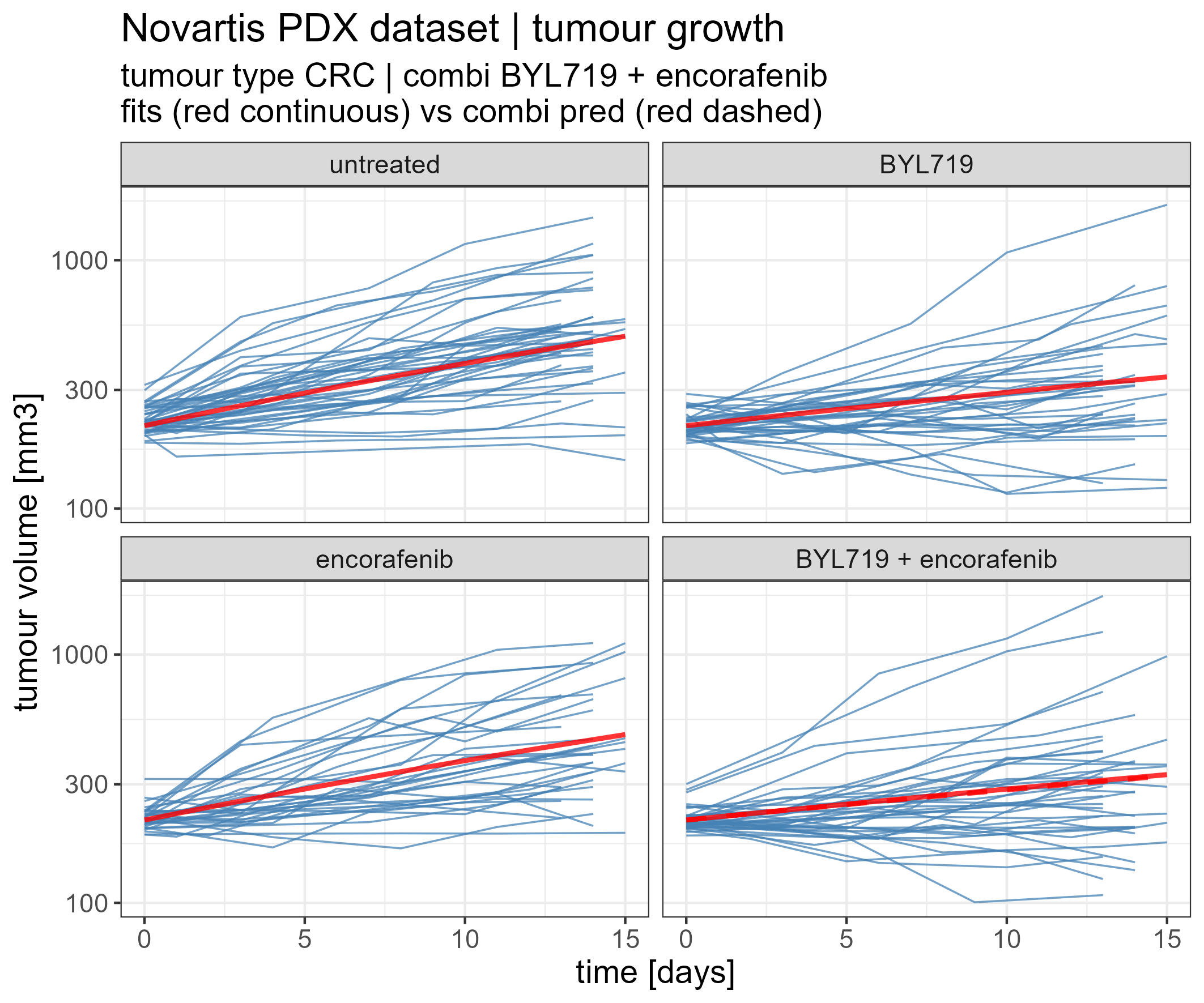

Supplement: Supplementary file 1 [file DataSheet1.ZIP › code_complete/results_PD_models_synergy_2/CRC_BYL719_encorafenib.png]

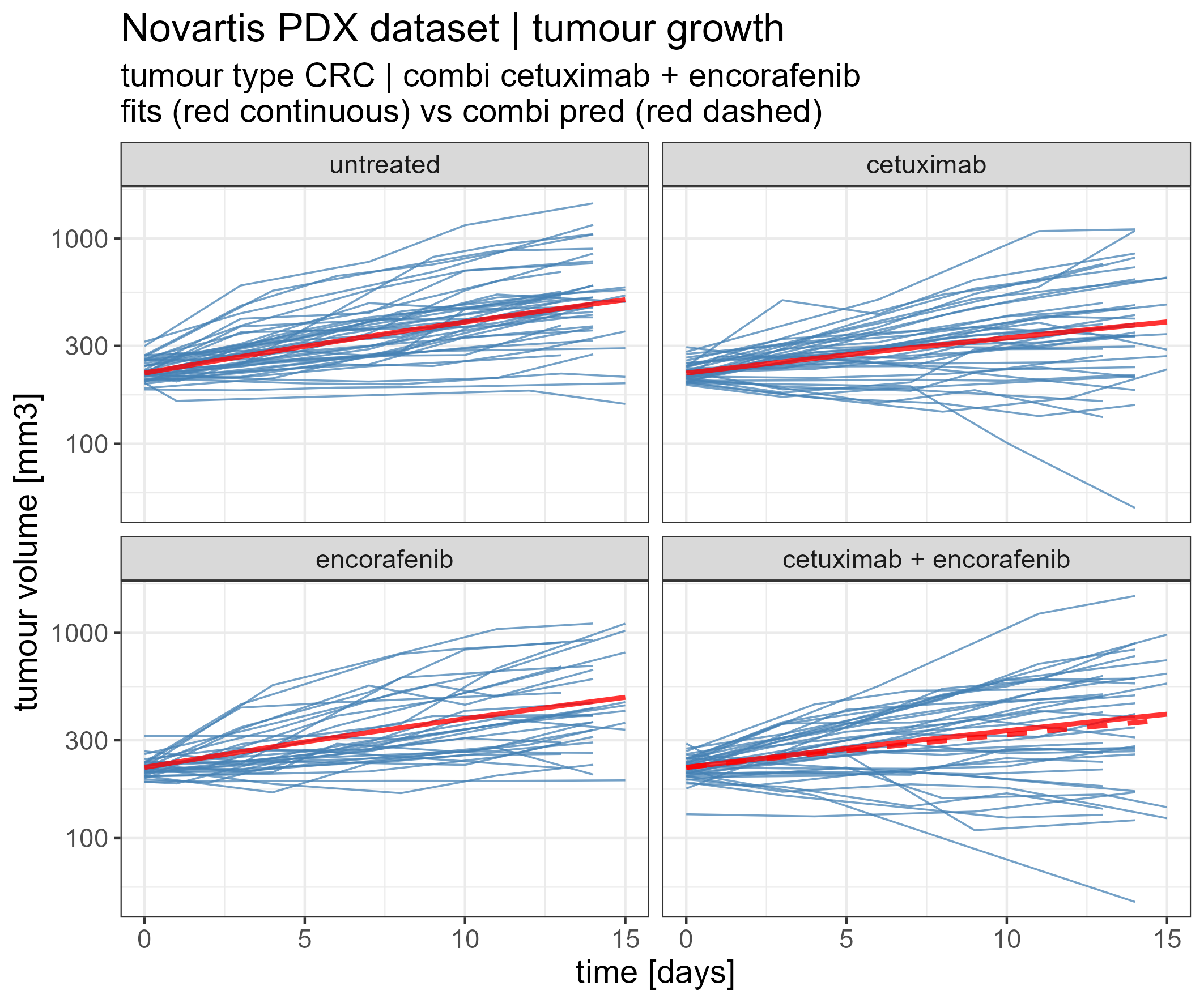

Supplement: Supplementary file 1 [file DataSheet1.ZIP › code_complete/results_PD_models_synergy_2/CRC_cetuximab_encorafenib.png]

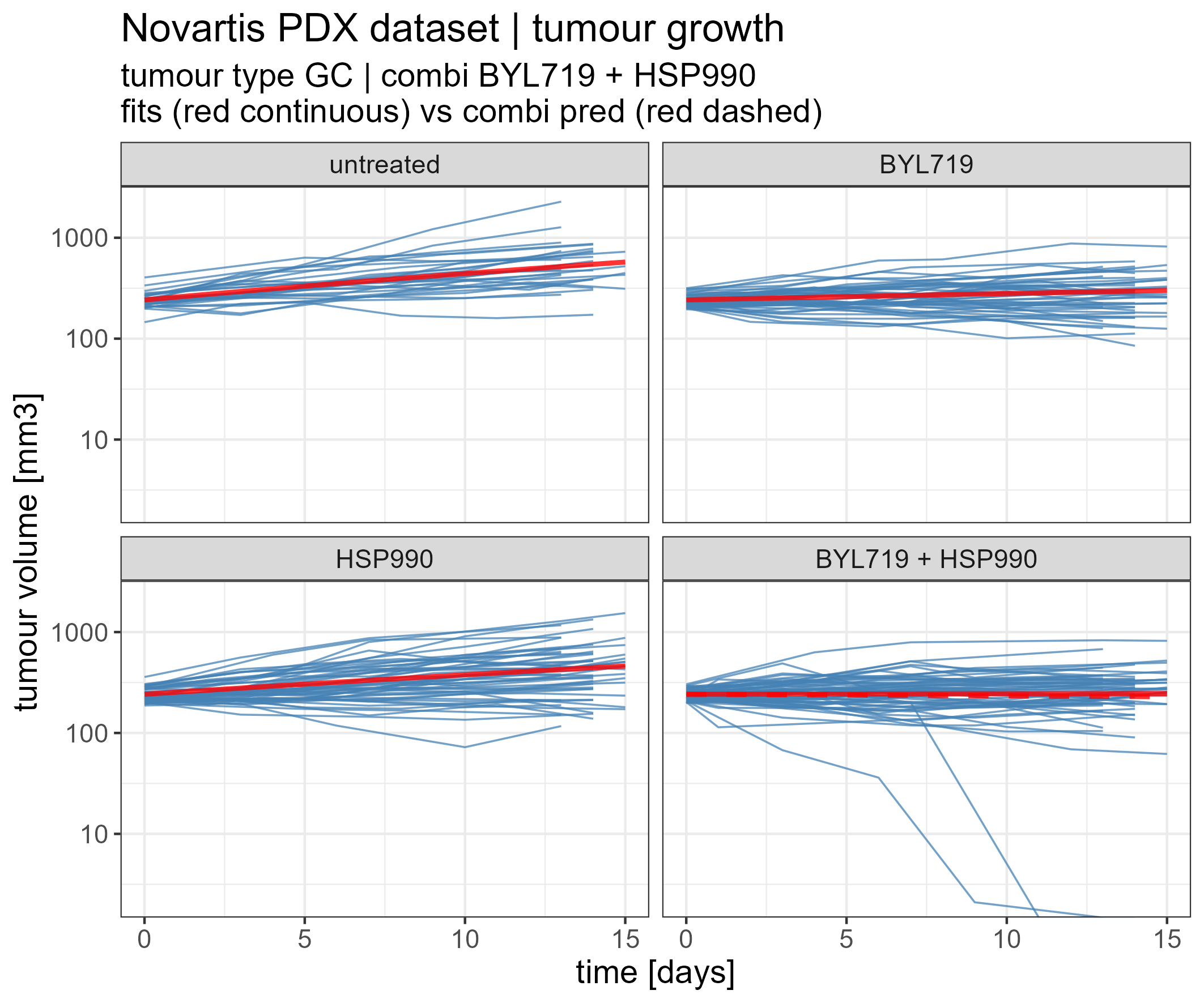

Supplement: Supplementary file 1 [file DataSheet1.ZIP › code_complete/results_PD_models_synergy_2/GC_BYL719_HSP990.png]

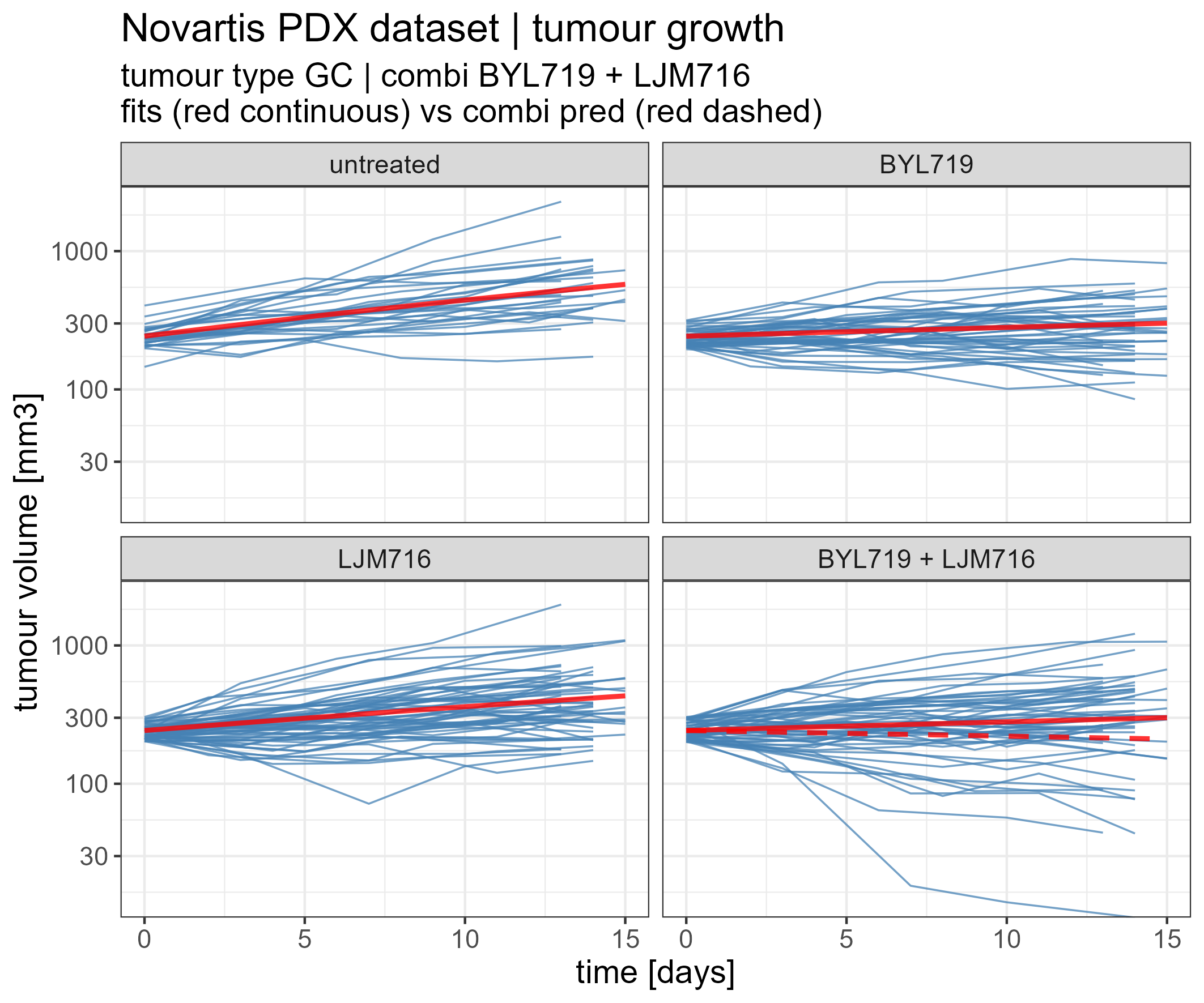

Supplement: Supplementary file 1 [file DataSheet1.ZIP › code_complete/results_PD_models_synergy_2/GC_BYL719_LJM716.png]

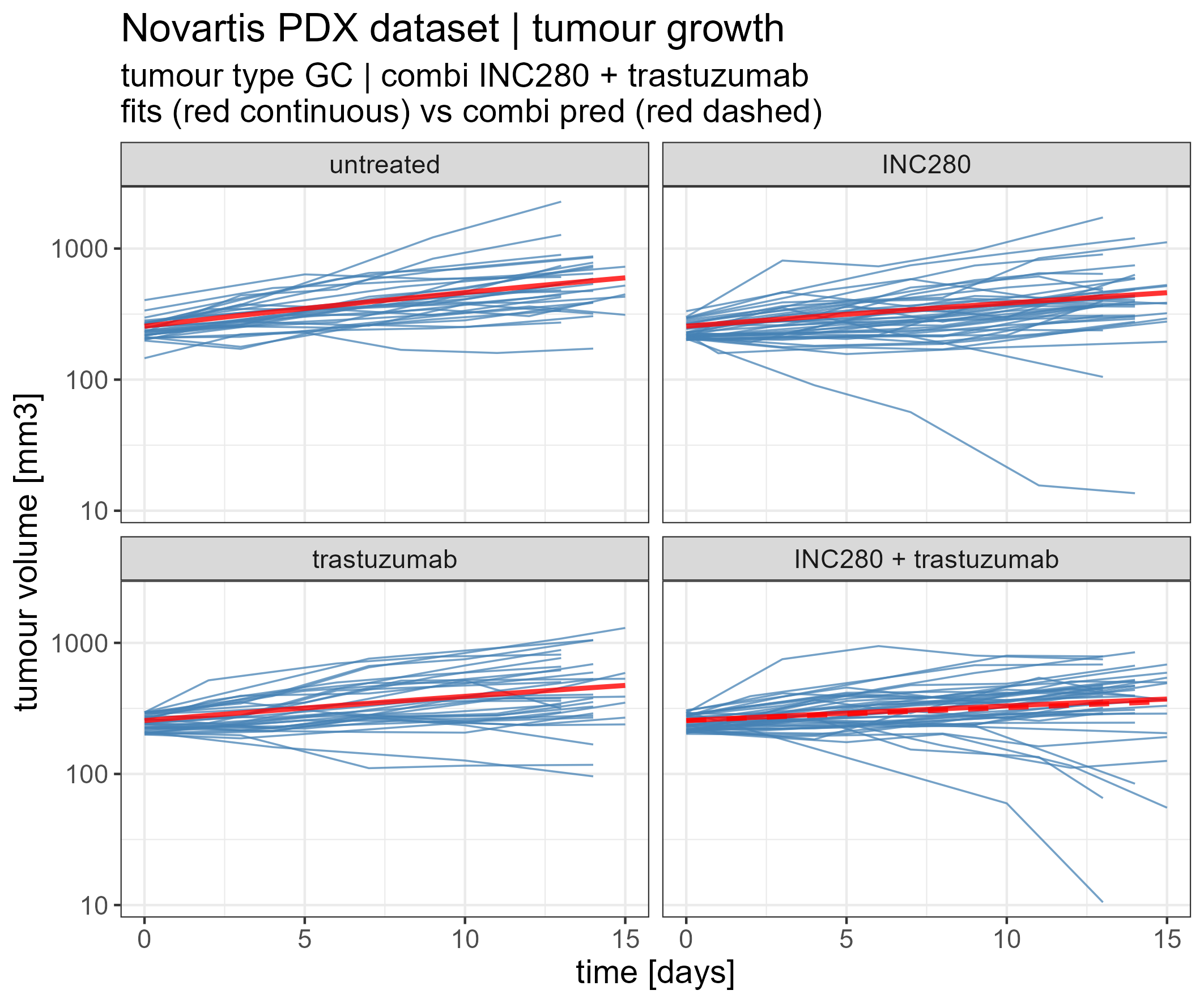

Supplement: Supplementary file 1 [file DataSheet1.ZIP › code_complete/results_PD_models_synergy_2/GC_INC280_trastuzumab.png]

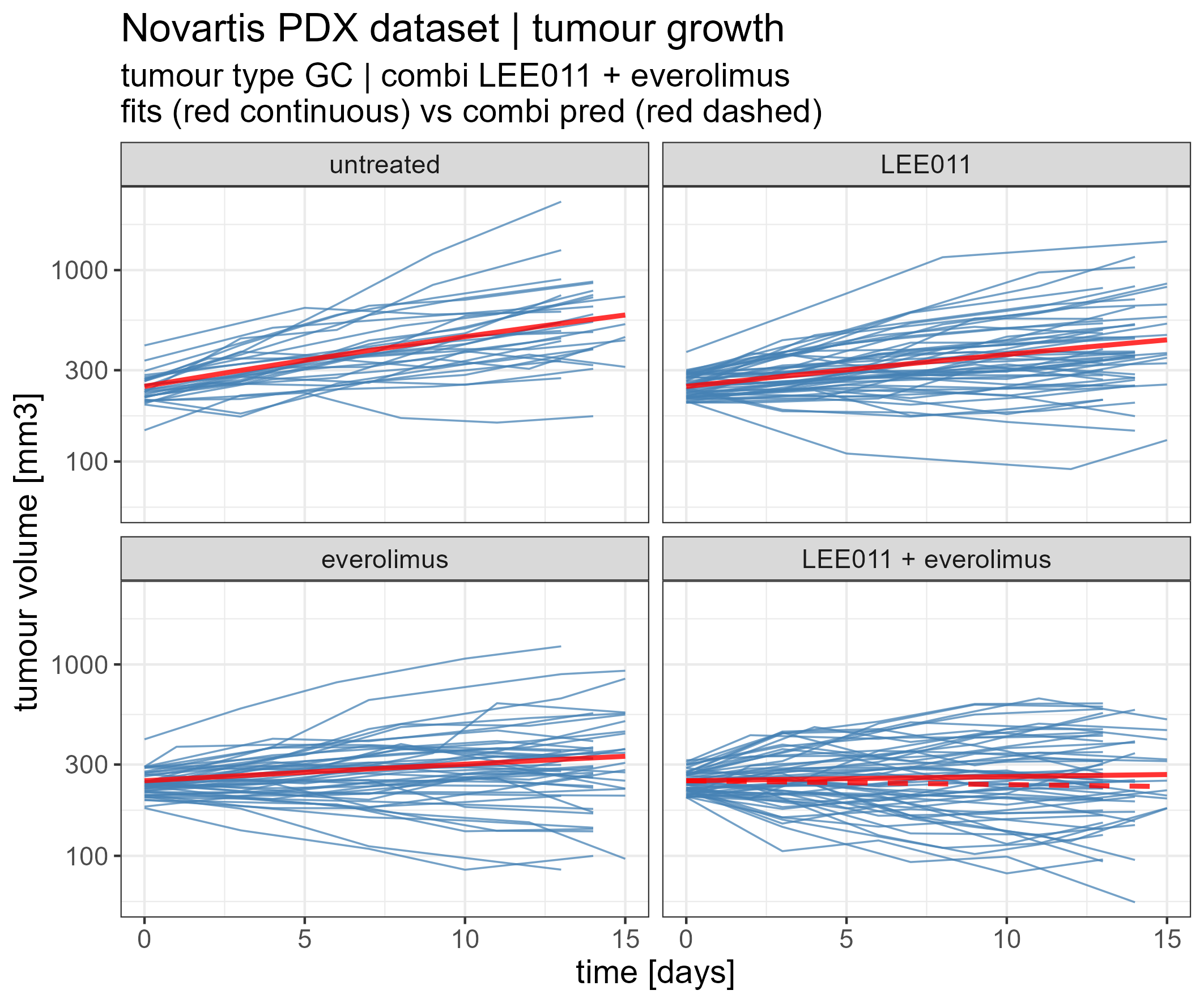

Supplement: Supplementary file 1 [file DataSheet1.ZIP › code_complete/results_PD_models_synergy_2/GC_LEE011_everolimus.png]

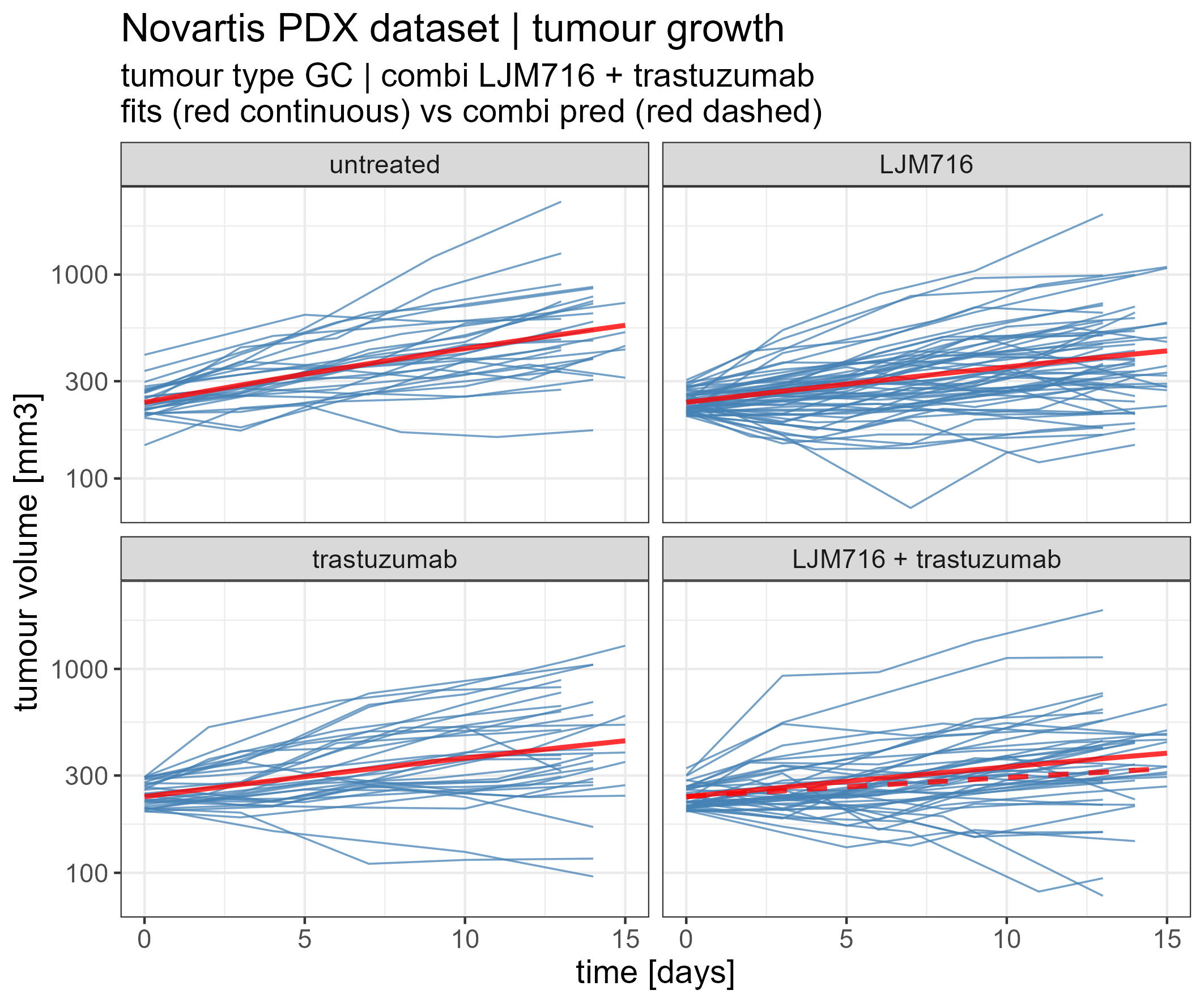

Supplement: Supplementary file 1 [file DataSheet1.ZIP › code_complete/results_PD_models_synergy_2/GC_LJM716_trastuzumab.png]

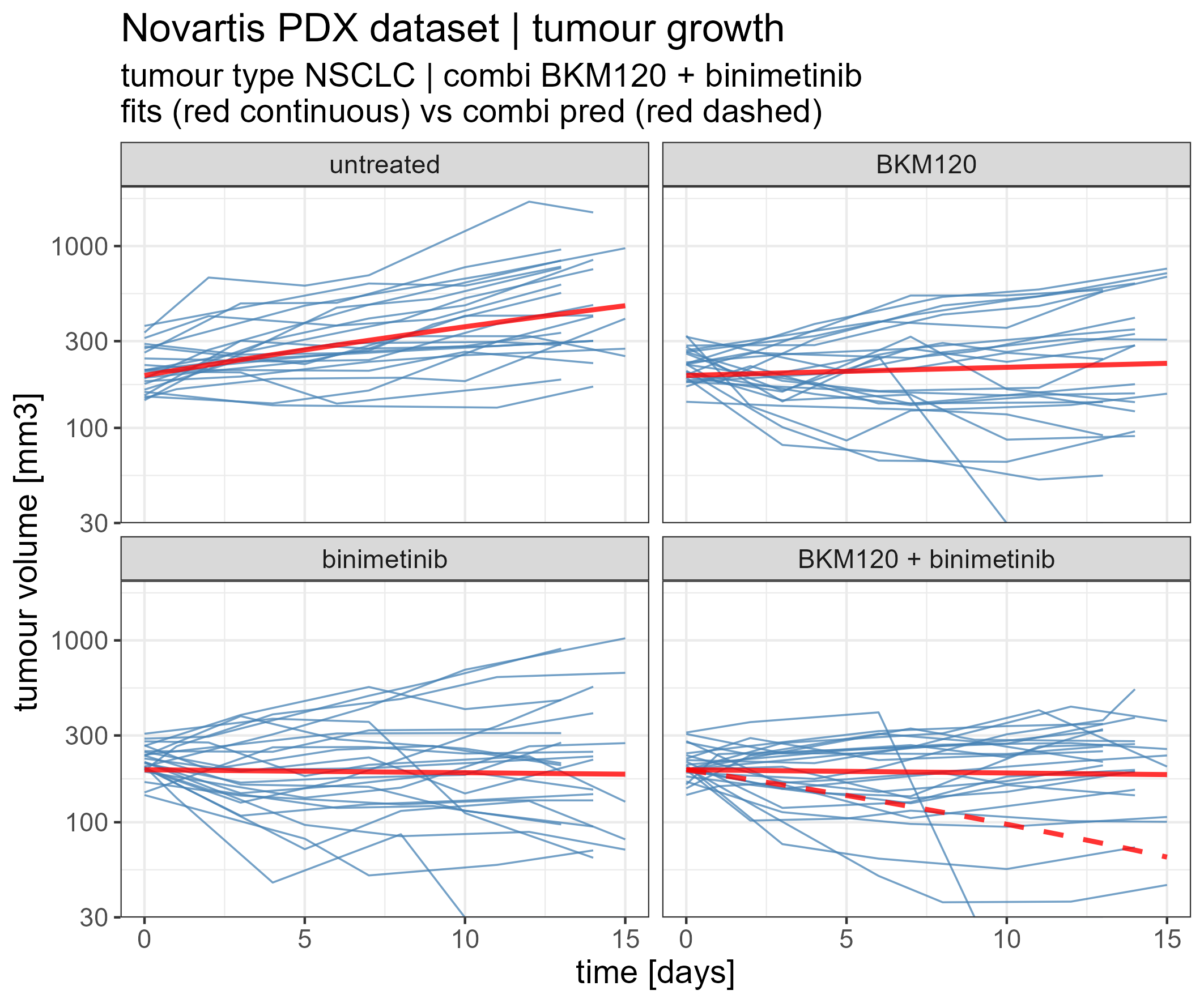

Supplement: Supplementary file 1 [file DataSheet1.ZIP › code_complete/results_PD_models_synergy_2/NSCLC_BKM120_binimetinib.png]

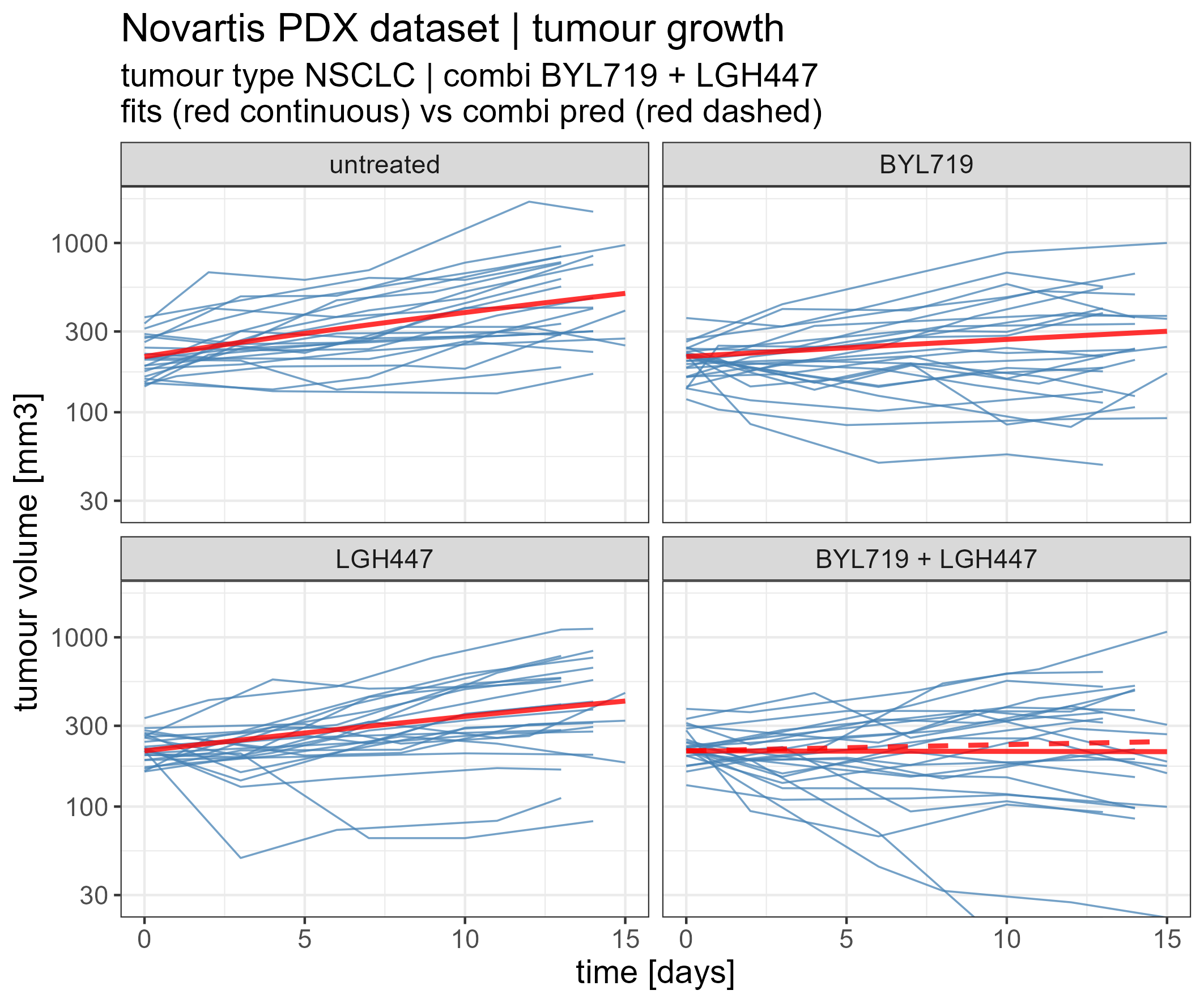

Supplement: Supplementary file 1 [file DataSheet1.ZIP › code_complete/results_PD_models_synergy_2/NSCLC_BYL719_LGH447.png]

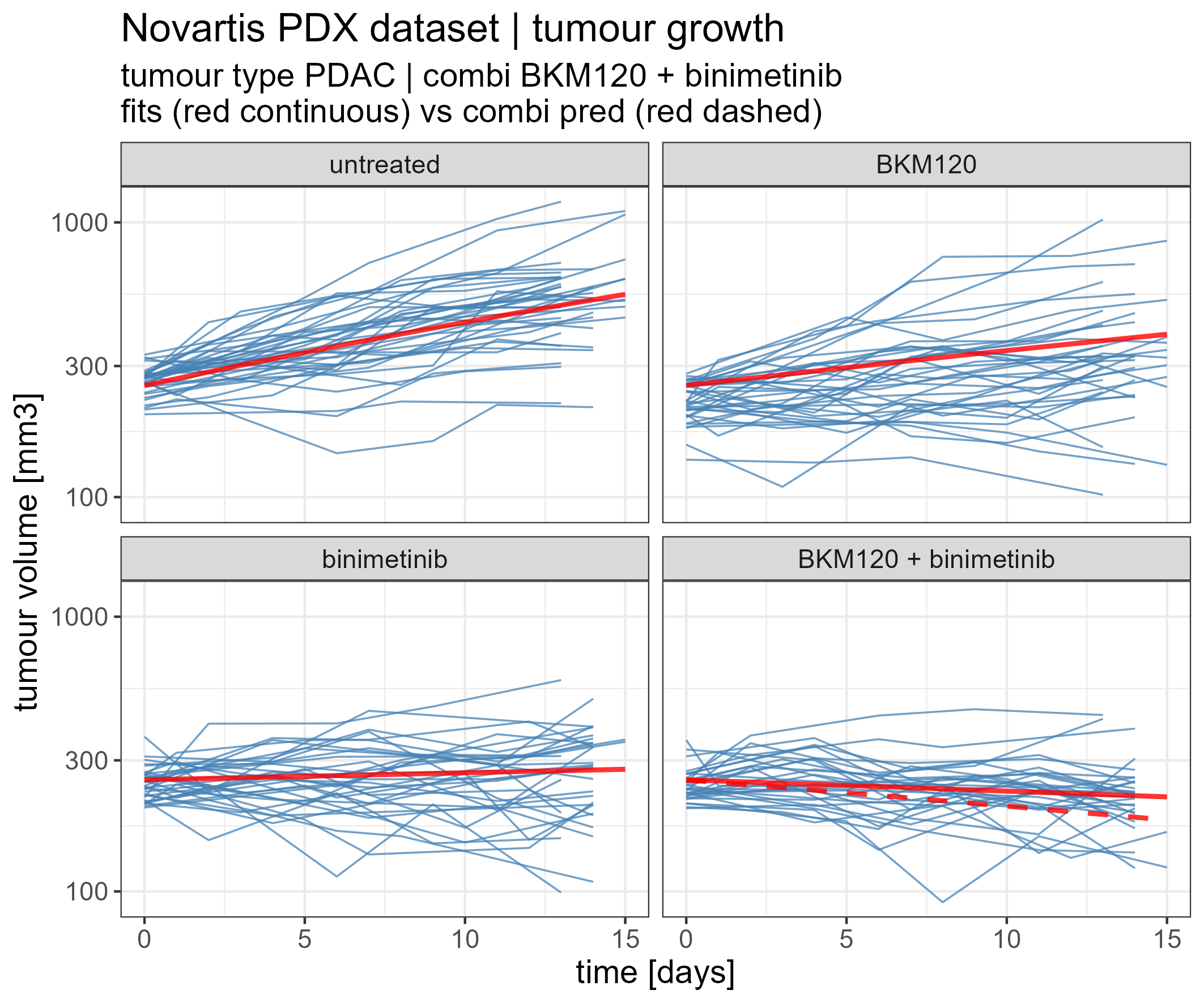

Supplement: Supplementary file 1 [file DataSheet1.ZIP › code_complete/results_PD_models_synergy_2/PDAC_BKM120_binimetinib.png]

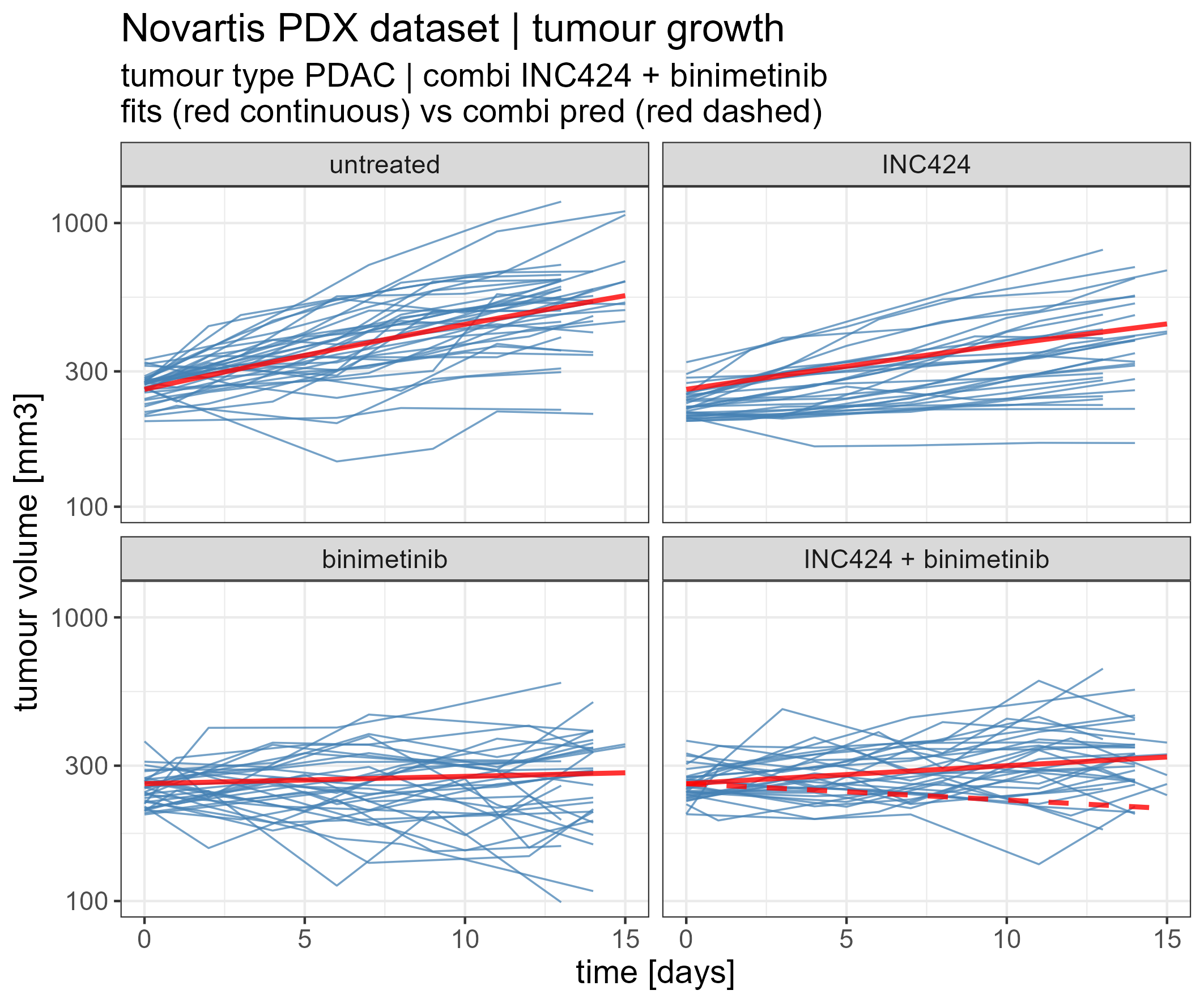

Supplement: Supplementary file 1 [file DataSheet1.ZIP › code_complete/results_PD_models_synergy_2/PDAC_INC424_binimetinib.png]

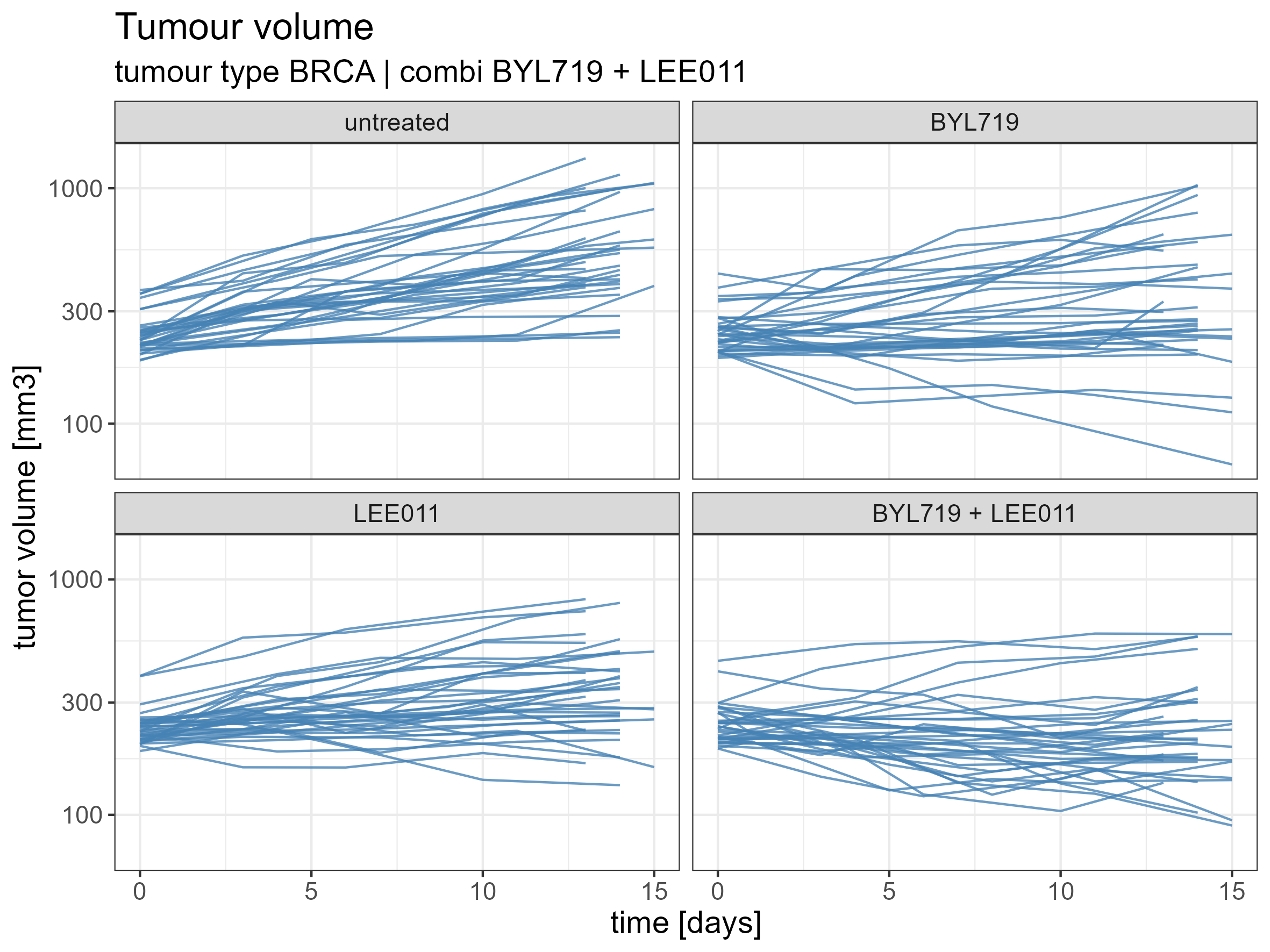

Supplement: Supplementary file 1 [file DataSheet1.ZIP › code_complete/results_plot_data/BRCA_BYL719_LEE011.png]

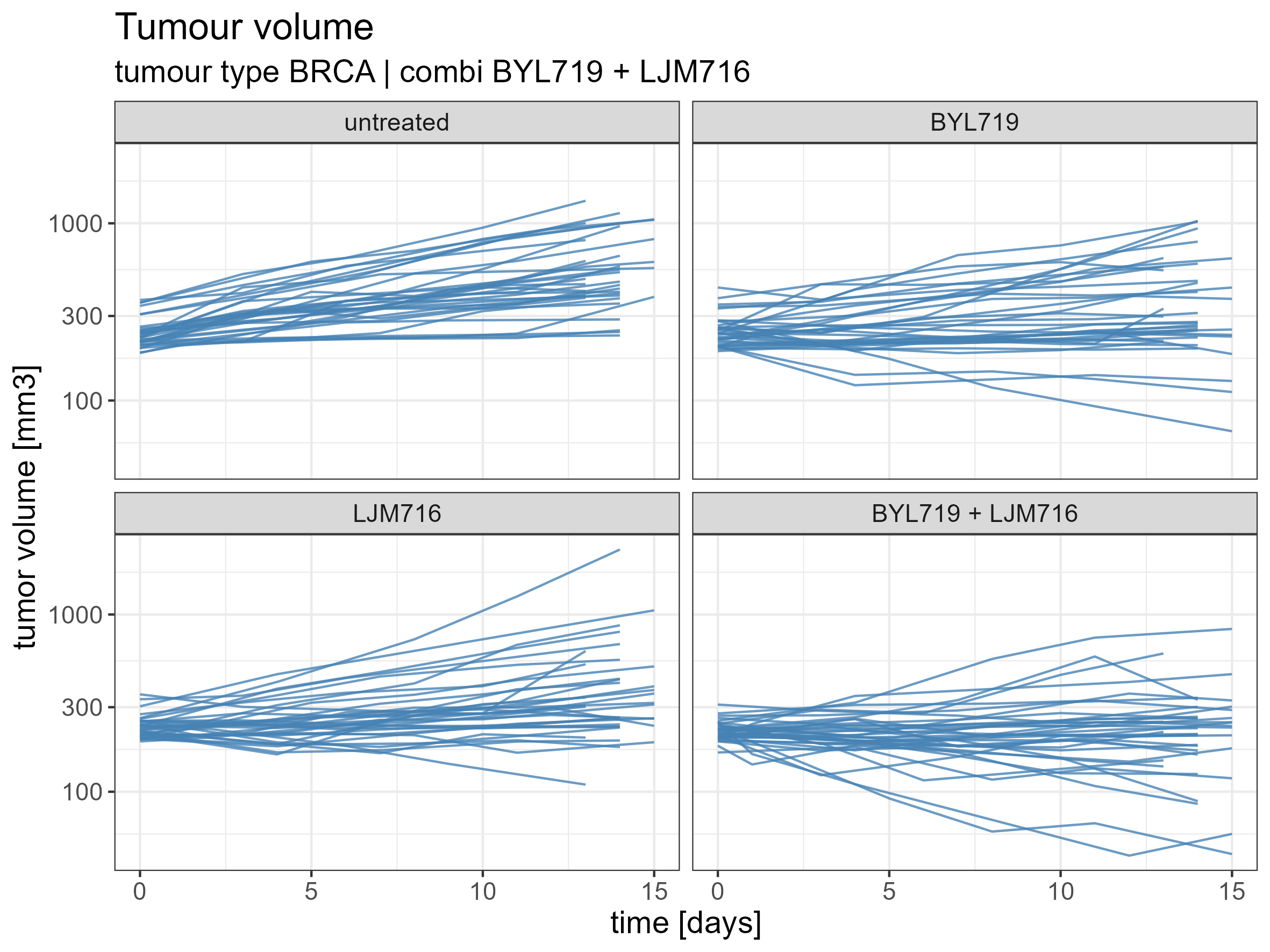

Supplement: Supplementary file 1 [file DataSheet1.ZIP › code_complete/results_plot_data/BRCA_BYL719_LJM716.png]

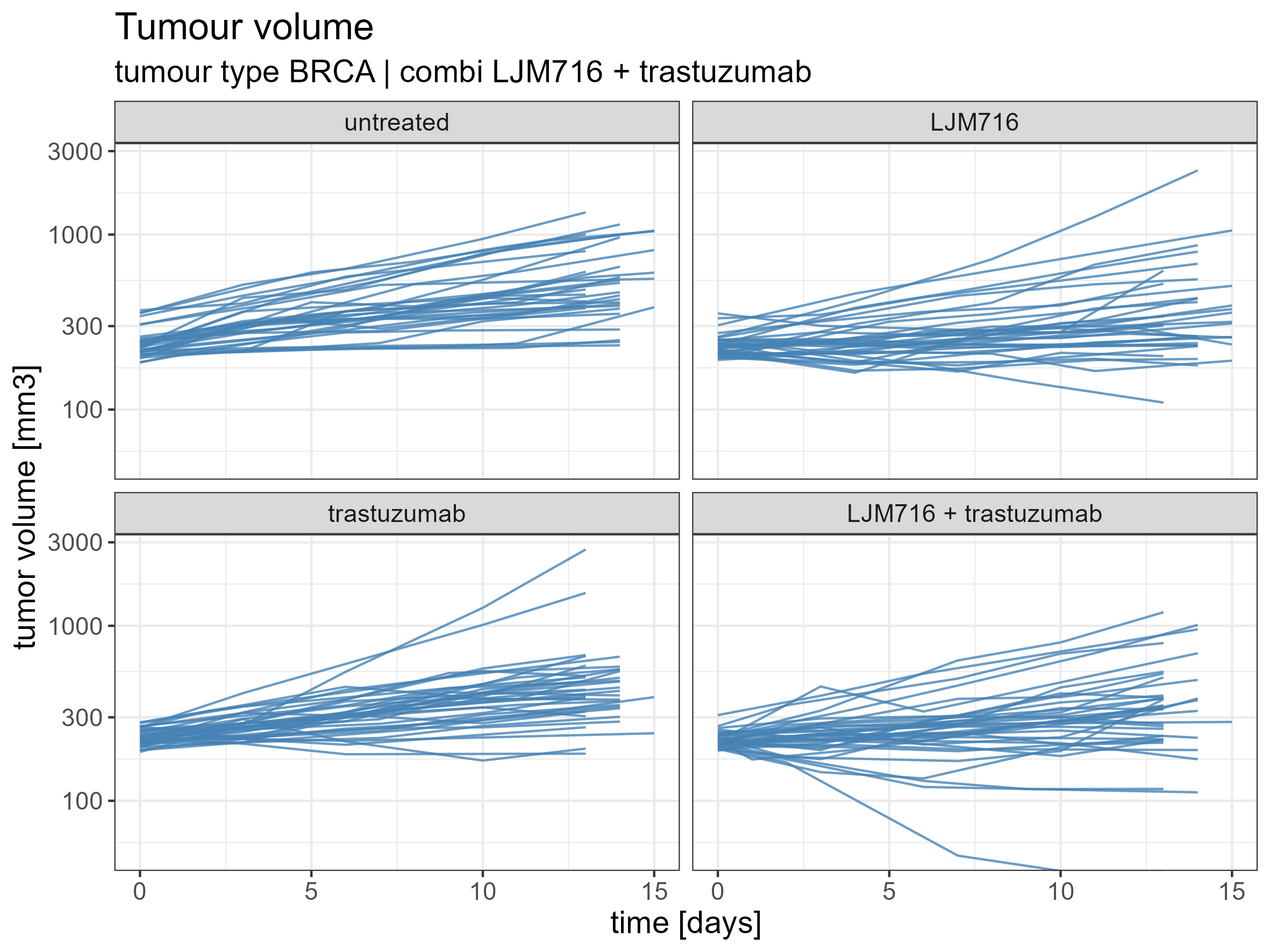

Supplement: Supplementary file 1 [file DataSheet1.ZIP › code_complete/results_plot_data/BRCA_LJM716_trastuzumab.png]

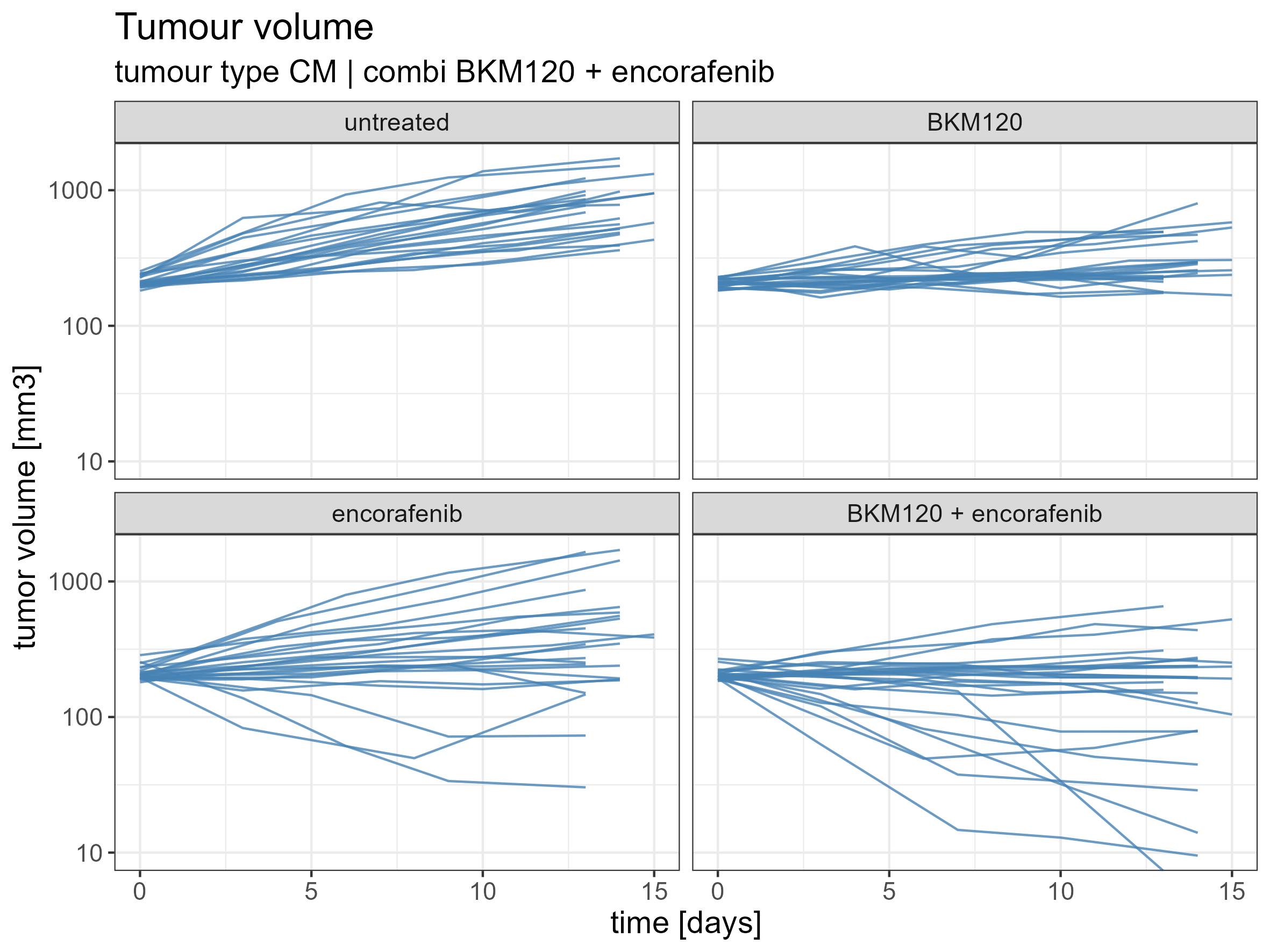

Supplement: Supplementary file 1 [file DataSheet1.ZIP › code_complete/results_plot_data/CM_BKM120_encorafenib.png]

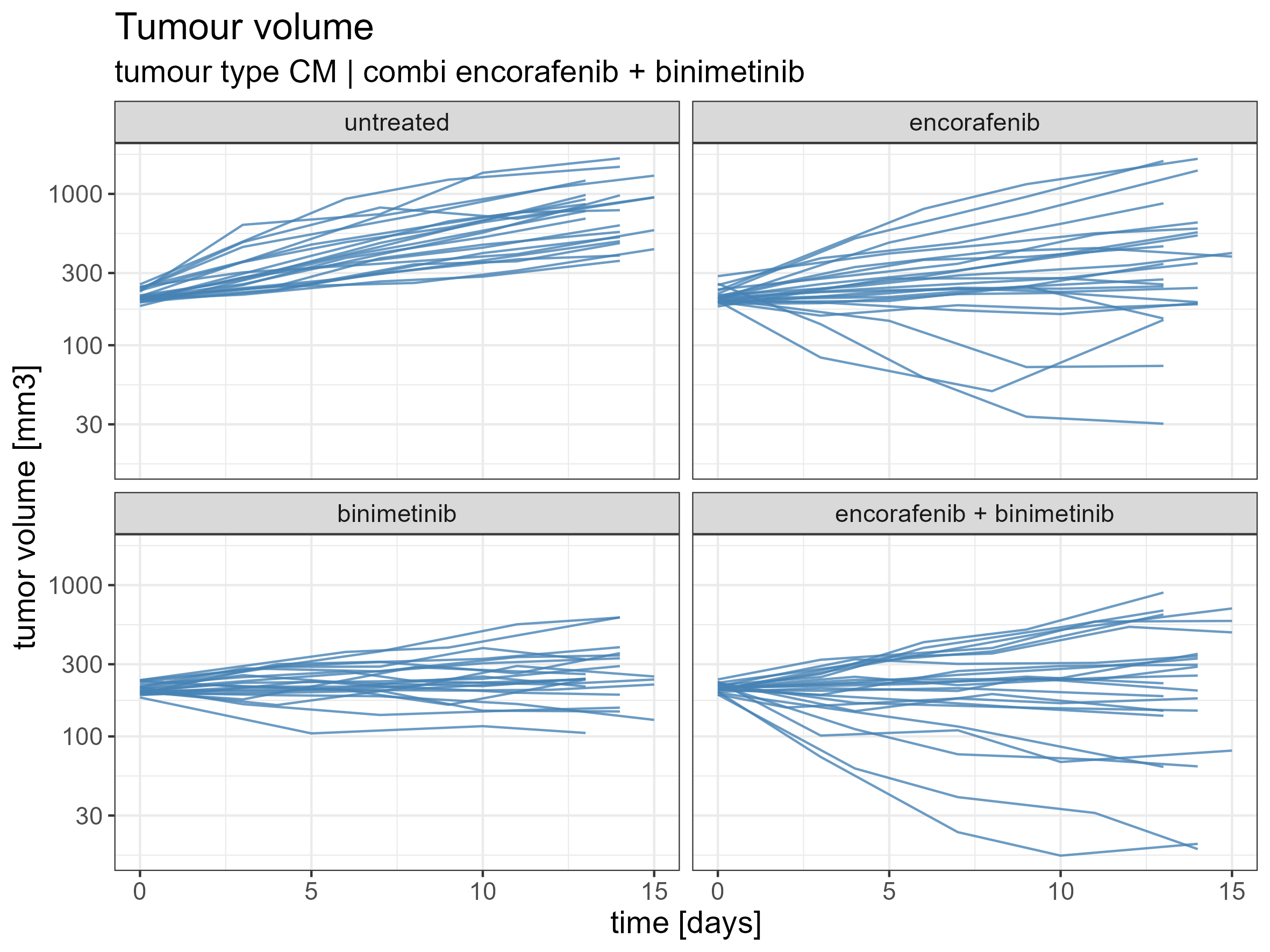

Supplement: Supplementary file 1 [file DataSheet1.ZIP › code_complete/results_plot_data/CM_encorafenib_binimetinib.png]

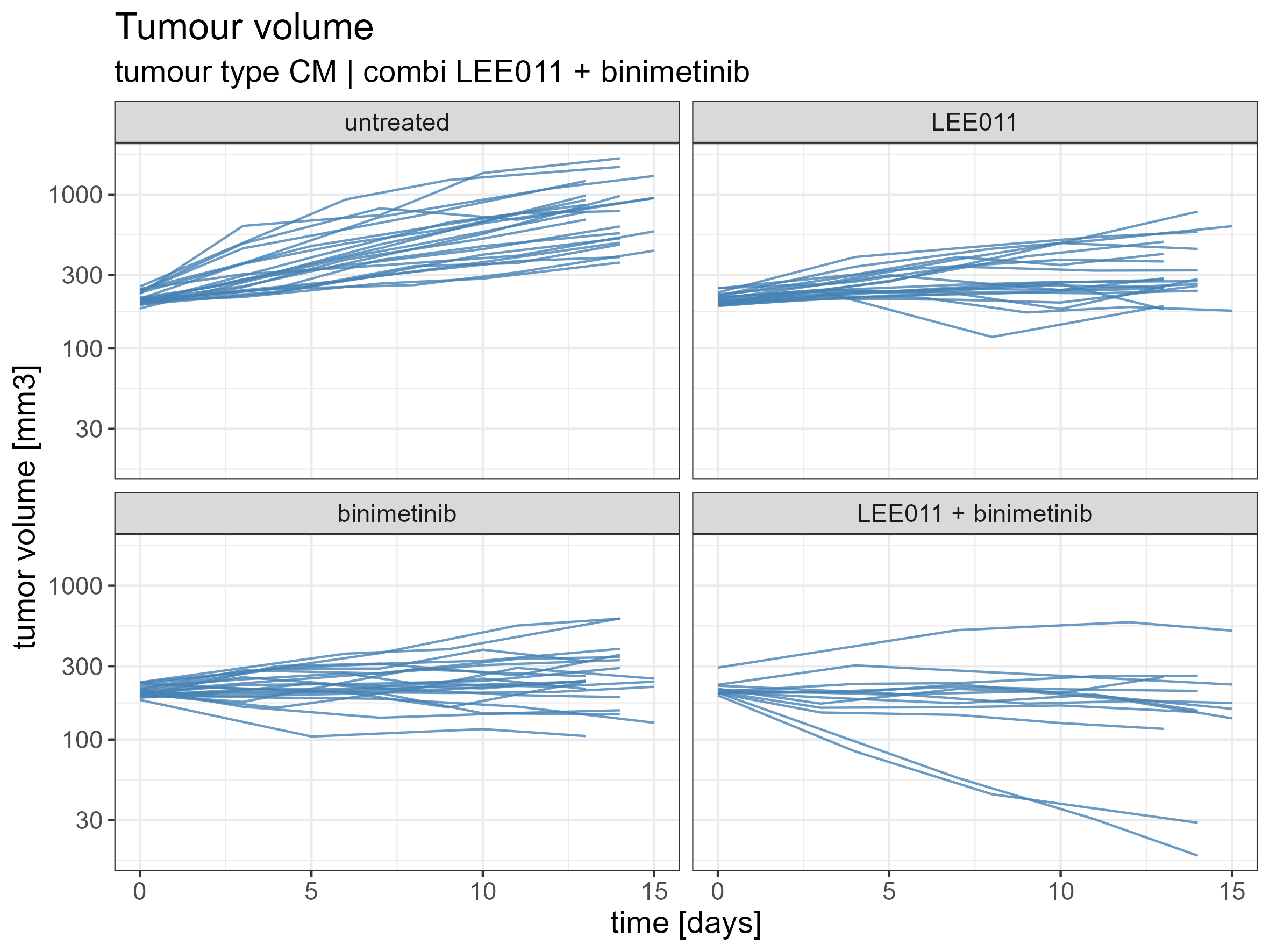

Supplement: Supplementary file 1 [file DataSheet1.ZIP › code_complete/results_plot_data/CM_LEE011_binimetinib.png]

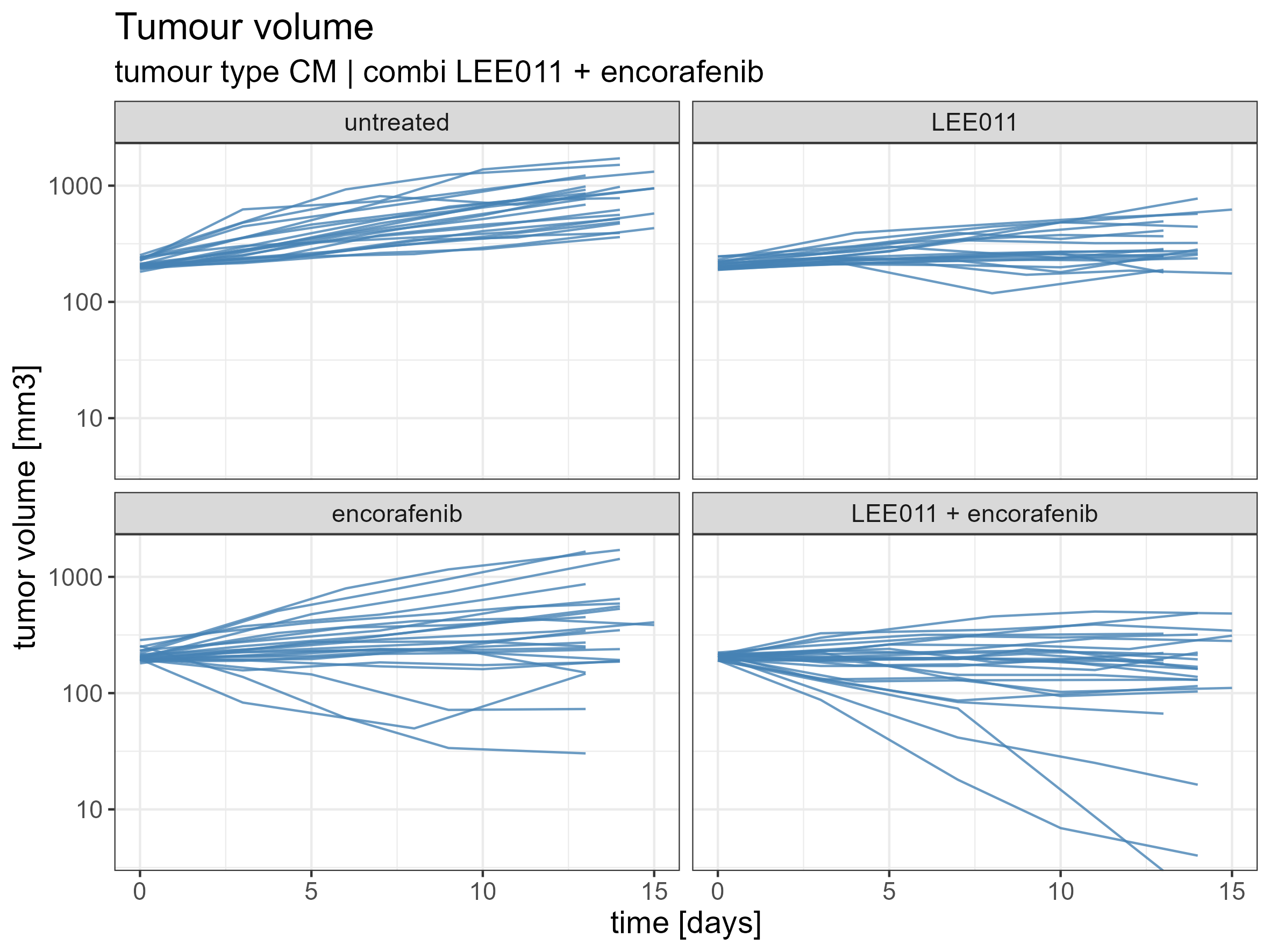

Supplement: Supplementary file 1 [file DataSheet1.ZIP › code_complete/results_plot_data/CM_LEE011_encorafenib.png]

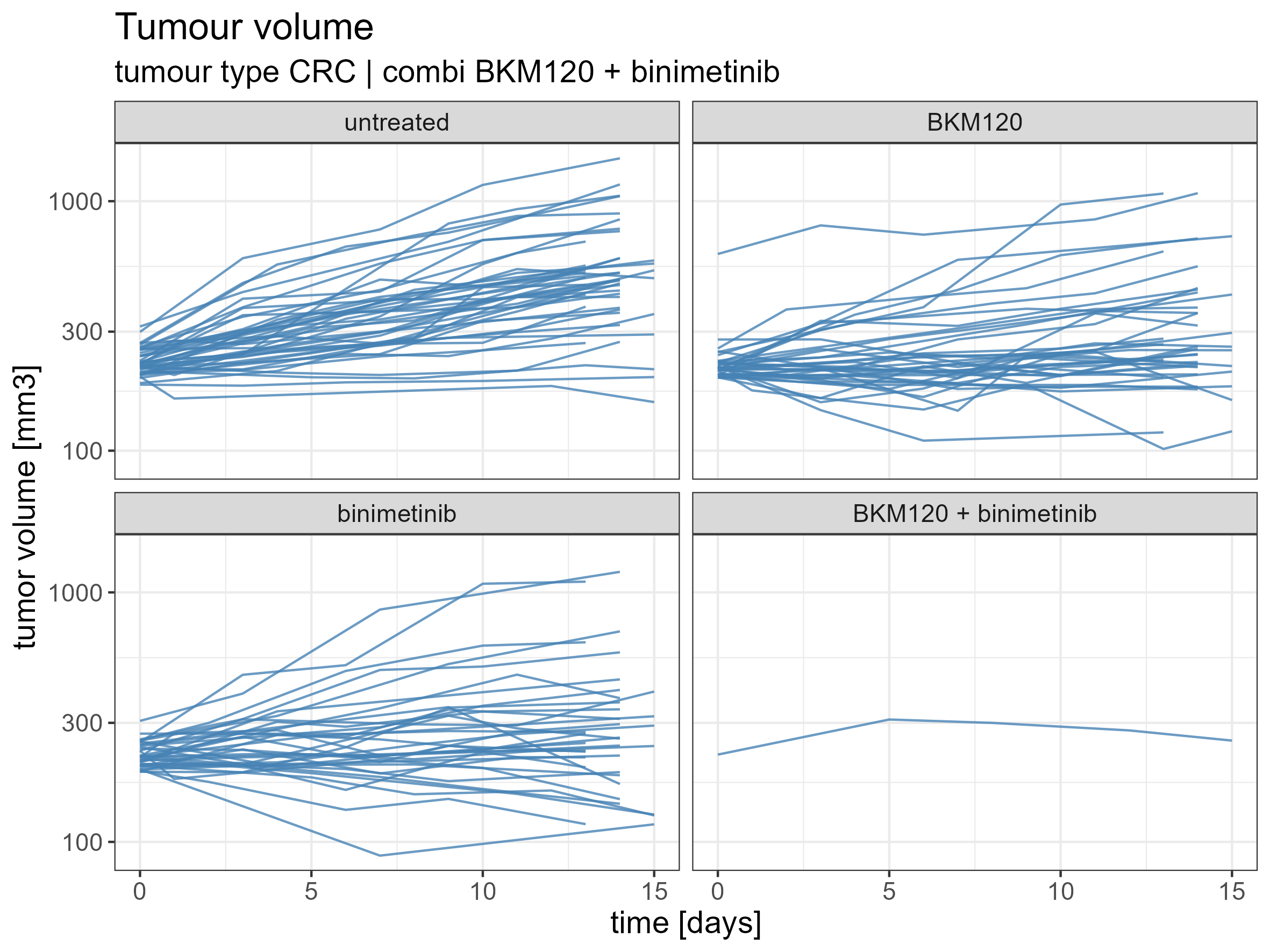

Supplement: Supplementary file 1 [file DataSheet1.ZIP › code_complete/results_plot_data/CRC_BKM120_binimetinib.png]

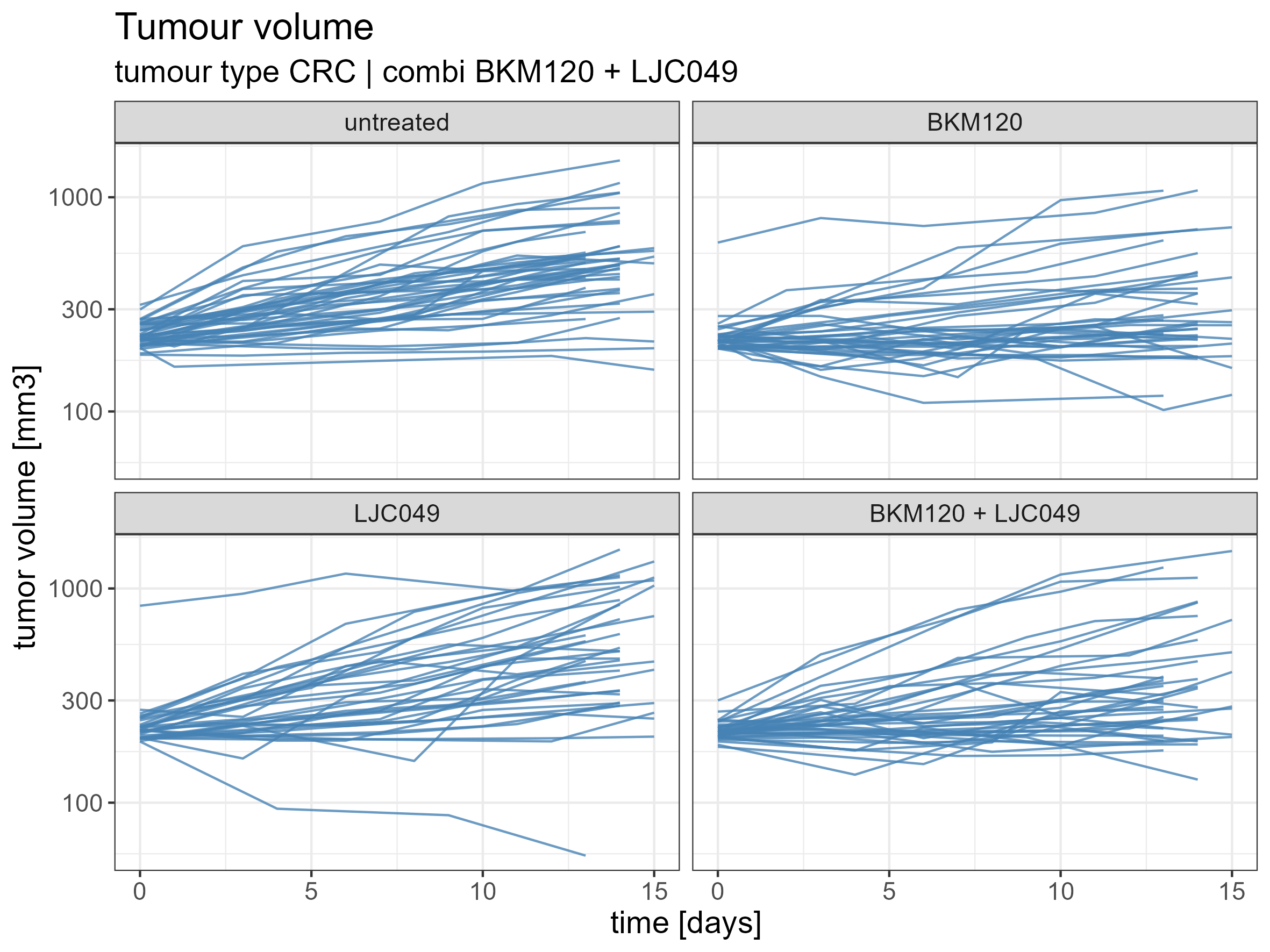

Supplement: Supplementary file 1 [file DataSheet1.ZIP › code_complete/results_plot_data/CRC_BKM120_LJC049.png]

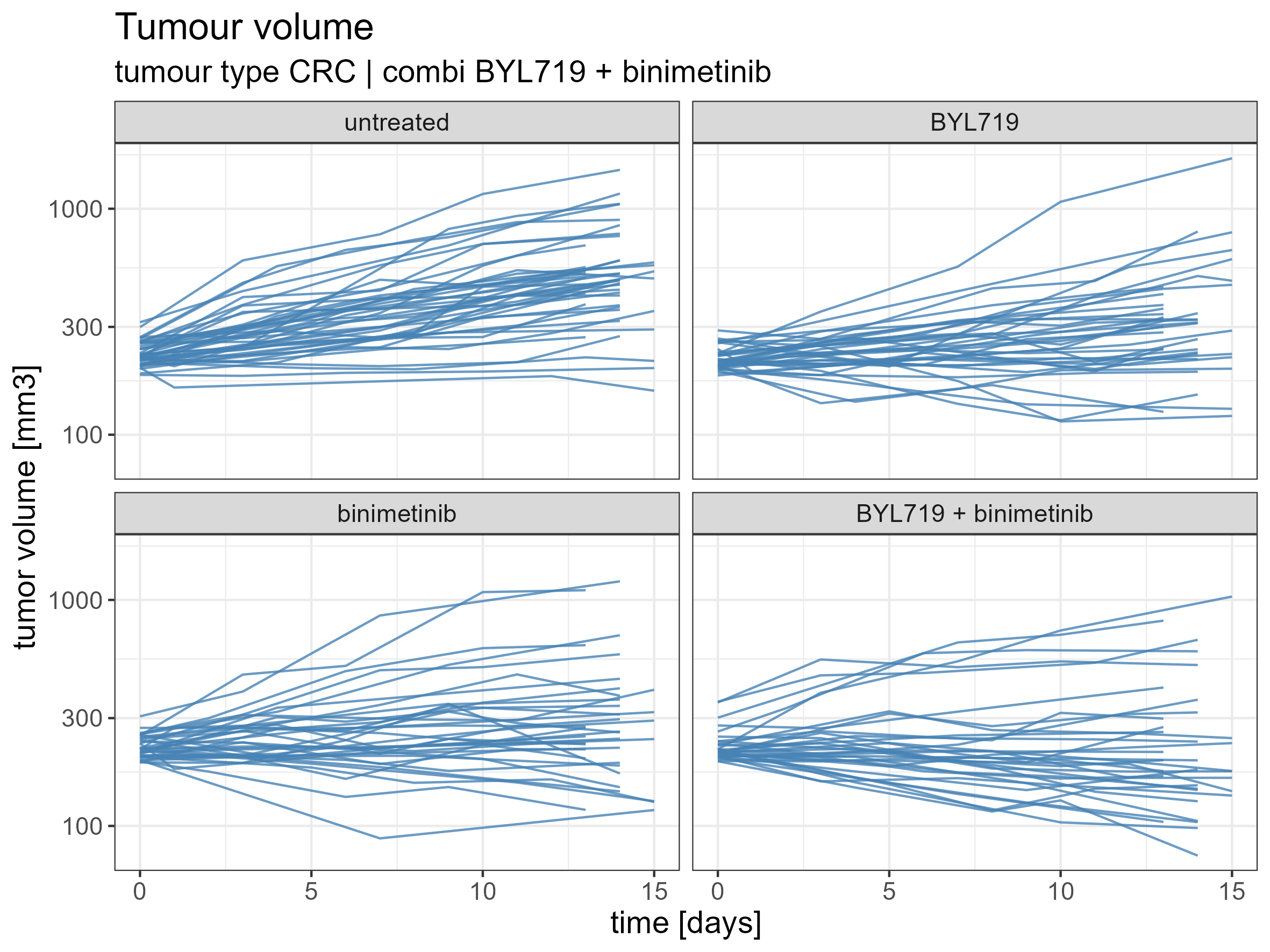

Supplement: Supplementary file 1 [file DataSheet1.ZIP › code_complete/results_plot_data/CRC_BYL719_binimetinib.png]

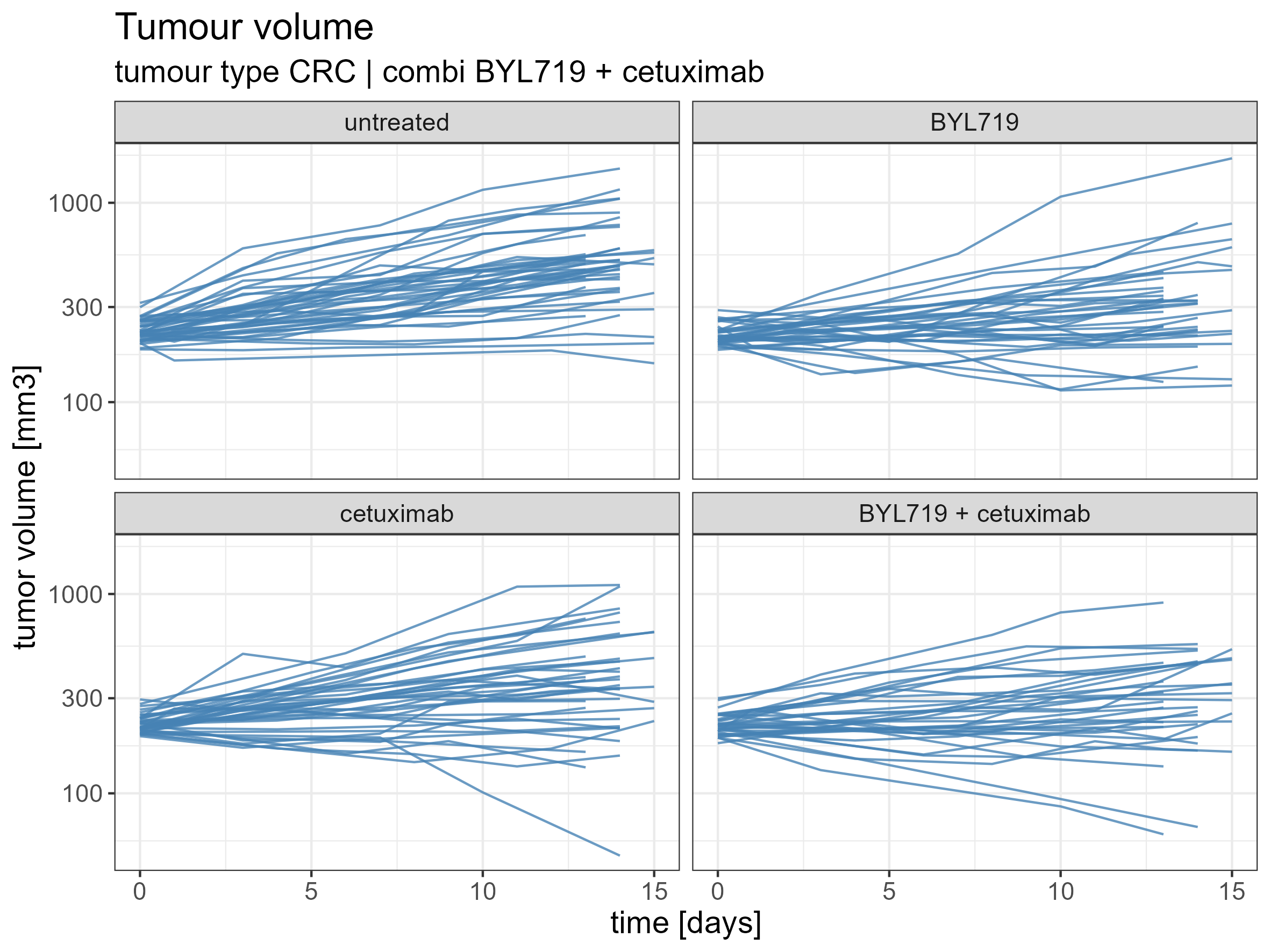

Supplement: Supplementary file 1 [file DataSheet1.ZIP › code_complete/results_plot_data/CRC_BYL719_cetuximab.png]

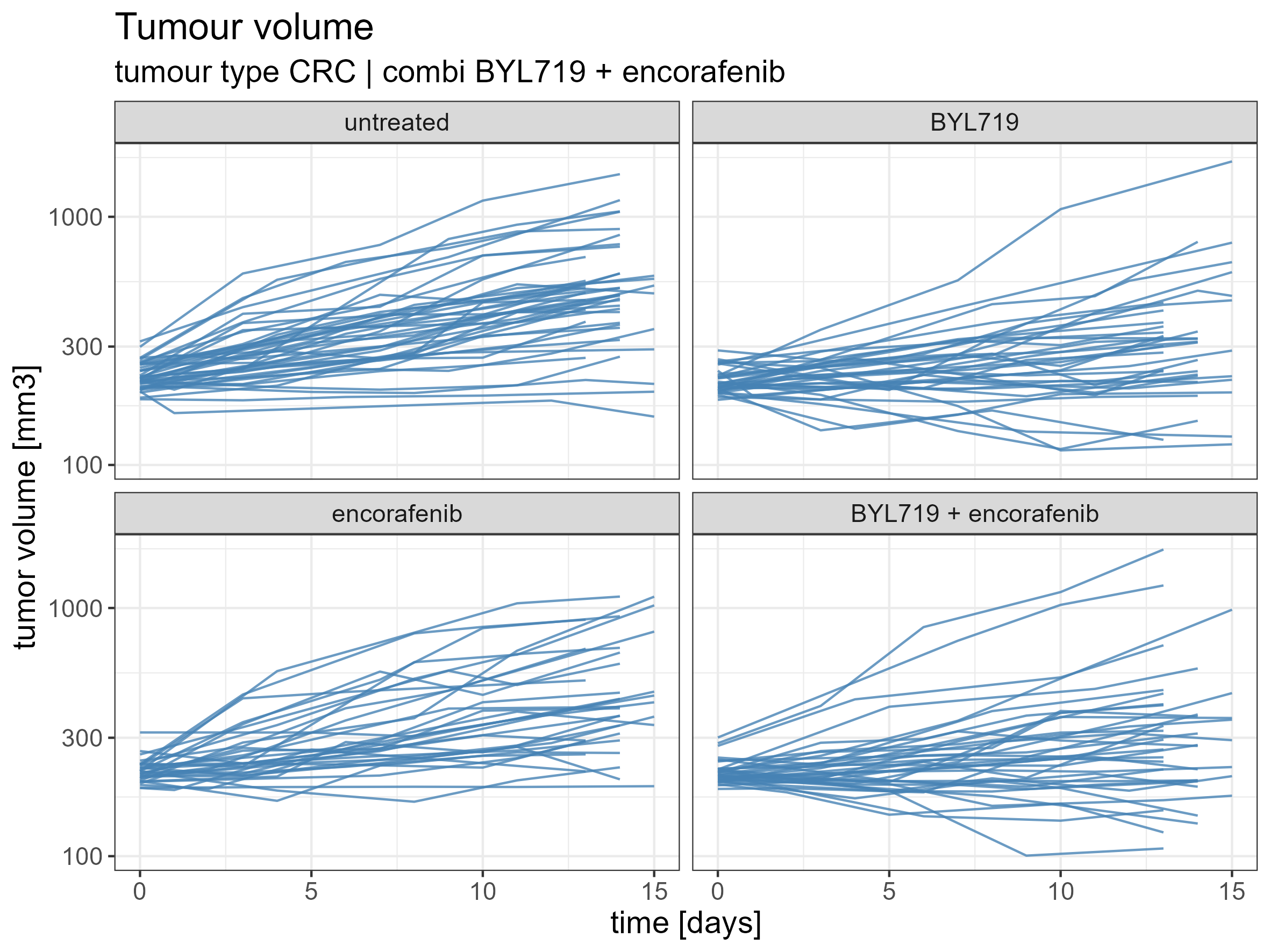

Supplement: Supplementary file 1 [file DataSheet1.ZIP › code_complete/results_plot_data/CRC_BYL719_encorafenib.png]

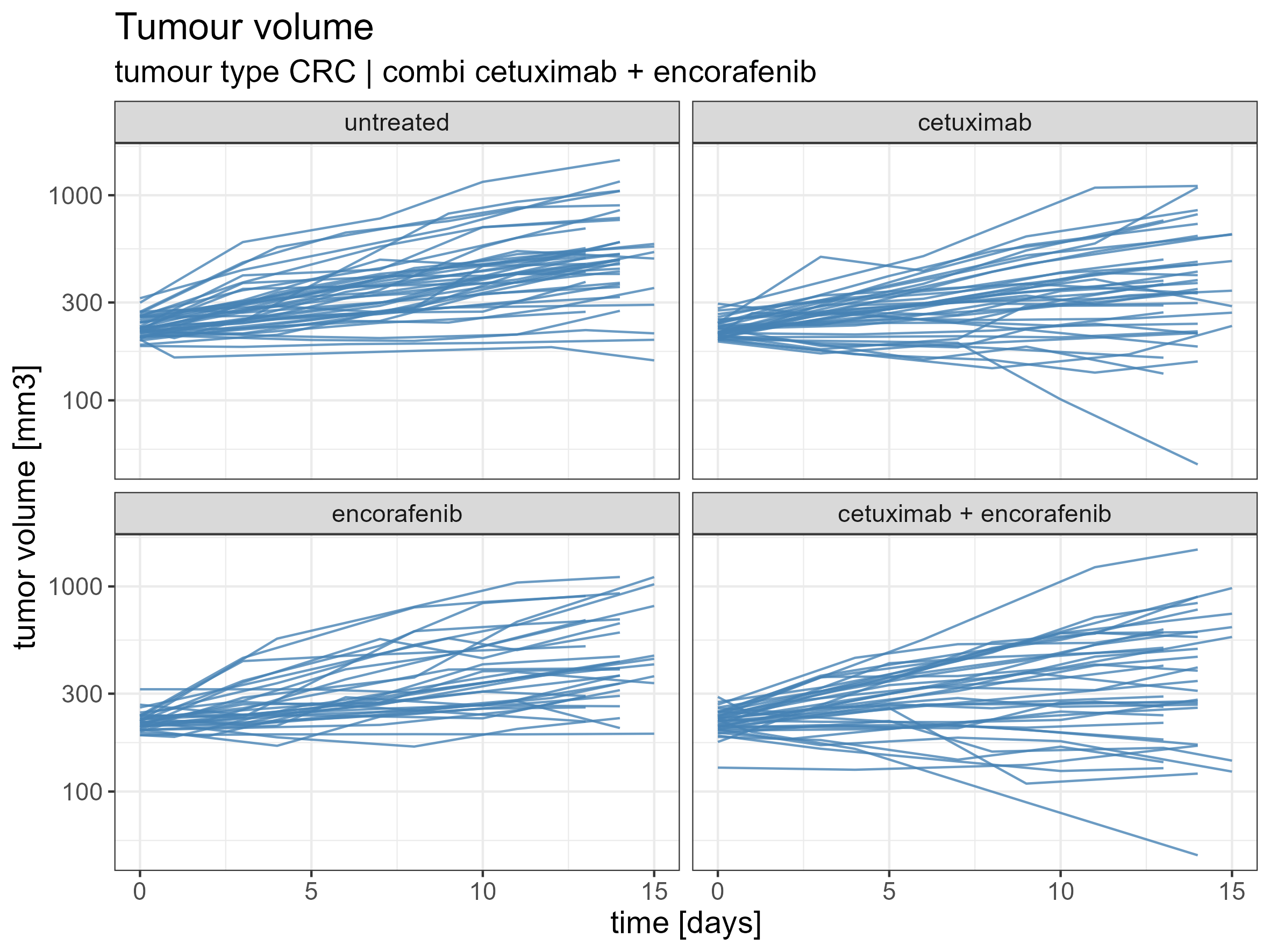

Supplement: Supplementary file 1 [file DataSheet1.ZIP › code_complete/results_plot_data/CRC_cetuximab_encorafenib.png]

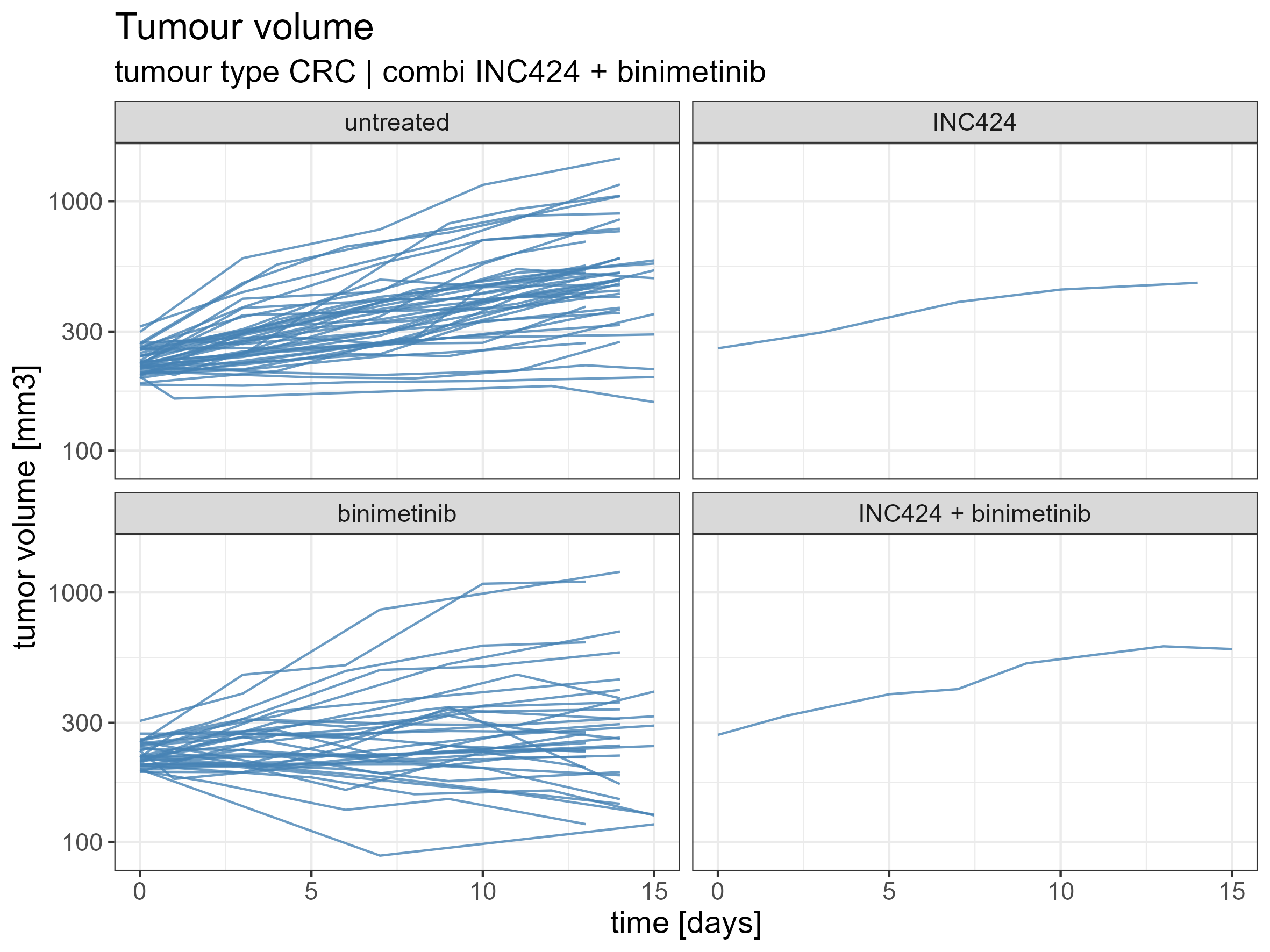

Supplement: Supplementary file 1 [file DataSheet1.ZIP › code_complete/results_plot_data/CRC_INC424_binimetinib.png]

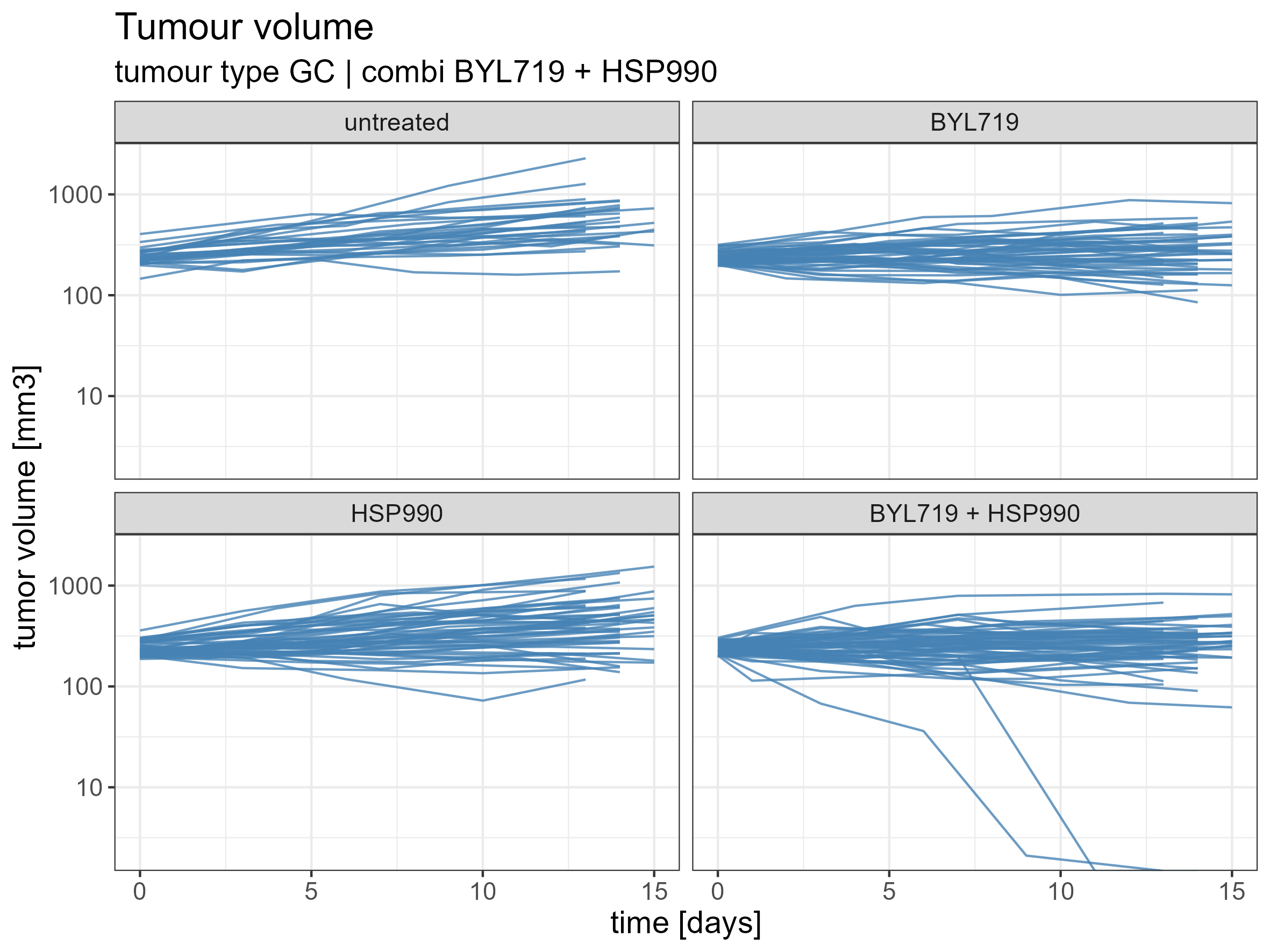

Supplement: Supplementary file 1 [file DataSheet1.ZIP › code_complete/results_plot_data/GC_BYL719_HSP990.png]

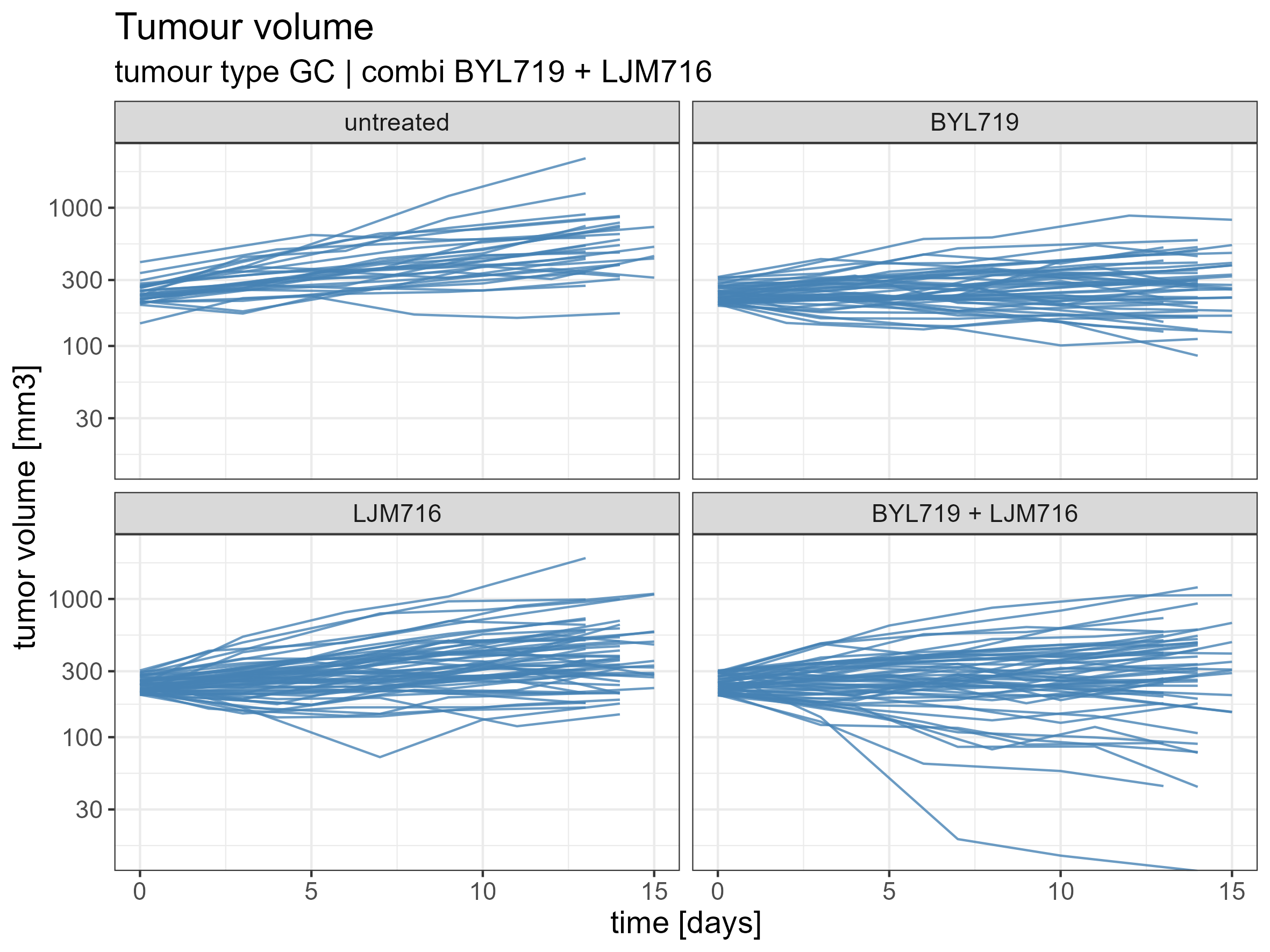

Supplement: Supplementary file 1 [file DataSheet1.ZIP › code_complete/results_plot_data/GC_BYL719_LJM716.png]

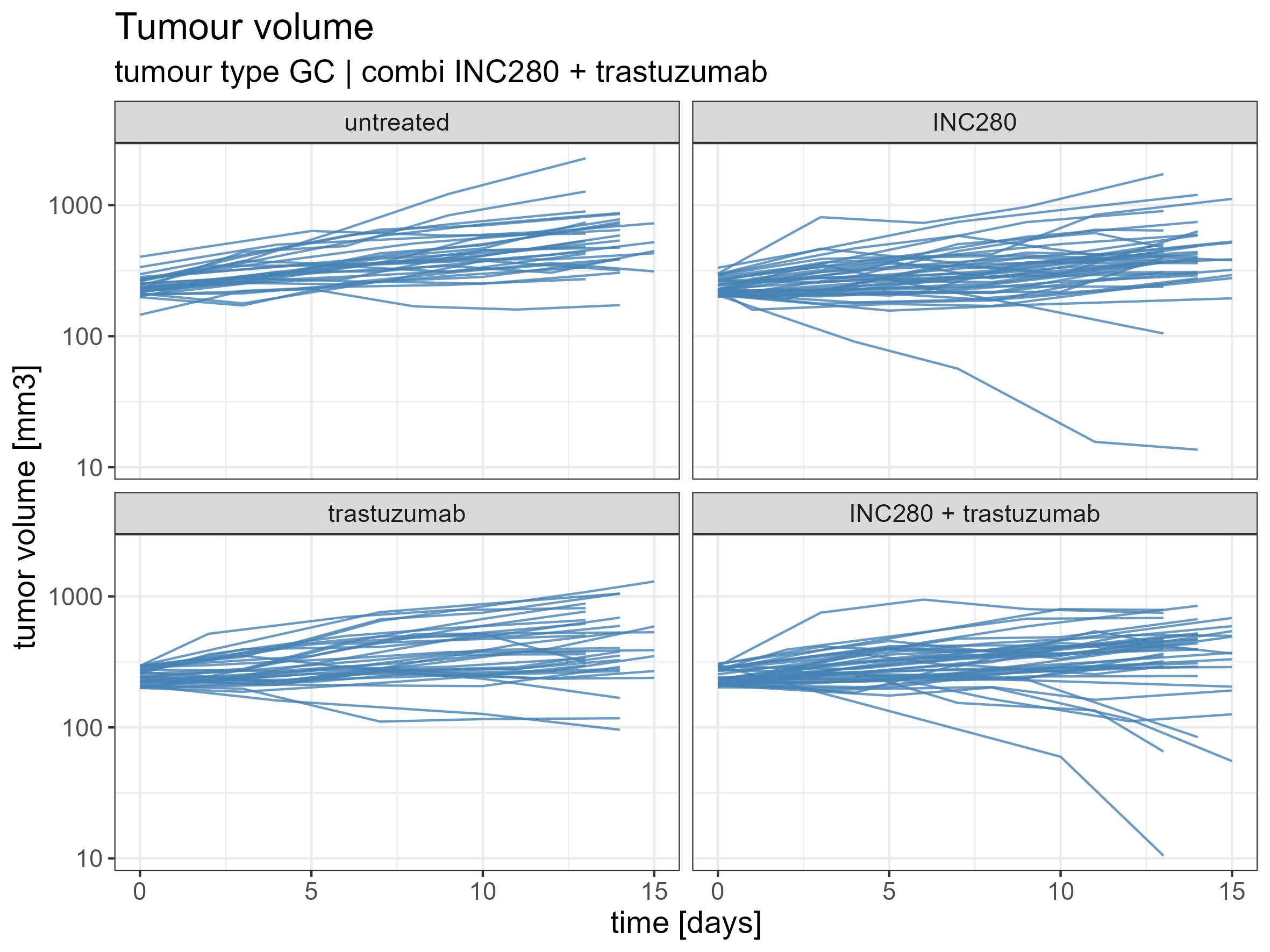

Supplement: Supplementary file 1 [file DataSheet1.ZIP › code_complete/results_plot_data/GC_INC280_trastuzumab.png]

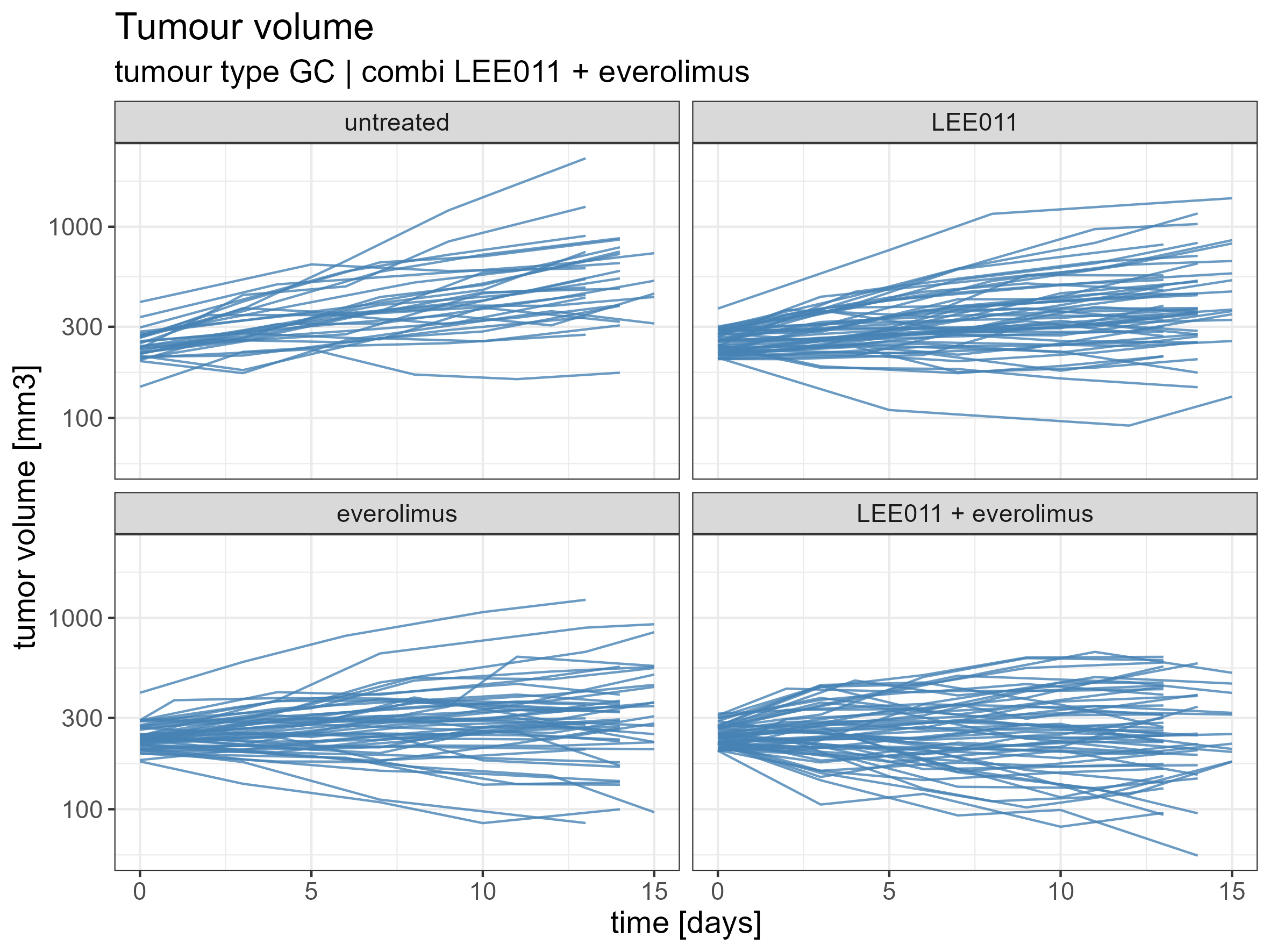

Supplement: Supplementary file 1 [file DataSheet1.ZIP › code_complete/results_plot_data/GC_LEE011_everolimus.png]

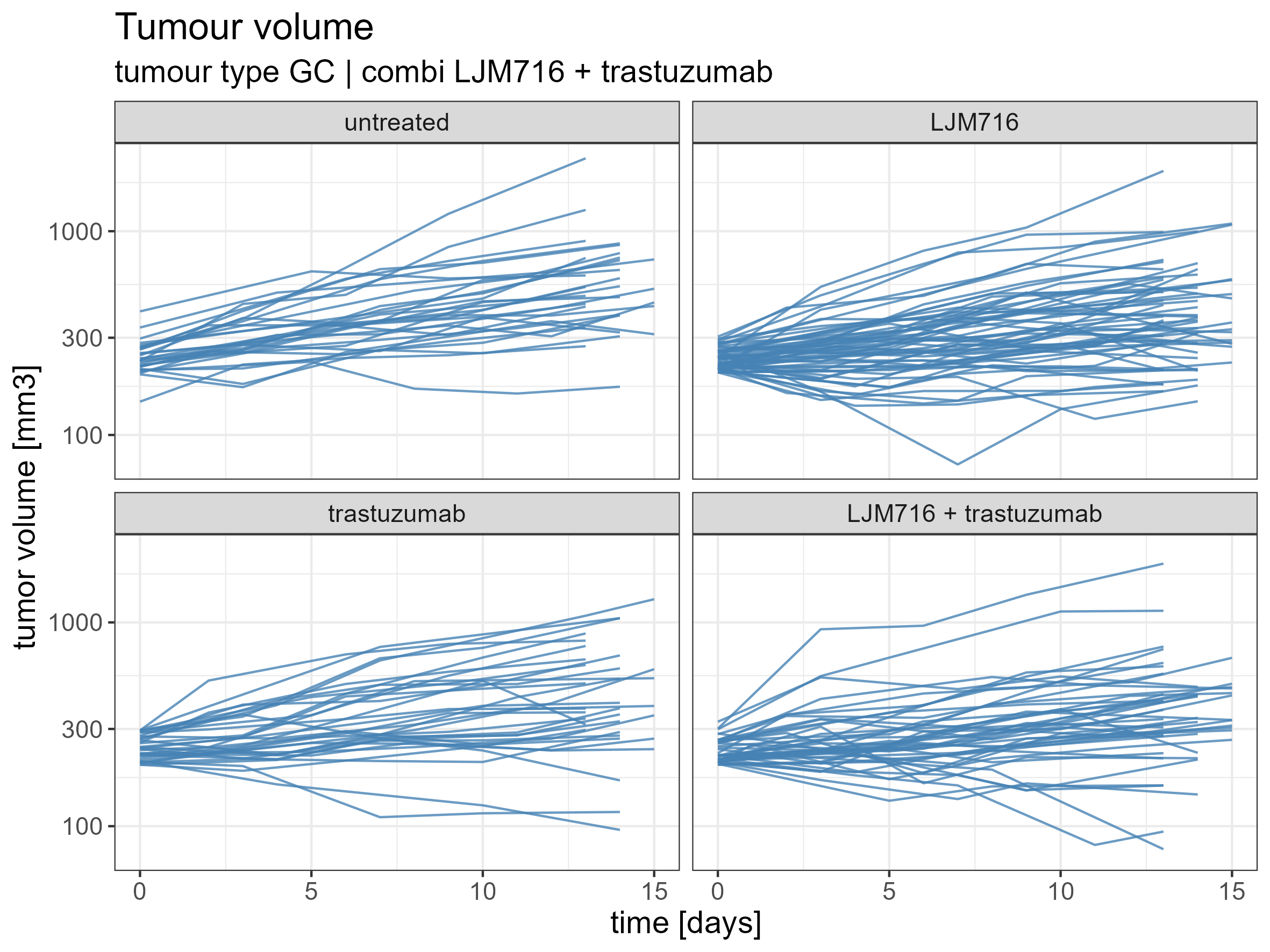

Supplement: Supplementary file 1 [file DataSheet1.ZIP › code_complete/results_plot_data/GC_LJM716_trastuzumab.png]

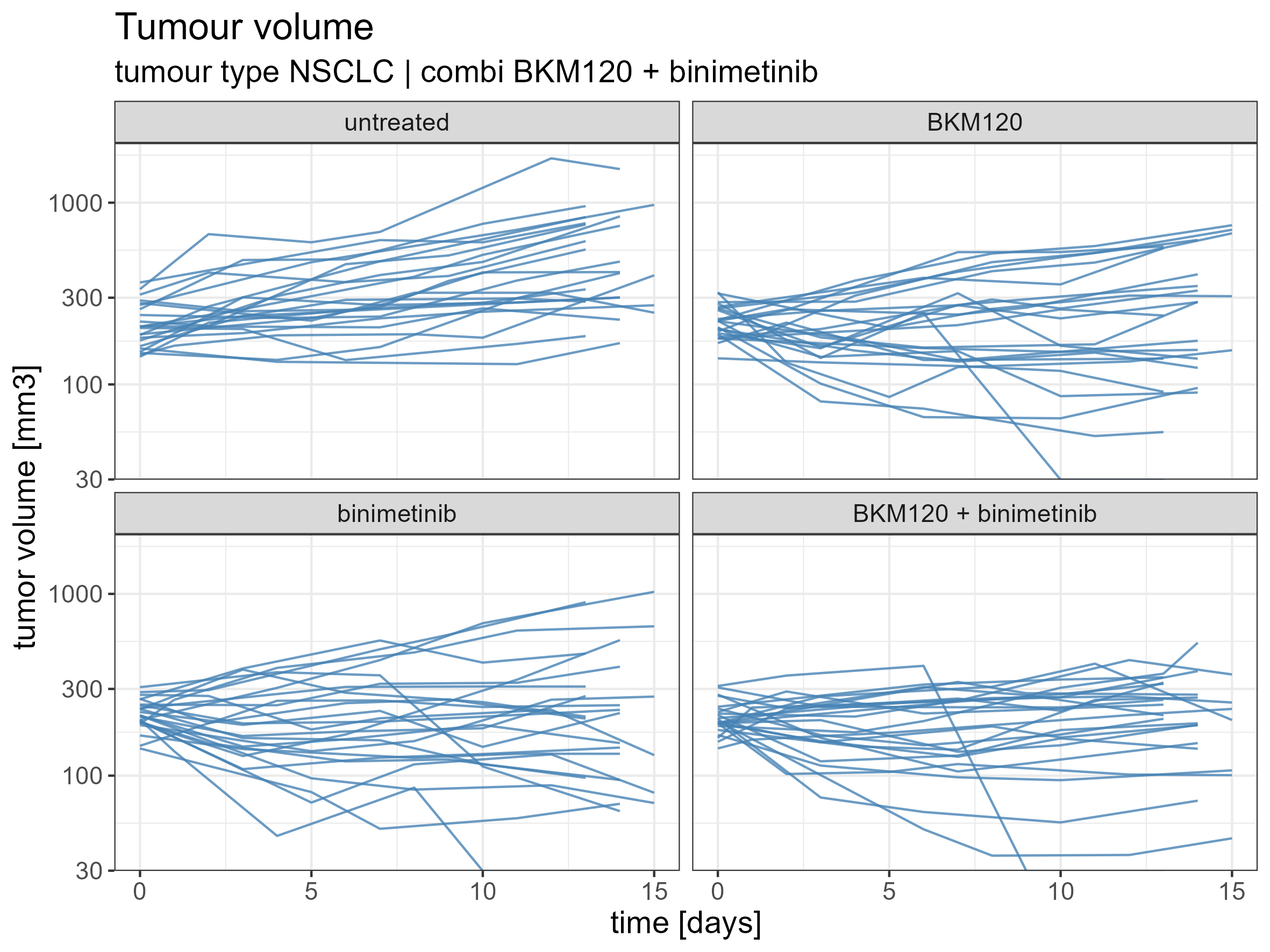

Supplement: Supplementary file 1 [file DataSheet1.ZIP › code_complete/results_plot_data/NSCLC_BKM120_binimetinib.png]

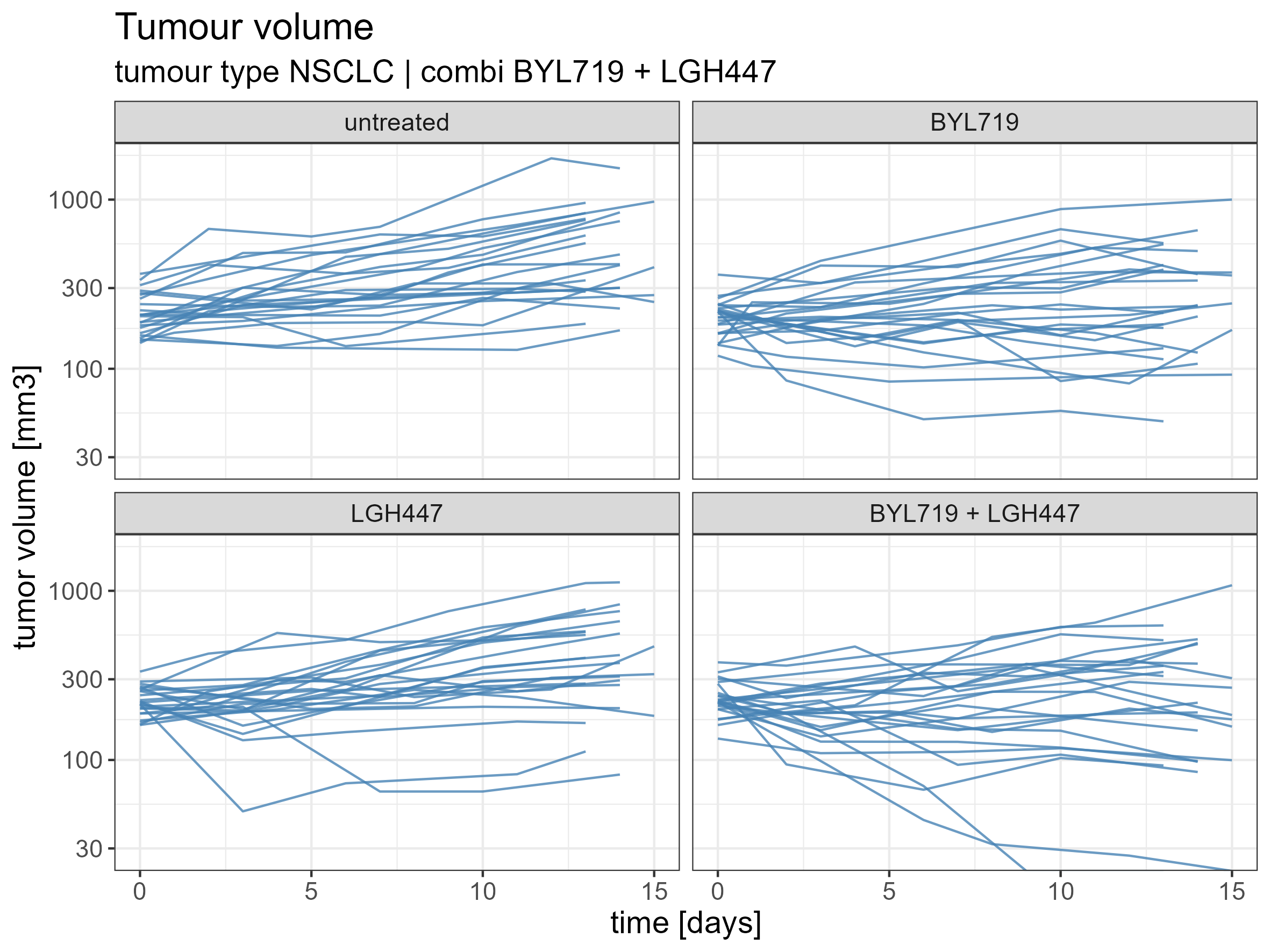

Supplement: Supplementary file 1 [file DataSheet1.ZIP › code_complete/results_plot_data/NSCLC_BYL719_LGH447.png]

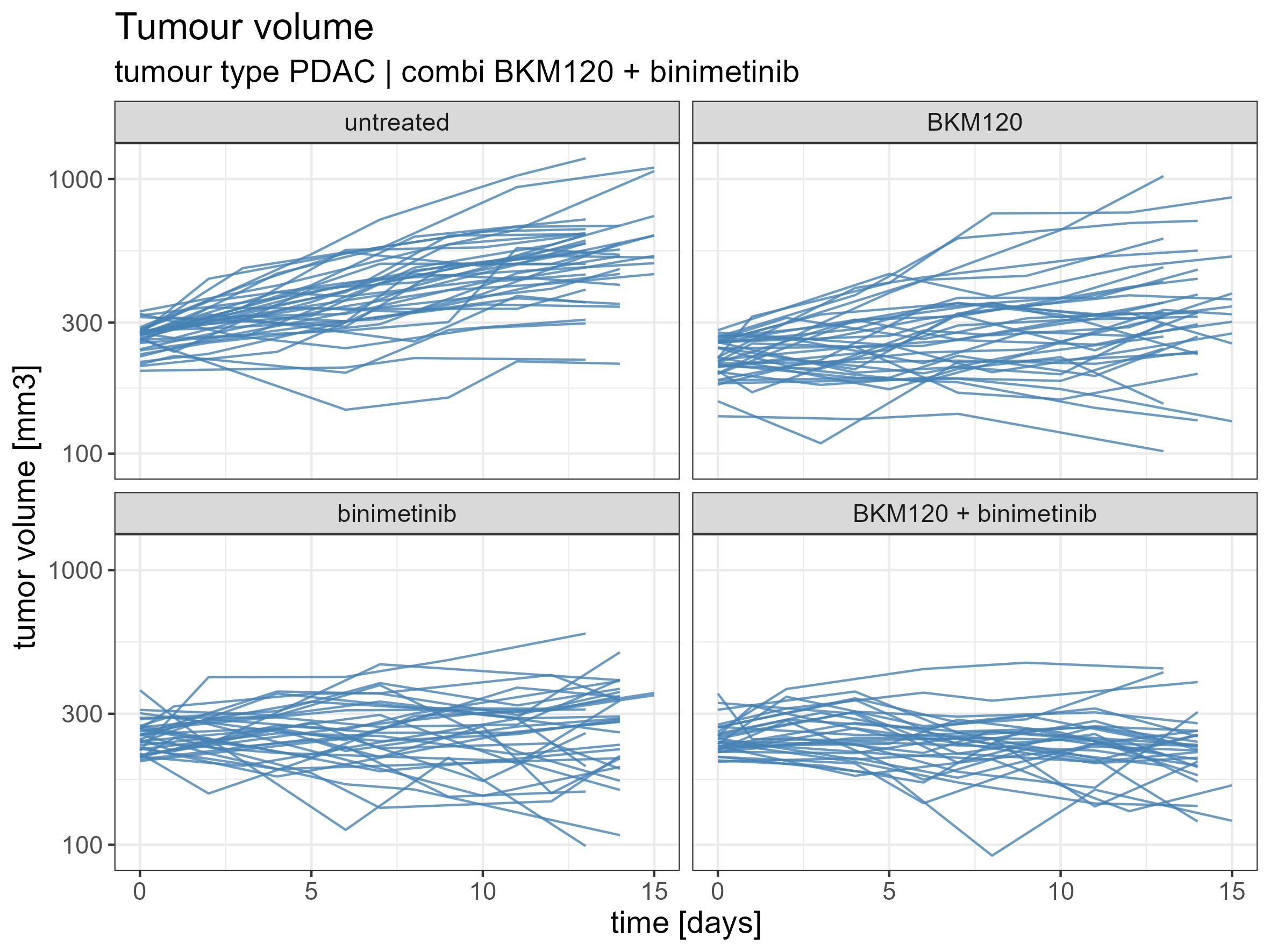

Supplement: Supplementary file 1 [file DataSheet1.ZIP › code_complete/results_plot_data/PDAC_BKM120_binimetinib.png]

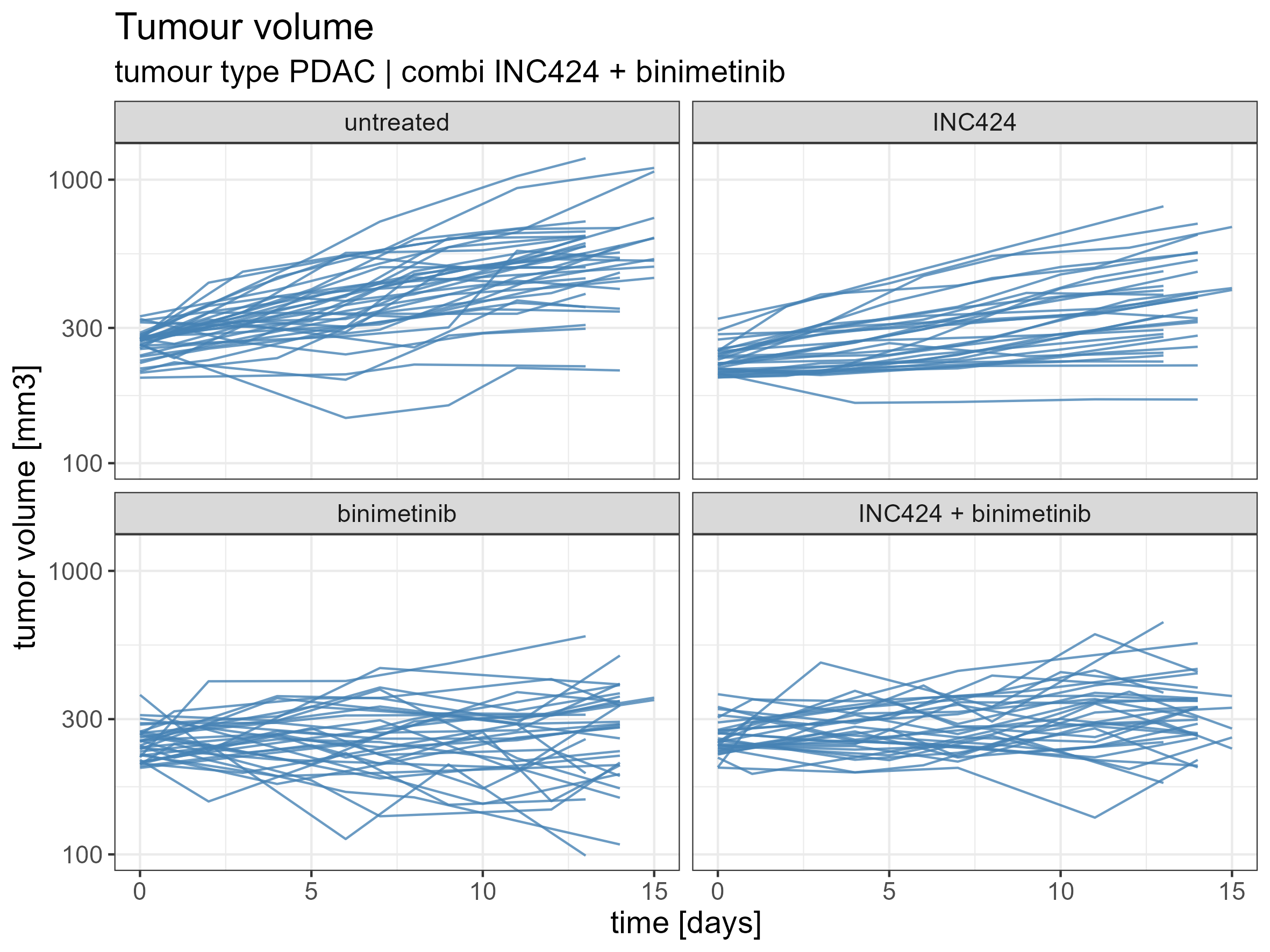

Supplement: Supplementary file 1 [file DataSheet1.ZIP › code_complete/results_plot_data/PDAC_INC424_binimetinib.png]

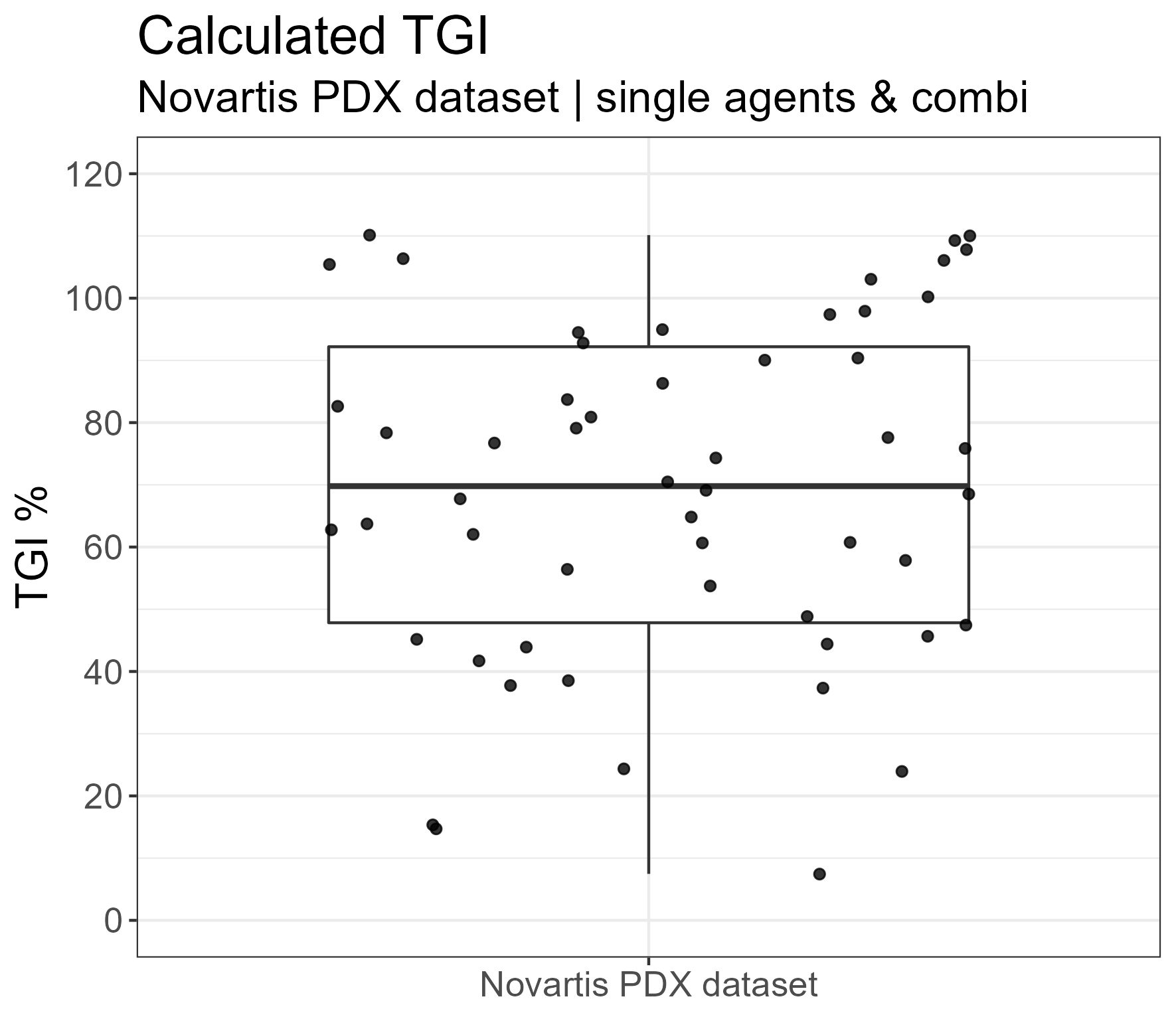

Supplement: Supplementary file 1 [file DataSheet1.ZIP › code_complete/results_TGI_combi_2/calc_TGI_all.png]

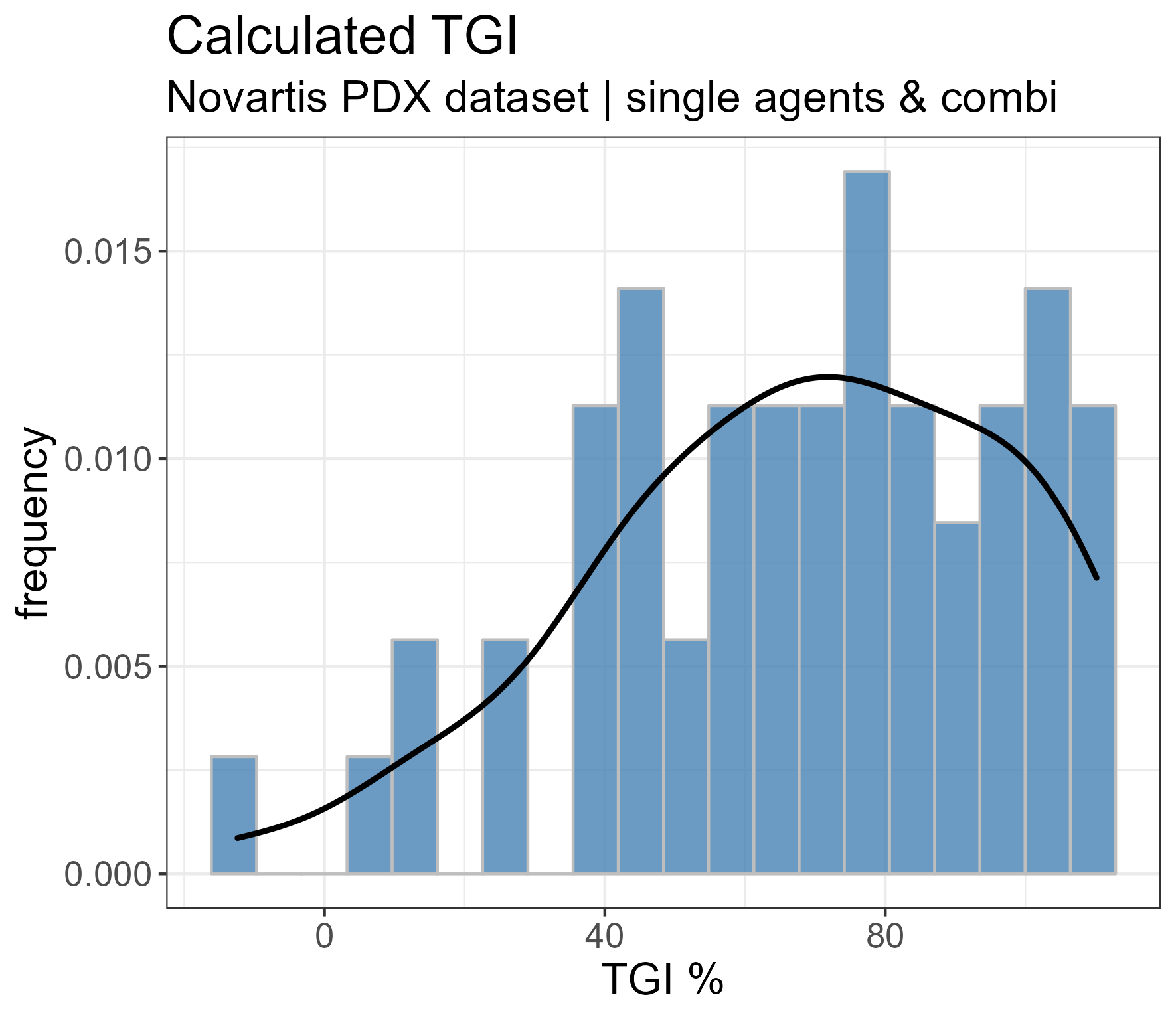

Supplement: Supplementary file 1 [file DataSheet1.ZIP › code_complete/results_TGI_combi_2/calc_TGI_all_hist.png]

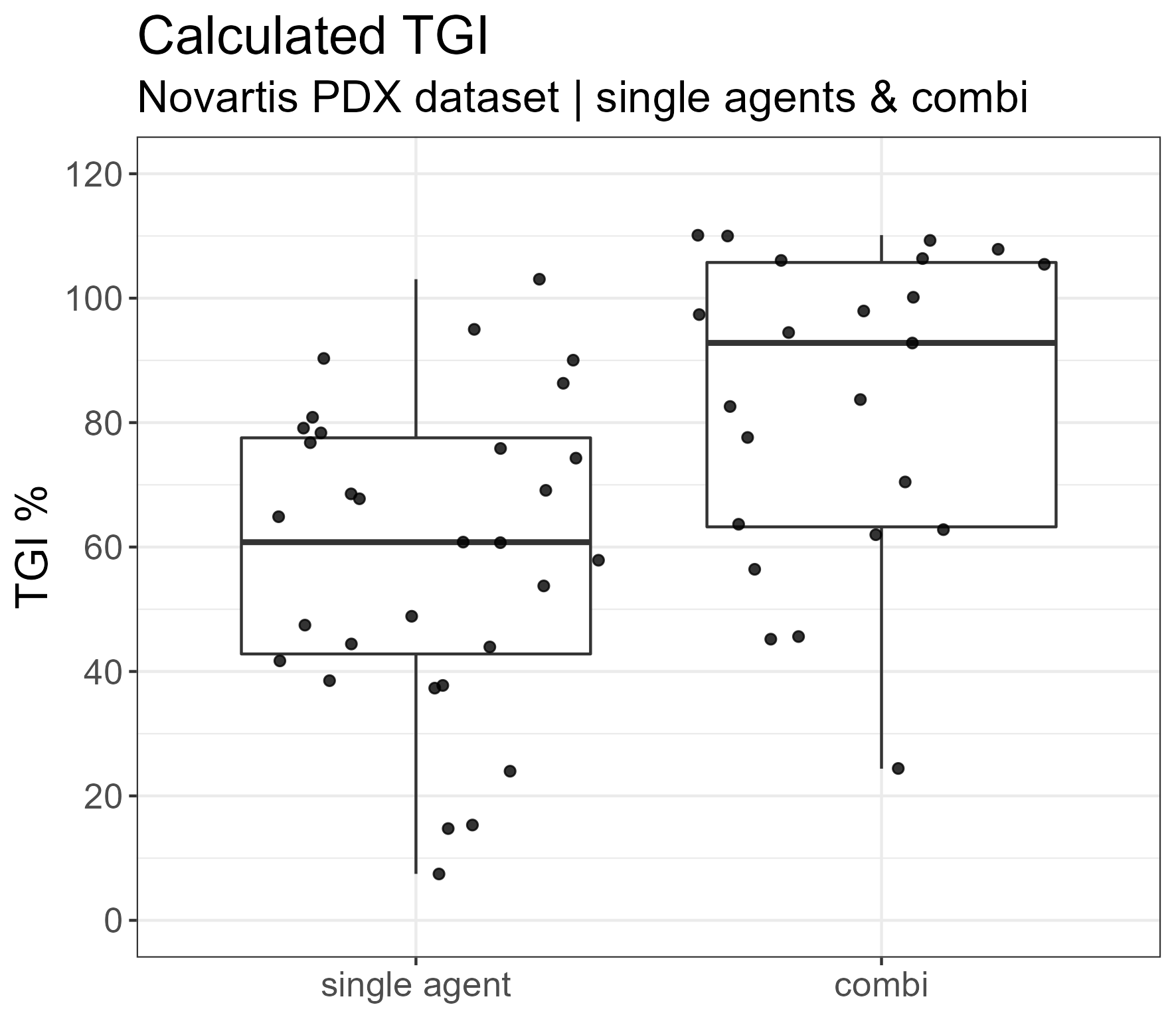

Supplement: Supplementary file 1 [file DataSheet1.ZIP › code_complete/results_TGI_combi_2/calc_TGI_strat.png]

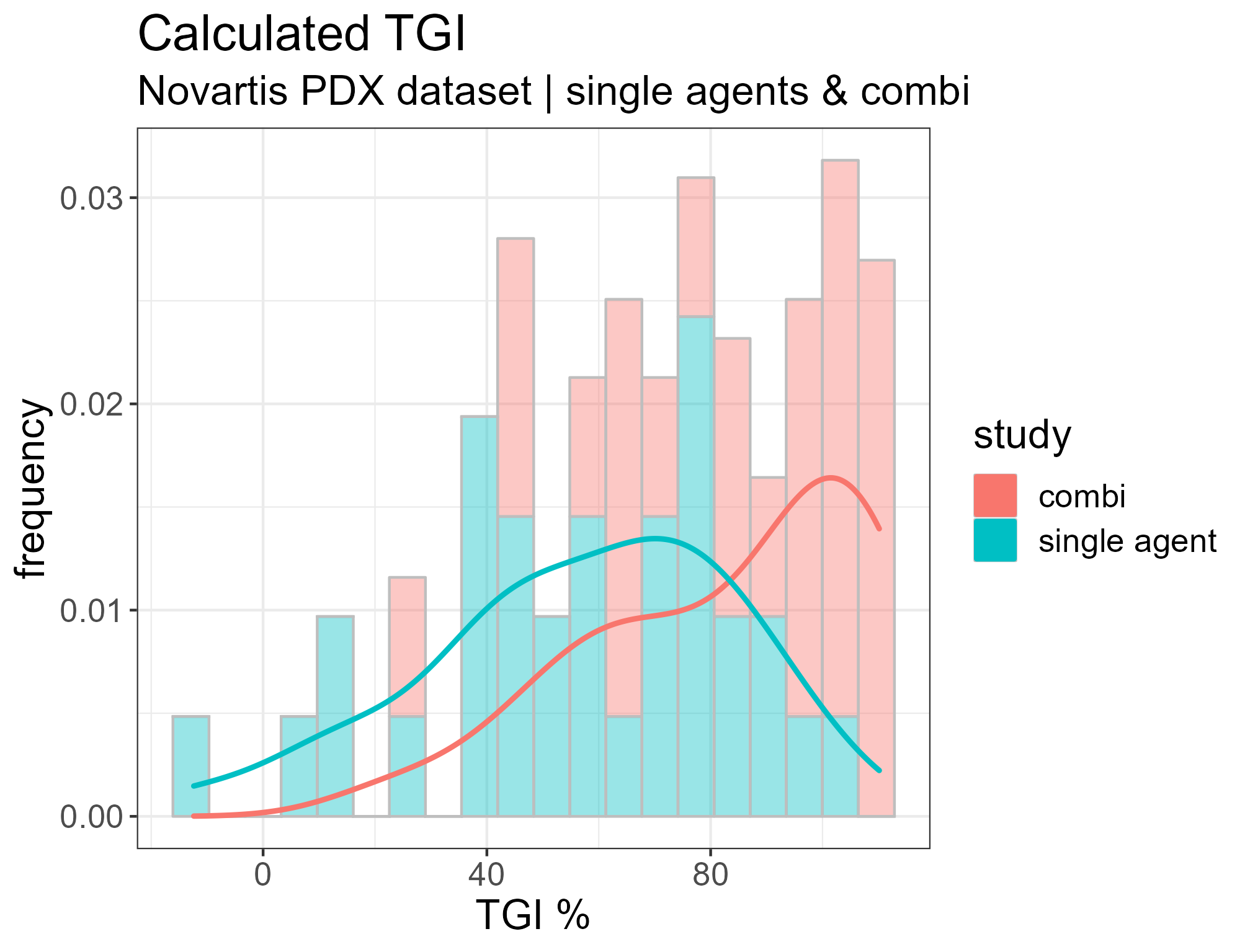

Supplement: Supplementary file 1 [file DataSheet1.ZIP › code_complete/results_TGI_combi_2/calc_TGI_strat_hist.png]

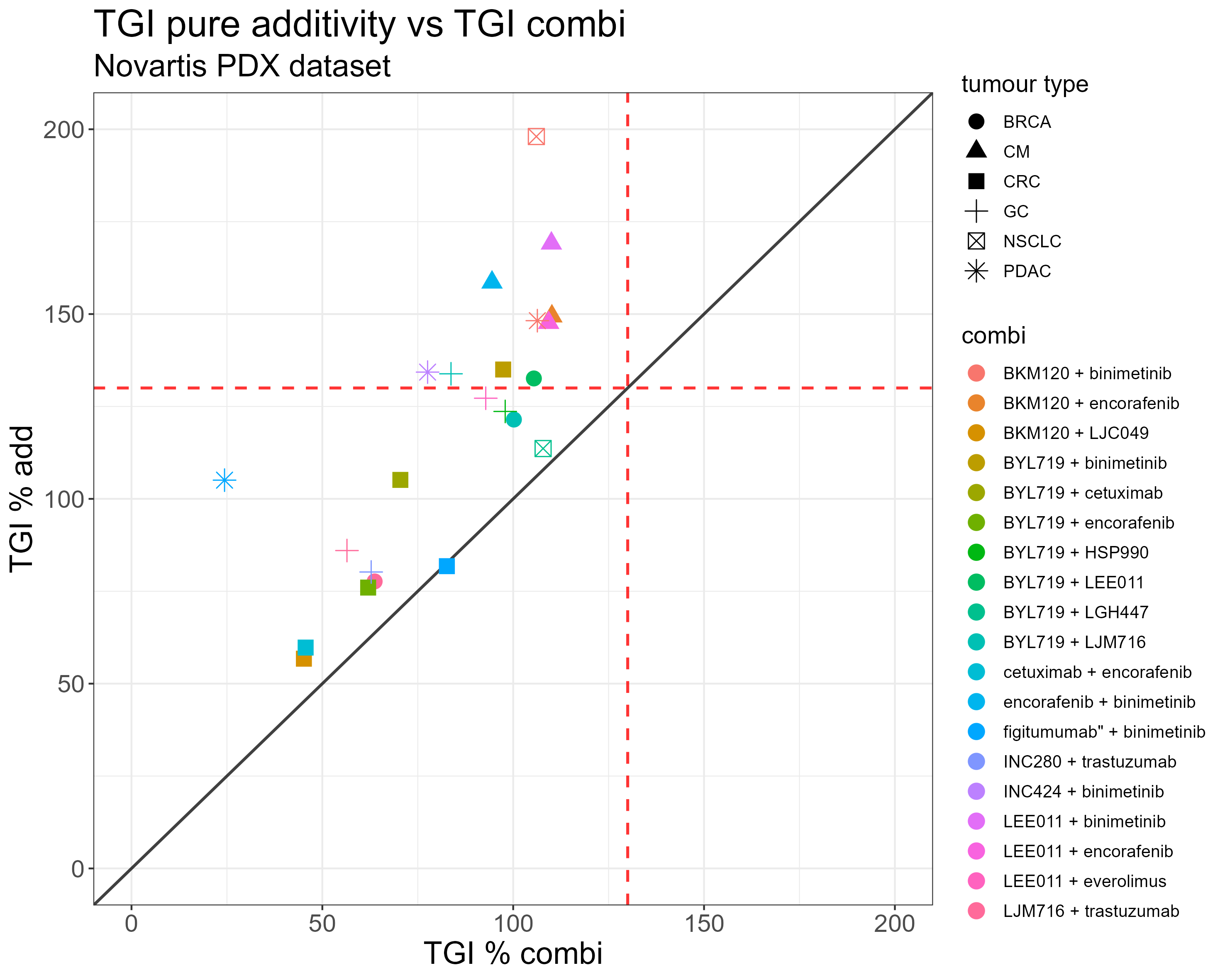

Supplement: Supplementary file 1 [file DataSheet1.ZIP › code_complete/results_TGI_combi_2/TGI_add_vs_TGI_combi.png]

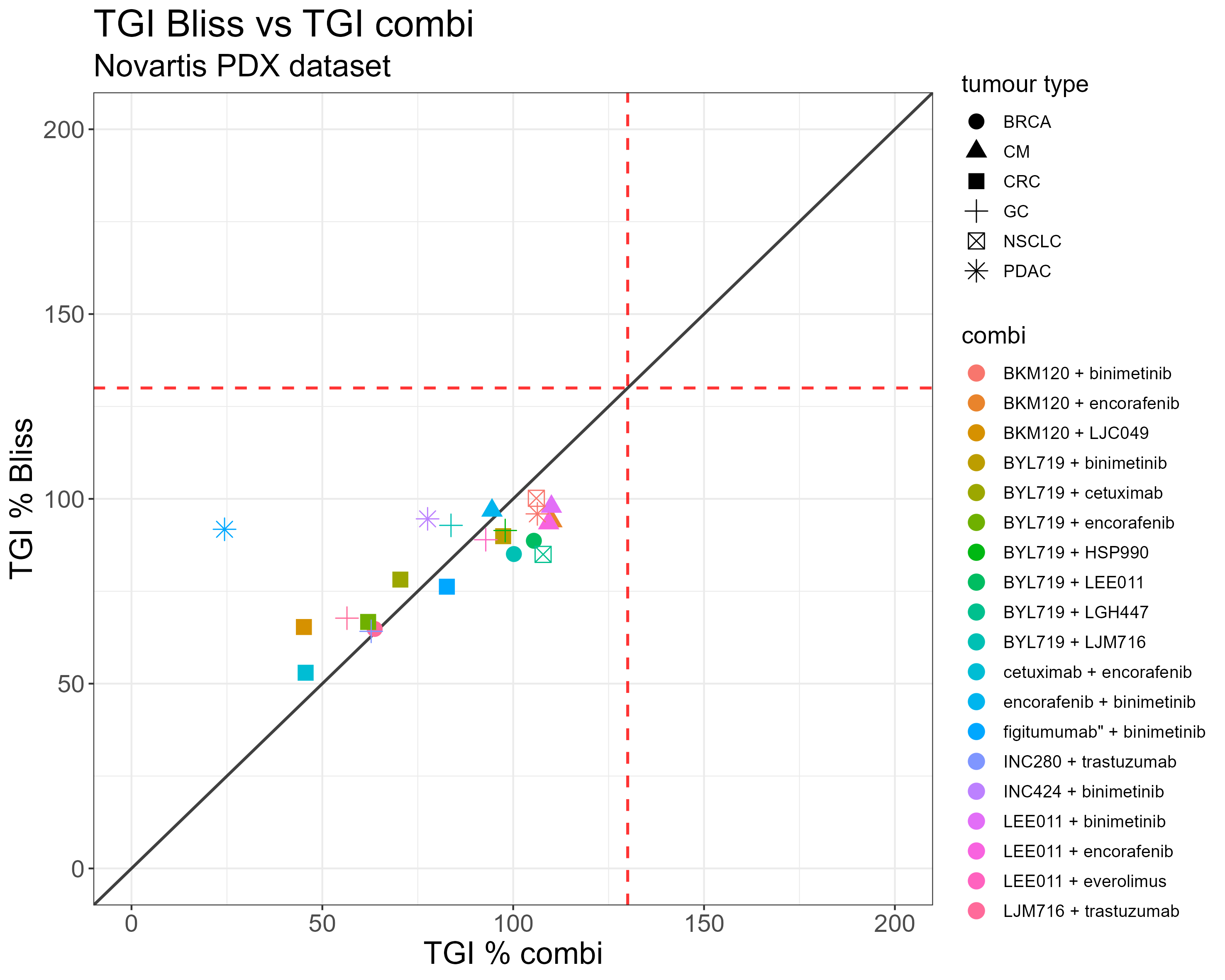

Supplement: Supplementary file 1 [file DataSheet1.ZIP › code_complete/results_TGI_combi_2/TGI_Bliss_vs_TGI_combi.png]

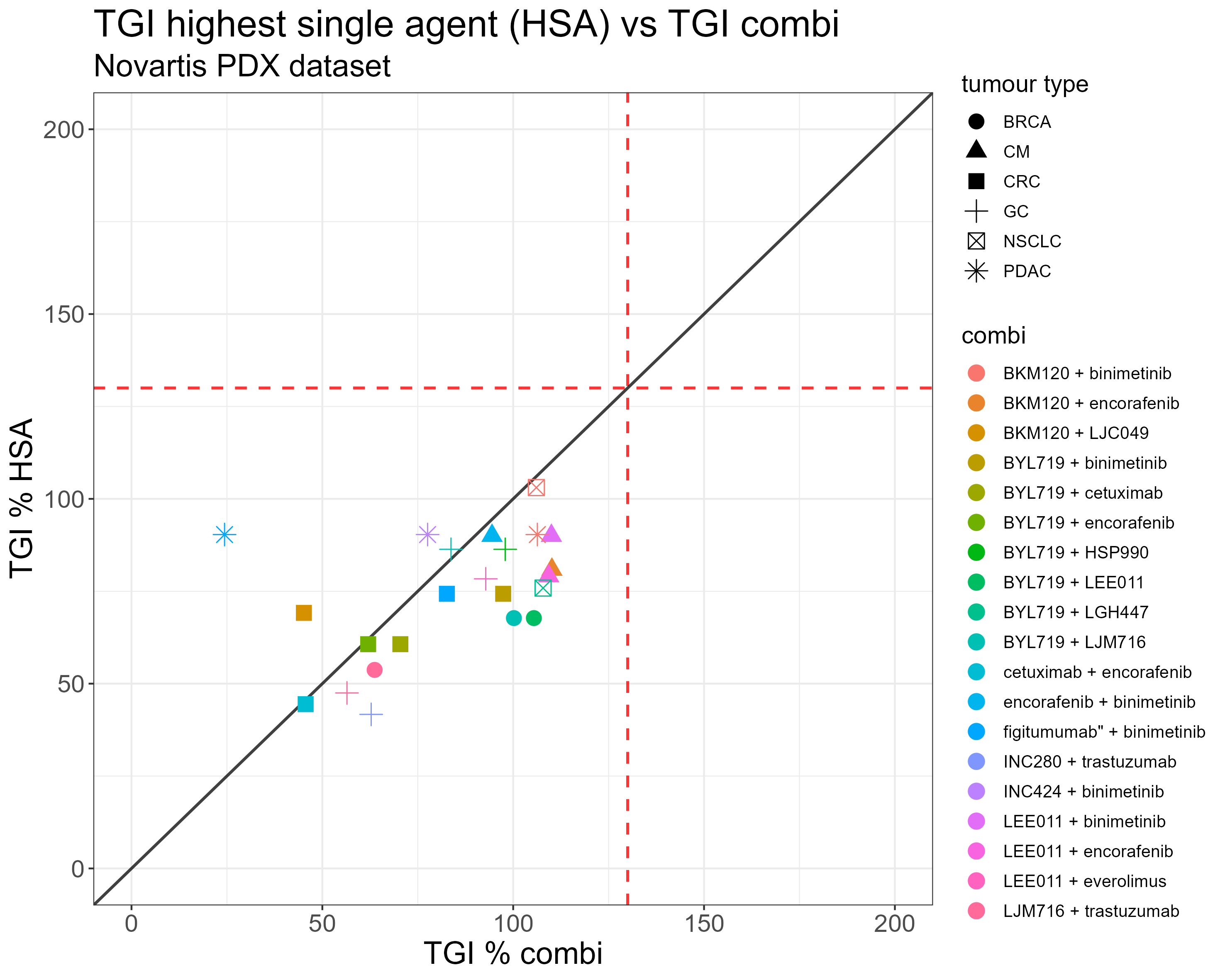

Supplement: Supplementary file 1 [file DataSheet1.ZIP › code_complete/results_TGI_combi_2/TGI_HSA_vs_TGI_combi.png]

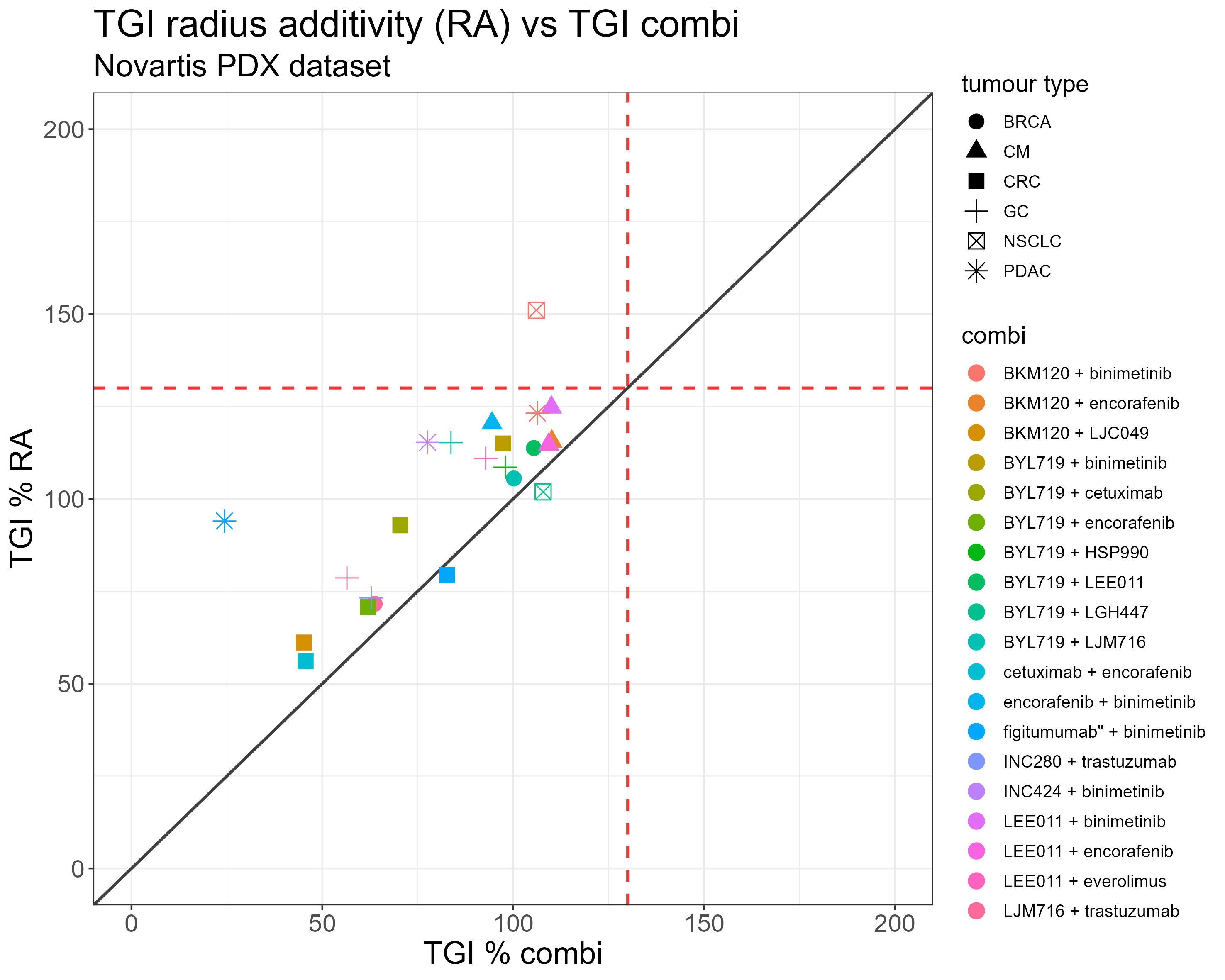

Supplement: Supplementary file 1 [file DataSheet1.ZIP › code_complete/results_TGI_combi_2/TGI_RA_vs_TGI_combi.png]

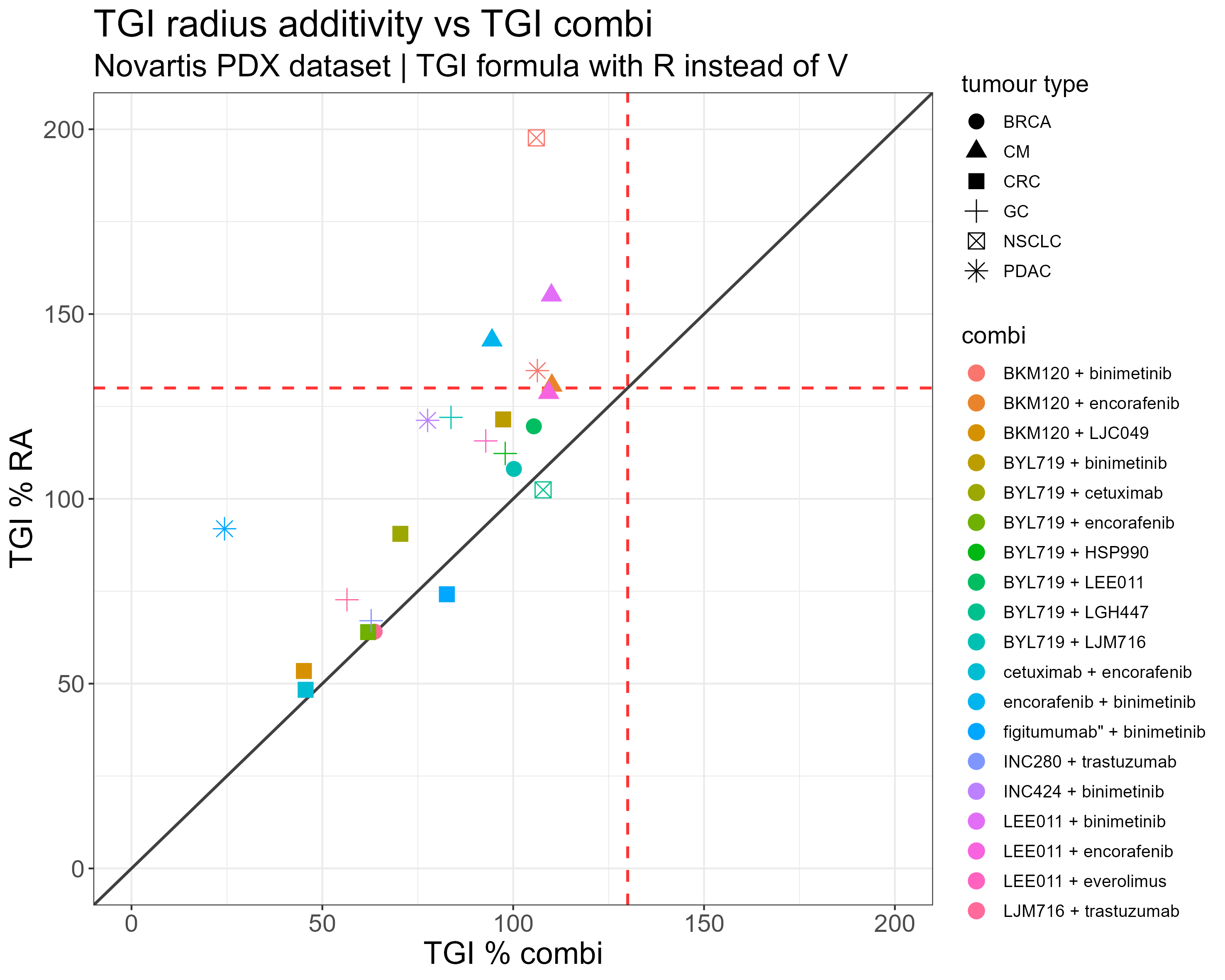

Supplement: Supplementary file 1 [file DataSheet1.ZIP › code_complete/results_TGI_combi_2/TGI_RA_vs_TGI_combi_R_formula.png]

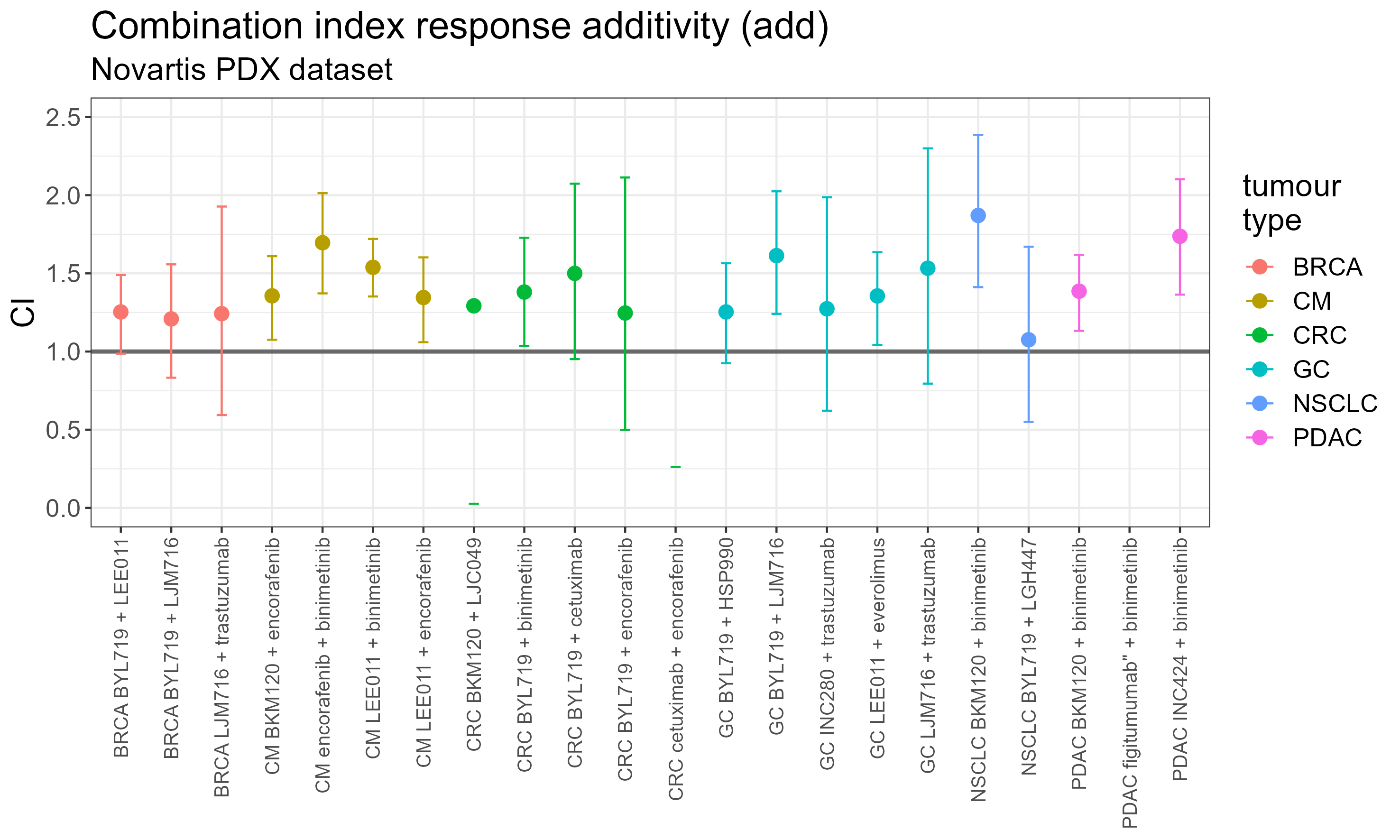

Supplement: Supplementary file 1 [file DataSheet1.ZIP › code_complete/results_TGI_combi_2_boot/CI_add.png]

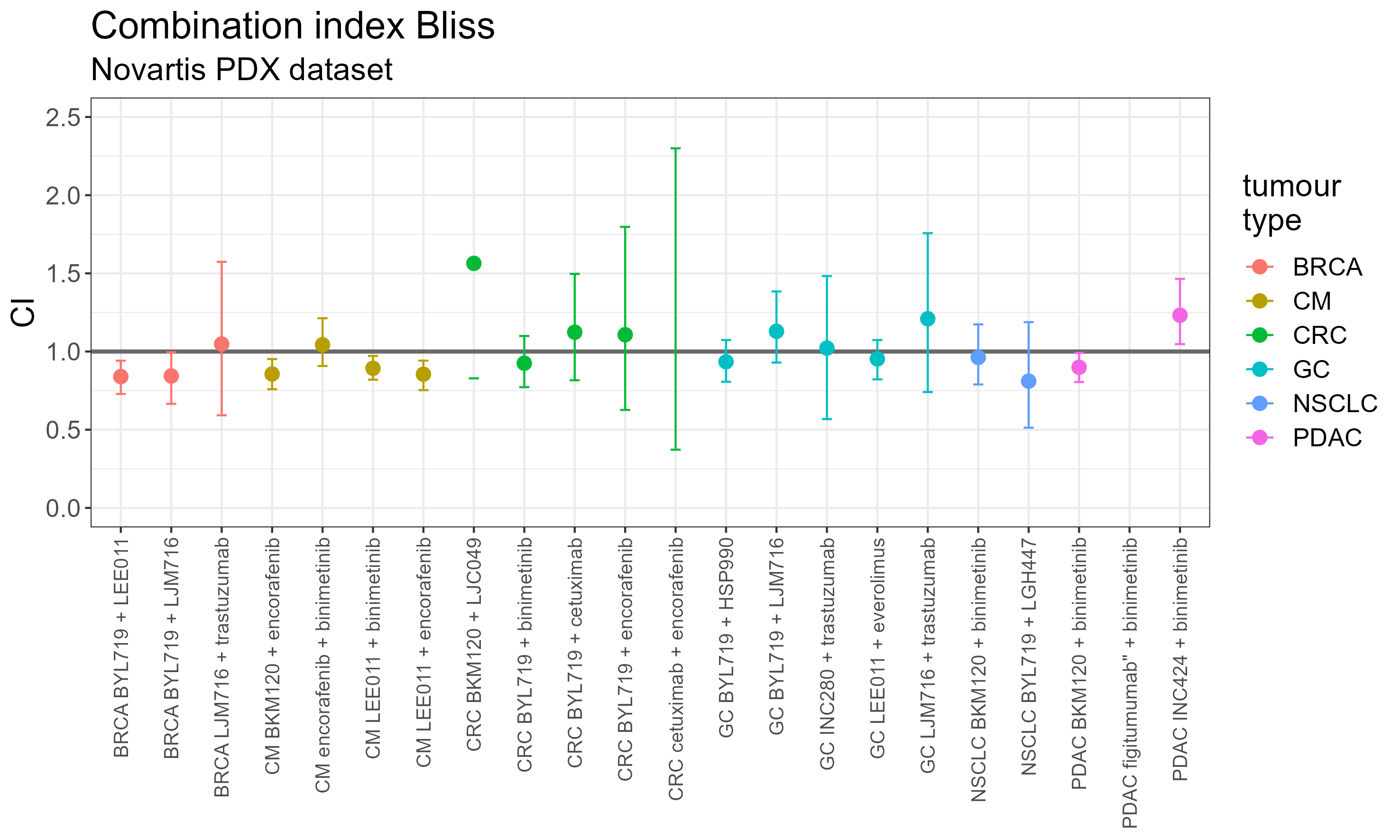

Supplement: Supplementary file 1 [file DataSheet1.ZIP › code_complete/results_TGI_combi_2_boot/CI_Bliss.png]

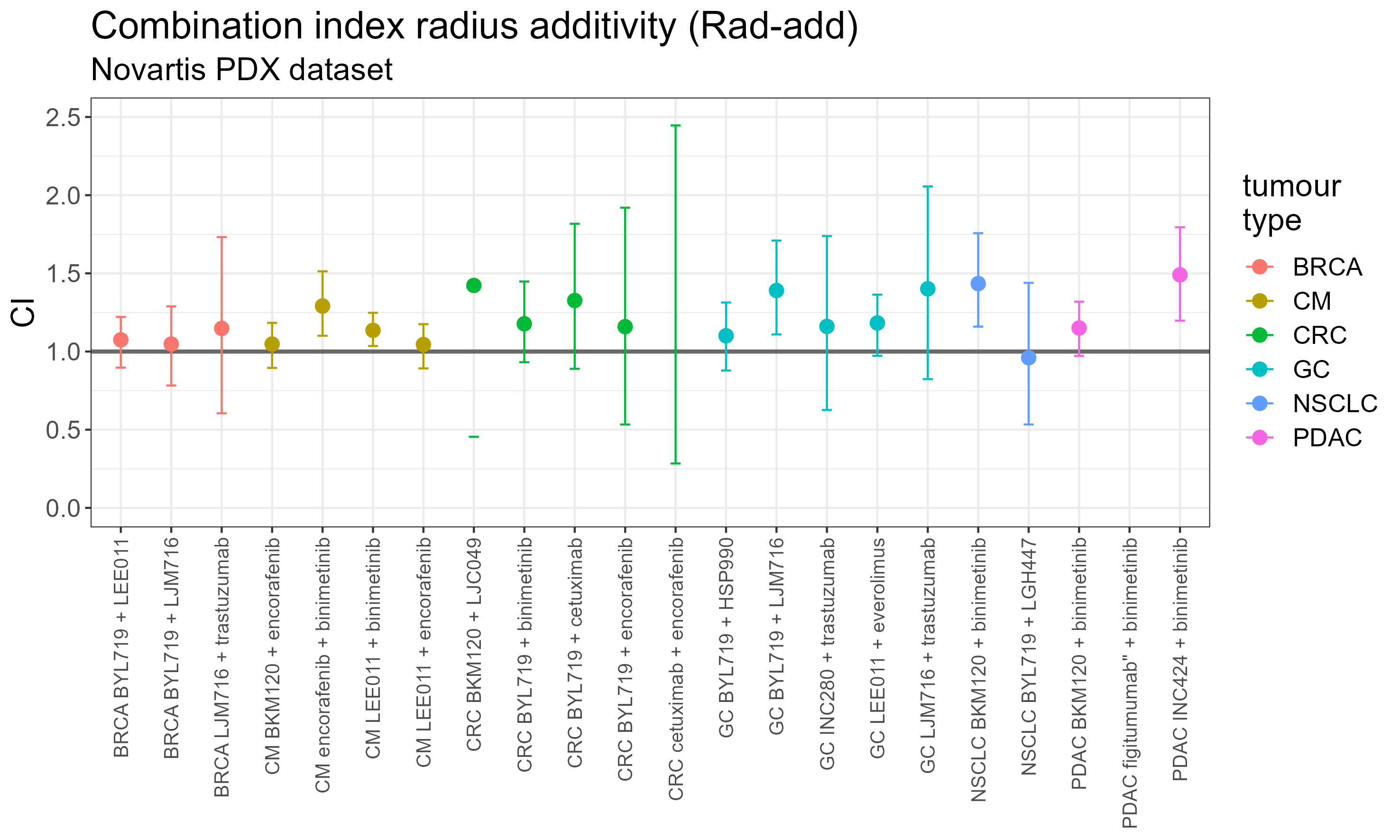

Supplement: Supplementary file 1 [file DataSheet1.ZIP › code_complete/results_TGI_combi_2_boot/CI_RA.png]

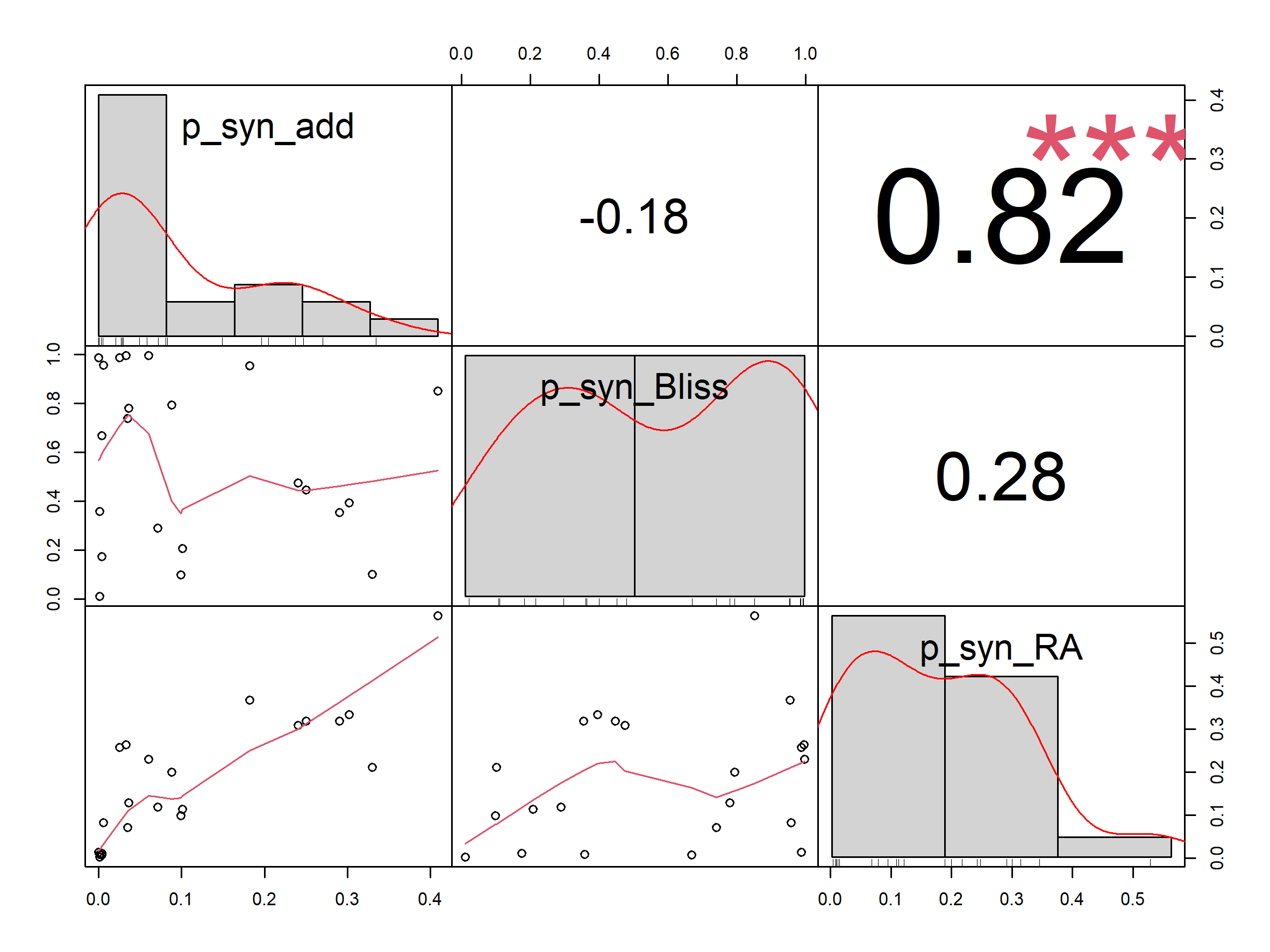

Supplement: Supplementary file 1 [file DataSheet1.ZIP › code_complete/results_TGI_combi_2_boot/corr_prob.jpg]

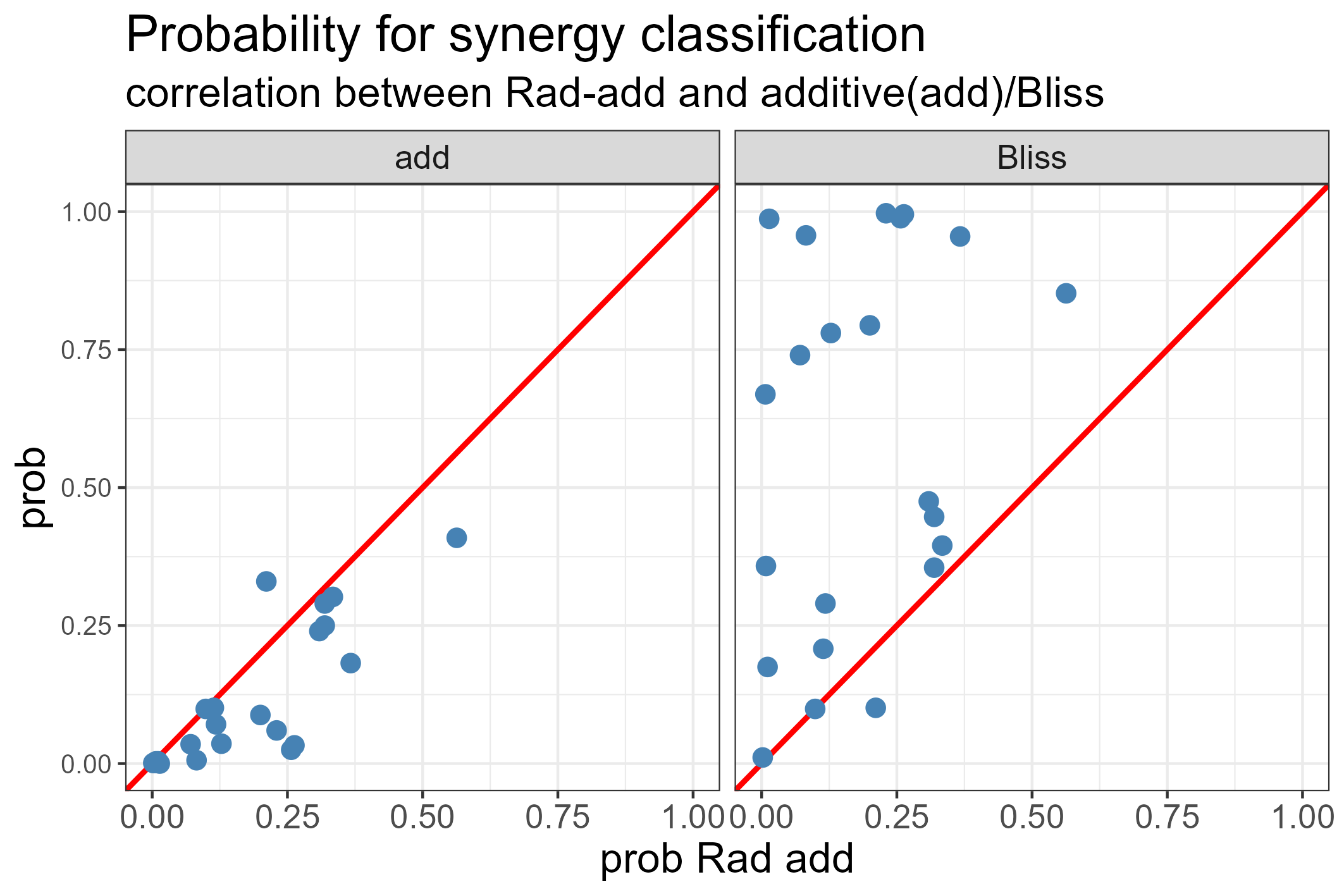

Supplement: Supplementary file 1 [file DataSheet1.ZIP › code_complete/results_TGI_combi_2_boot/corr_RA_Bliss_add.png]

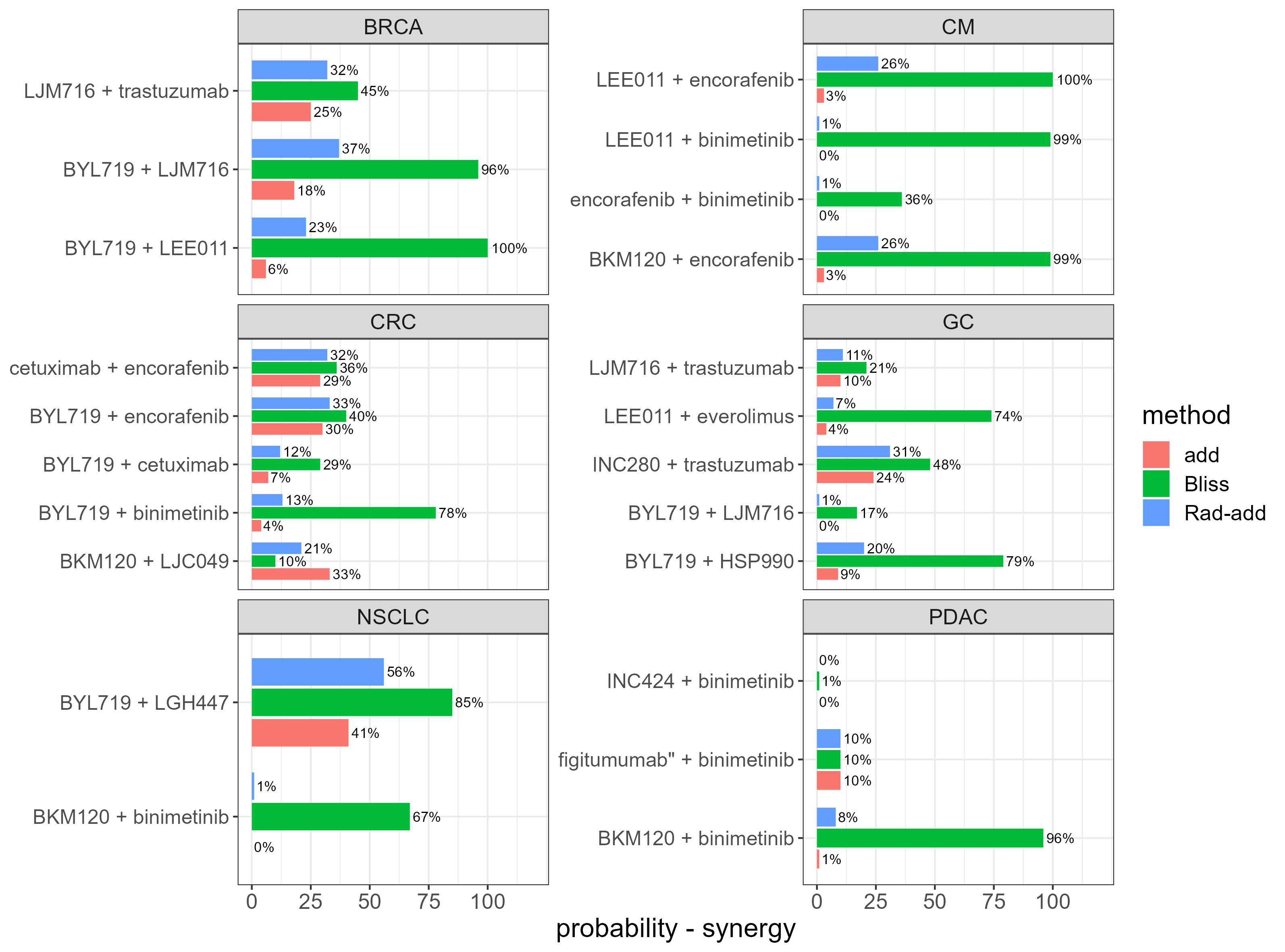

Supplement: Supplementary file 1 [file DataSheet1.ZIP › code_complete/results_TGI_combi_2_boot/prob_2.png]

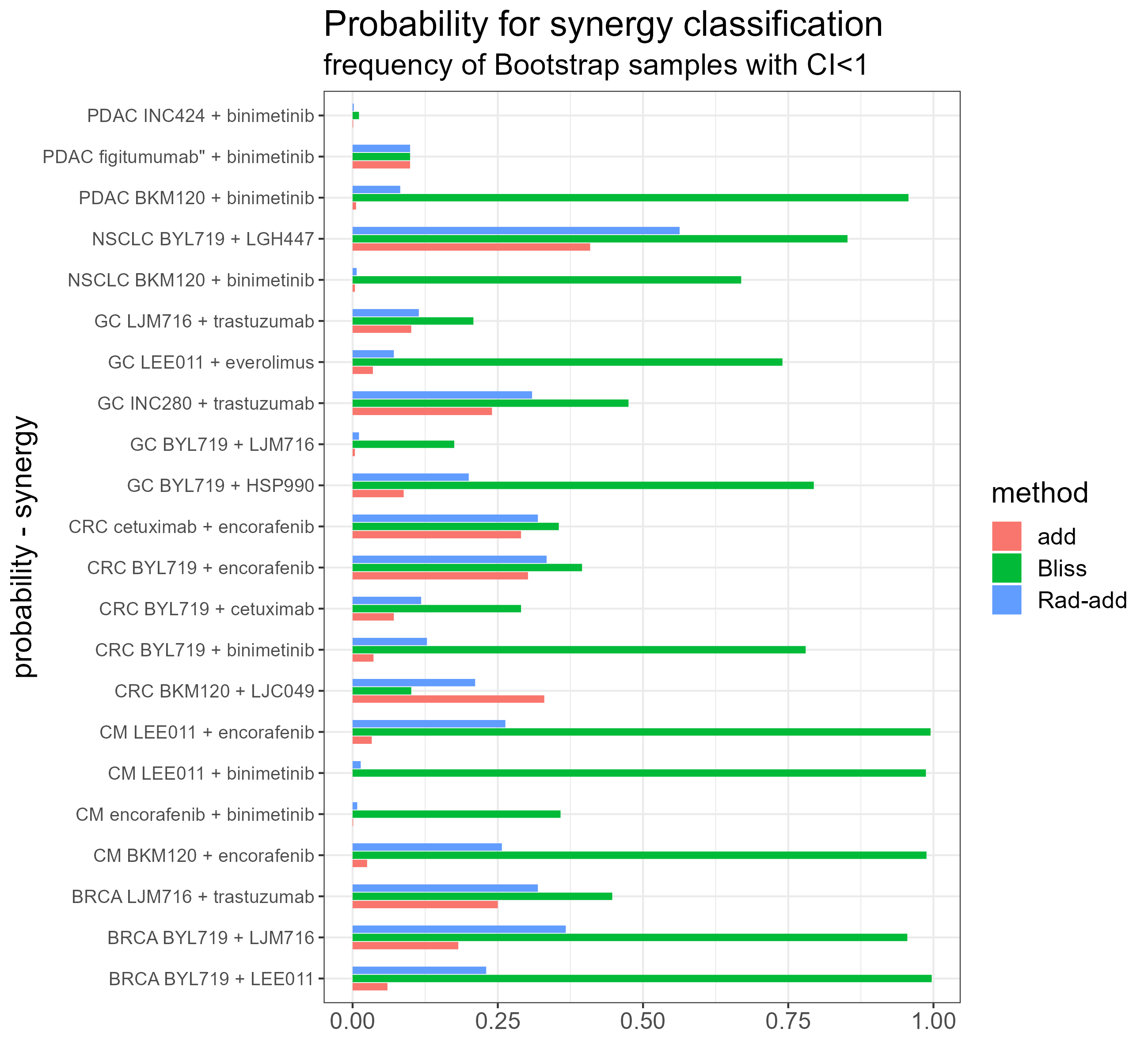

Supplement: Supplementary file 1 [file DataSheet1.ZIP › code_complete/results_TGI_combi_2_boot/prob_synergy.png]
