# Supplementary material for: Radius additivity score: a novel combination index for tumour growth inhibition in fixed-dose xenograft studies
Source: Front Pharmacol. 2023 Oct 13;14:1272058. doi: 10.3389/fphar.2023.1272058 (PMC10603293; doi:10.3389/fphar.2023.1272058)
Supplement: Supplementary file 2 [file DataSheet1.docx]

# Supplementary materials

Table S1: predicted combination indices (CI) using response additivity (add), Bliss and radius additivity (Rad-add) approaches on combination studies with BRCA, CM and CRC as tumour types.

| **Tumour type** | **Drug 1** | **Drug 2** | **CI add**  **90% CI** | **Prob synergy**  **add** | **CI Bliss**  **90% CI** | **Prob synergy Bliss** | **CI Rad-add**  **90% CI** | **Prob synergy**  **Rad-add** |
| --- | --- | --- | --- | --- | --- | --- | --- | --- |
| BRCA | BYL719 | LEE011 | 1.25  (0.99, 1.49) | 0.06 | 0.84  (0.73, 0.94) | 1 | 1.07  (0.9, 1.22) | 0.23 |
| BRCA | BYL719 | LJM716 | 1.21  (0.83, 1.56) | 0.18 | 0.84  (0.67, 1) | 0.96 | 1.05  (0.78, 1.29) | 0.37 |
| BRCA | LJM716 | trastuzumab | 1.24  (0.59, 1.93) | 0.25 | 1.05  (0.59, 1.57) | 0.45 | 1.15  (0.6, 1.73) | 0.32 |
| CM | BKM120 | encorafenib | 1.36  (1.08, 1.61) | 0.03 | 0.86  (0.76, 0.95) | 0.99 | 1.05  (0.9, 1.18) | 0.26 |
| CM | encorafenib | binimetinib | 1.7  (1.37, 2.01) | 0 | 1.04  (0.91, 1.21) | 0.36 | 1.29  (1.1, 1.51) | 0.01 |
| CM | LEE011 | binimetinib | 1.54  (1.35, 1.72) | 0 | 0.89  (0.82, 0.97) | 0.99 | 1.14  (1.04, 1.25) | 0.01 |
| CM | LEE011 | encorafenib | 1.35  (1.06, 1.6) | 0.03 | 0.85  (0.75, 0.94) | 1 | 1.04  (0.89, 1.18) | 0.26 |
| CRC | BKM120 | LJC049 | 1.29  (0.03, 2.58) | 0.33 | 1.56  (0.83, 2.71) | 0.1 | 1.42  (0.45, 2.56) | 0.21 |
| CRC | BYL719 | binimetinib | 1.38  (1.04, 1.73) | 0.04 | 0.93  (0.77, 1.1) | 0.78 | 1.18  (0.93, 1.45) | 0.13 |
| CRC | BYL719 | cetuximab | 1.5  (0.95, 2.07) | 0.07 | 1.12  (0.82, 1.5) | 0.29 | 1.33  (0.89, 1.82) | 0.12 |
| CRC | BYL719 | encorafenib | 1.25  (0.5, 2.11) | 0.3 | 1.11  (0.63, 1.8) | 0.4 | 1.16  (0.53, 1.92) | 0.33 |
| CRC | cetuximab | encorafenib | -0.87  (0.26, 2.65) | 0.29 | -0.72  (0.37, 2.3) | 0.36 | -1.1  (0.28, 2.45) | 0.32 |

Table S2: predicted combination indices (CI) using response additivity (add), Bliss and radius additivity (Rad-add) approaches on combination studies with GC, NSCLC and PDAC as tumour types.

| **Tumour type** | **Drug 1** | **Drug 2** | **CI add**  **90% CI** | **Prob synergy**  **add** | **CI Bliss**  **90% CI** | **Prob synergy Bliss** | **CI Rad-add**  **90% CI** | **Prob synergy**  **Rad-add** |
| --- | --- | --- | --- | --- | --- | --- | --- | --- |
| GC | BYL719 | HSP990 | 1.25  (0.93, 1.56) | 0.09 | 0.93  (0.81, 1.07) | 0.79 | 1.1  (0.88, 1.31) | 0.2 |
| GC | BYL719 | LJM716 | 1.61  (1.24, 2.03) | 0 | 1.13  (0.93, 1.38) | 0.17 | 1.39  (1.11, 1.71) | 0.01 |
| GC | INC280 | trastuzumab | 1.27  (0.62, 1.99) | 0.24 | 1.02  (0.57, 1.48) | 0.48 | 1.16  (0.63, 1.74) | 0.31 |
| GC | LEE011 | everolimus | 1.36  (1.04, 1.64) | 0.04 | 0.95  (0.82, 1.07) | 0.74 | 1.18  (0.97, 1.36) | 0.07 |
| GC | LJM716 | trastuzumab | 1.53  (0.79, 2.3) | 0.1 | 1.21  (0.74, 1.76) | 0.21 | 1.4  (0.82, 2.06) | 0.11 |
| NSCLC | BKM120 | binimetinib | 1.87  (1.41, 2.39) | 0 | 0.96  (0.79, 1.17) | 0.67 | 1.43  (1.16, 1.76) | 0.01 |
| NSCLC | BYL719 | LGH447 | 1.08  (0.55, 1.67) | 0.41 | 0.81  (0.51, 1.19) | 0.85 | 0.96  (0.53, 1.44) | 0.56 |
| PDAC | BKM120 | binimetinib | 1.39  (1.13, 1.62) | 0.01 | 0.9  (0.81, 0.99) | 0.96 | 1.15  (0.97, 1.32) | 0.08 |
| PDAC | figitumumab" | binimetinib | 4.2  (-10.72, 20.56) | 0.1 | 2.68  (-10.21, 18.5) | 0.1 | 3.38  (-9.76, 18.66) | 0.1 |
| PDAC | INC424 | binimetinib | 1.74  (1.36, 2.1) | 0 | 1.23  (1.05, 1.46) | 0.01 | 1.49  (1.2, 1.79) | 0 |


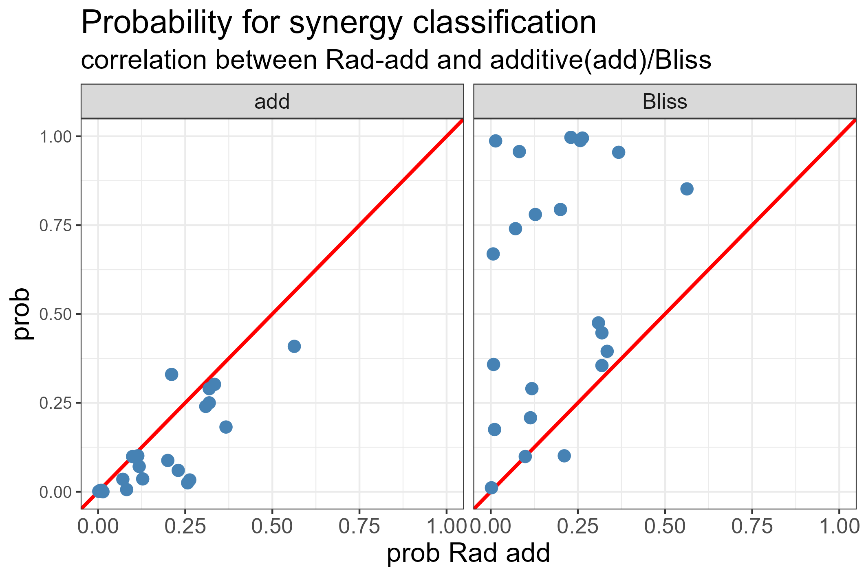


Figure S1: For each combination study, this figure reports the predicted probability of being classified as synergistic using the response additivity (add) and bliss methods vs. those using the radius additivity (Rad-add) approach. The response additivity predicted probabilities correlated well with the radius additivity probabilities (ρ=0.82, p<0.01), while the probabilities predicted using the Bliss approach did not (ρ=0.28, p=0.2).


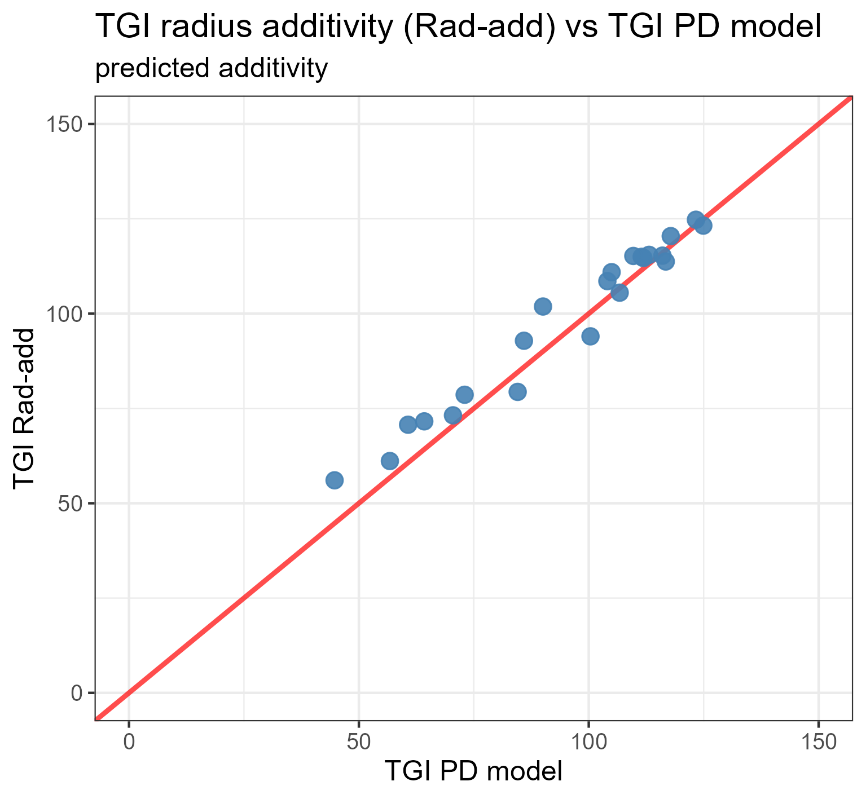


Figure S2: In this figure, for each combination, the predicted TGI in the case of additivity using the radius additivity (Rad-add) approach are reported against those predicted with the constant radius growth model-based analysis. In this exercise, all available curves were used to calculate the TGI with both approaches. The two predicted TGIs correlated well (ρ=0.98, p<0.01), and the maximal absolute difference between the two TGI, calculated as max(| TGI_Rad-add_ – TGI_PD model_ |), was 11.8%.
